# Cranial anatomy of *Allosaurus jimmadseni*, a new species from the lower part of the Morrison Formation (Upper Jurassic) of Western North America

**DOI:** 10.7717/peerj.7803

**Published:** 2020-01-24

**Authors:** Daniel J. Chure, Mark A. Loewen

**Affiliations:** 1 Dinosaur National Monument (retired), Jensen, UT, USA; 2 Independent Researcher, Jensen, UT, USA; 3 Natural History Museum of Utah, University of Utah, Salt Lake City, UT, USA; 4 Department of Geology and Geophysics, University of Utah, Salt Lake City, UT, USA

**Keywords:** Allosaurus, *Allosaurus jimmadseni*, Dinosaur, Theropod, Morrison Formation, Jurassic, Cranial anatomy

## Abstract

*Allosaurus* is one of the best known theropod dinosaurs from the Jurassic and a crucial taxon in phylogenetic analyses. On the basis of an in-depth, firsthand study of the bulk of *Allosaurus* specimens housed in North American institutions, we describe here a new theropod dinosaur from the Upper Jurassic Morrison Formation of Western North America, *Allosaurus jimmadseni* sp. nov., based upon a remarkably complete articulated skeleton and skull and a second specimen with an articulated skull and associated skeleton. The present study also assigns several other specimens to this new species, *Allosaurus jimmadseni*, which is characterized by a number of autapomorphies present on the dermal skull roof and additional characters present in the postcrania. In particular, whereas the ventral margin of the jugal of *Allosaurus fragilis* has pronounced sigmoidal convexity, the ventral margin is virtually straight in *Allosaurus jimmadseni*. The paired nasals of *Allosaurus jimmadseni* possess bilateral, blade-like crests along the lateral margin, forming a pronounced nasolacrimal crest that is absent in *Allosaurus fragilis*.

## Introduction

*Allosaurus* is the most common genus of theropod in the Late Jurassic of North America. It is widespread both geographically and stratigraphically and the most abundant theropod in virtually all quarries (Turner & Peterson, 1999; Foster, 2003). Nonetheless, well-preserved complete skeletons are rare, and most occurrences are represented by scattered elements. A major concentration of *Allosaurus* material is preserved in the Cleveland-Lloyd Dinosaur Quarry, where disassociated bones of dozens of individuals over a wide ontogenetic range occur by the thousands (Madsen, 1976; Miller, Horrocks & Madsen, 1996).

*Allosaurus* has long played a crucial role in phylogenetic analyses of the Theropoda, either as a member of an ingroup or as an outgroup taxon in analyses of Coelurosauria (Rauhut, 2003; Benson, Carrano & Brusatte, 2010; Carrano, Benson & Sampson, 2012). Nineteen species of *Allosaurus* have been erected since 1877 (see Chure, 2000a; Mateus, Walen & Antunes, 2006; Dalman, 2014), although the holotype material for many has been neither fully illustrated nor described, and the validity of these species has not been critically evaluated. Many proposed synonymies are yet to be evaluated in detail, although we have a manuscript in preparation doing that. As the holotype of *Allosaurus fragilis* is not diagnostic, a neotype has been proposed in an International Commission on Zoological Nomenclature (ICZN) (Case 3506) (Paul & Carpenter, 2010; Carrano, Loewen & Evers, 2018) to conserve the name *Allosaurus*. In the past MOR 693 has been the subject of studies on pathology (Hanna, 2002) and morphology (Rayfield, 2005a, 2005b) and considered *Allosaurus fragilis*. This study refers this specimen to *Allosaurus jimmadseni*. We currently recognize only three species in genus *Allosaurus*: *Allosaurus fragilis* and *Allosaurus jimmadseni* in North America and *Allosaurus europaeus* in Europe.

Over the past 20 years, the authors have conducted a hands-on, detailed morphological study of virtually all North American *Allosaurus* material, including several new and remarkably complete specimens that shed important light on the morphology of this dinosaur. Given the abundance of data we now possess on *Allosaurus*, we will present our analyses over a series of publications; this present study describing skull morphology, is the first. A postcranial description and a revision of genus *Allosaurus* will be the subject of a future publication.

### Discovery and excavational history

Here we describe two specimens of *Allosaurus* from the lower part of the Morrison Formation: DINO 11541 from Dinosaur National Monument of Utah and MOR 693 from the Howe Quarry in Wyoming. DINO 11541 was found by Dr. George Engelmann (University of Nebraska, Omaha) on July 15, 1990 (Hubert & Chure, 1992) during a contracted paleontological inventory of the Morrison Formation of Dinosaur National Monument (National Park Service contract CA-1463-5-0001). The surface material consisted of several articulated pedal phalanges of the right pes and several articulated midcaudal vertebrae. The specimen was located about six m off the ground in a sandstone face dipping approximately 70° south. Excavation of DINO 11541 by staff of the National Park Service’s Dinosaur National Monument started in the late summer of 1990 and continued through the summer of 1994. The tilt of the beds and the weight of the block required the judicious use of explosives to remove overburden and the development of innovative solutions to getting the block horizontal on a palette (Elder & Madsen, 1994; Elder, Madsen & Chure, 1994, 1997). The postcranial skeleton was jacketed primarily in a single 2,700 kg block and flown out by helicopter (Chure, 2000a).

**Figure 1:**
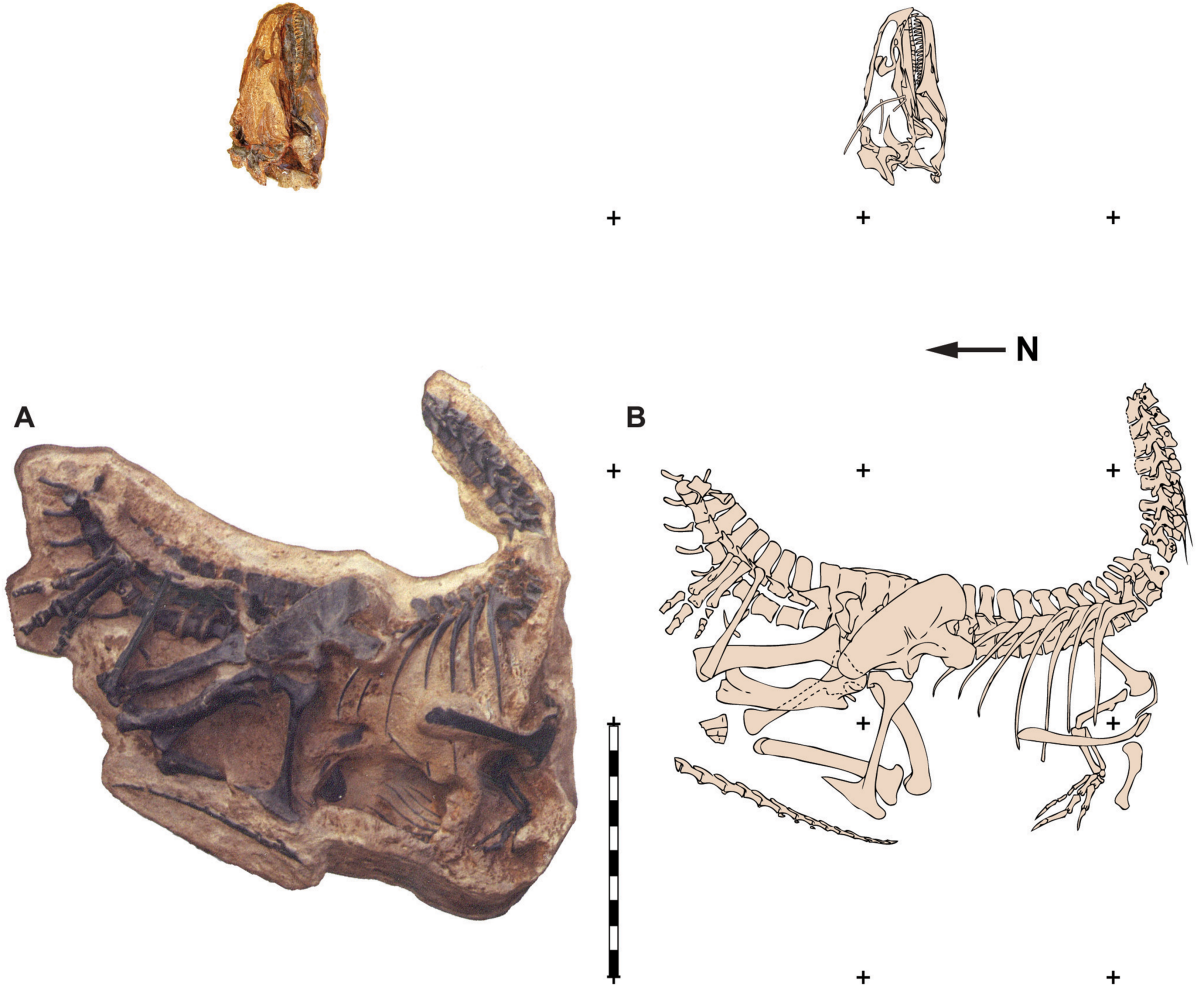
Quarry map of DINO 11541. Photograph of a painted cast of parts of the skeleton and skull of DINO 11541 in their original positions with respect to each other (A) and an explanatory line drawing taken from original quarry photos (B). Photos by Dan Chure. Scale bar equals one m.

After the articulated and nearly complete postcranium was removed, excavation continued for another 2 weeks in an attempt to find the skull, but work ceased when the quarry wall became vertical and there was no sign of it. During the summer of 1996, Ray Jones of the University of Utah came to the monument and used his recently developed radiological surveying techniques to locate a high gamma emission source in the quarry wall. Excavations began again and the skull was found just below the surface (Jones & Chure, 1998; Jones, McDonald & Chure, 1998a, 1998b, 1998c). The spatial relationship between the skull and skeleton are shown in Fig. 1. Collection of the skull was completed in 1996 and the DINO 11541 was prepared by Scott Madsen and Ann Elder at Dinosaur National Monument during 1996 and 1997 (Chure, 2000a).

**Figure 2:**
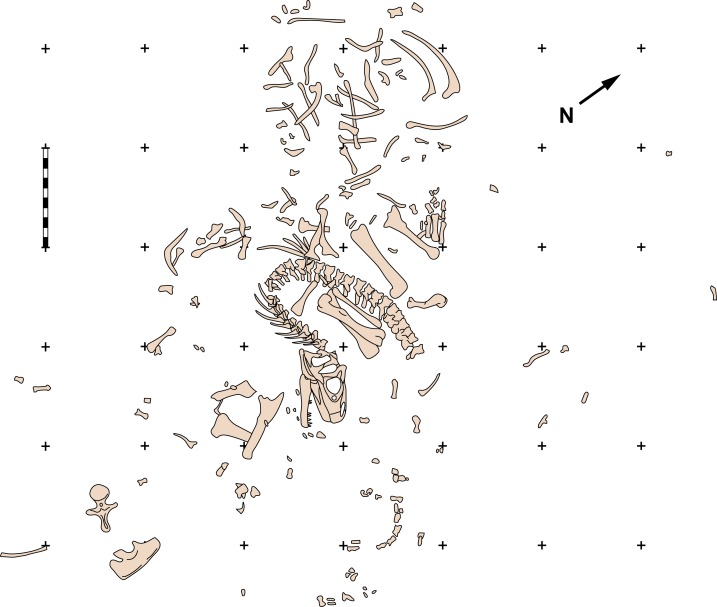
Quarry map of MOR 693. Quarry map of recovered elements of Big Al (MOR 693) from the Howe Quarry near Shell, Wyoming. Scale bars equals one m. Redrawn with permission from original artwork by Scott Hartman. Scale bar equals one m.

In 1934, Barnum Brown and a field crew from the American Museum of Natural History collected over 30 tons of sauropod bones from the Howe Ranch Quarry near Shell, Wyoming (Brown, 1935; Colbert, 1968). Brown’s field crews excavated remains of multiple sauropods—including *Barosaurus*, *Diplodocus*, *Apatosaurus*, and *Camarasaurus*—along with elements of the ornithopod *Camptosaurus* (Ayer, 1999). The only theropod remains recovered during this period were of *Allosaurus*. During the 1990s a commercial fossil collecting company Siber + Siber, Ltd., from Switzerland began digging at the Howe Quarry, located on private land adjacent to land administered by the U. S. Bureau of Land Management (BLM). During this effort, the commercial company found limited numbers of specimens in the original Howe Quarry and subsequently began to prospect nearby for sites nearby (Ayer, 1999). In 1991 they discovered an associated *Allosaurus* skeleton that became known as “Big Al.” The skull was still articulated with the axial column and much of the skeleton itself was in articulation (Fig. 2). Thereafter, the BLM recognized that this new site was located on public land and the excavation of the specimen (MOR 693) was taken over by a field crew from the Museum of the Rockies in Bozeman, Montana (Breithaupt, 1996). Undeterred, the Swiss found another, slightly larger individual (SMA 0005) on private land at Howe Ranch and dubbed it “Big Al II” (Ayer, 1999; Foth et al., 2015). This second Howe Ranch Quarry *Allosaurus* is housed in the Saurier Museum of Atahal in Switzerland. SMA 0005 is currently being described by scientists at Ludwig-Maximilians-University in Munich, Germany. Pathonogies in this specimen were described by Foth et al. (2015).

MOR 693 was prepared between 1991 and 1995 by crews from the Museum of the Rockies in Bozeman, Montana and has been the subject of several papers including studies of pathology (Laws, 1993, 1995, 1996, 1997; Hanna, 2002), cranial strength (Rayfield, 2005a, 2005b; Rayfield et al., 2001) and neck strength associated with feeding (Snively et al., 2013). All but one (Snively et al., 2013) of these studies have considered MOR 693 a specimen of *Allosaurus fragilis* and no detailed descriptions have been done on the specimen to date.

**Figure 3:**
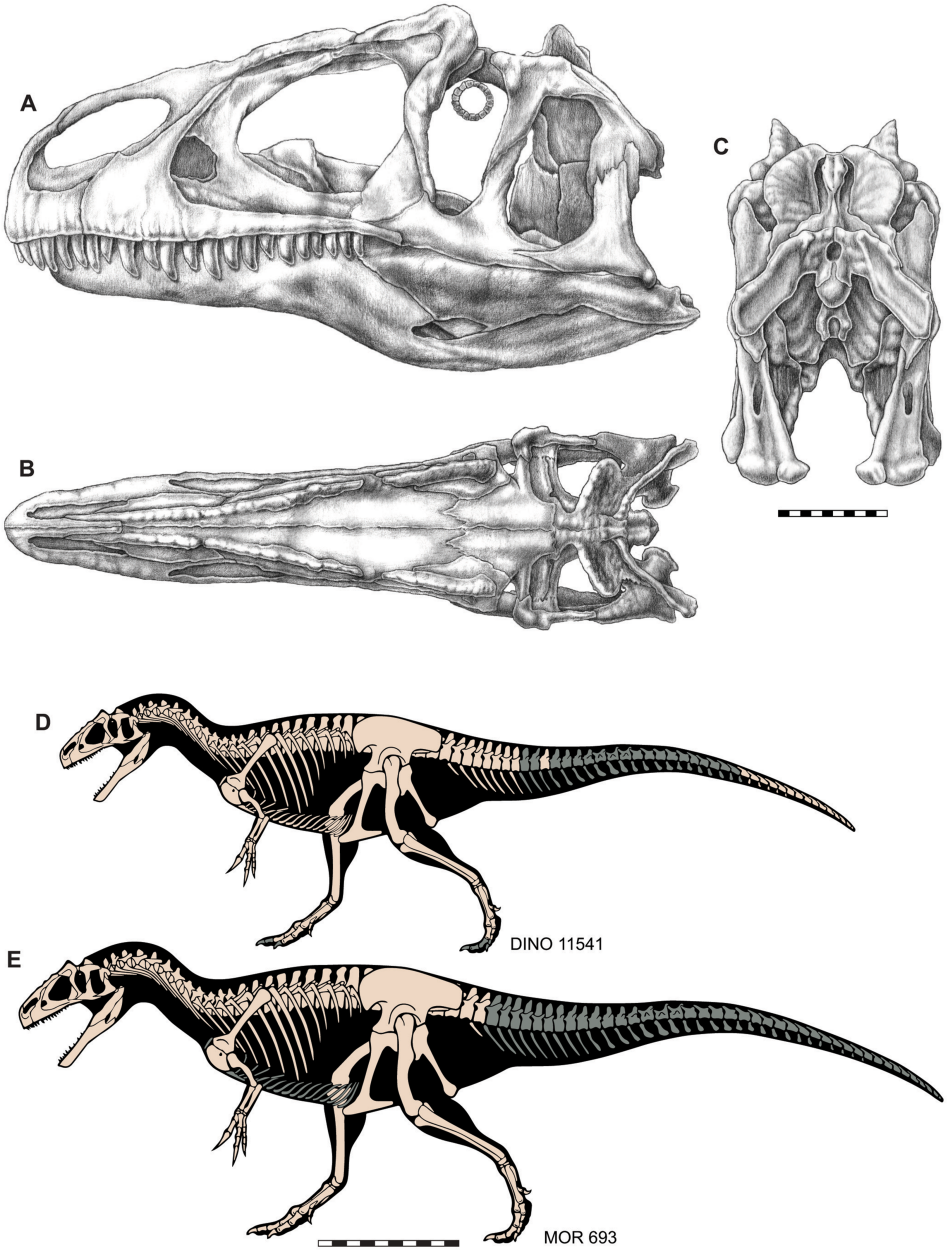
Skull and skeletal reconstructions of *Allosaurus jimmadseni*. Idealized skull of *Allosaurus jimmadseni* in lateral (A), dorsal (B) and posterior (C) views. Skeletal reconstructions of DINO 11541 (D) and MOR 693 (E). Missing elements in indicated in gray. A–C original artwork by Samantha Zimmerman; D and E are modified from artwork by Scott Hartman. Scale bar equals 10 cm for A–C; one m for D and E.

This paper describes both DINO 11541 and MOR 693 (Fig. 3) as a new species of *Allosaurus* and assigns other specimens from the Morrison Formation to the new taxon. The present description focuses on the head skeleton of the new taxon. We also differentiate this new species *Allosaurus jimmadseni* from the other two valid species of *Allosaurus*, *Allosaurus fragilis* and *Allosaurus europeaus*. Other previously named species of *Allosaurus* are invalid, including the recently named *Allosaurus lucasi* (Dalman, 2014), and are referable to either *Allosaurus fragilis* or are *Allosaurus* species indeterminate. These findings will be addressed in a subsequent review of species of *Allosaurus*, which is in preparation. The objective of this study is to provide a detailed description of the skull, mandible, dentition, atlas, and axis in a comparative context and to discuss the major cranial differences between the two species of *Allosaurus* in the Morrison Formation. Descripiton of the postcranial skeleton of *Allosaurus jimmadseni* will be the subject of a future paper.

## Materials and Methods

### Paleontological ethics statement

The specimens that are the focus of the descriptions in this paper (DINO 11541, and MOR 693) are reposited in the public repositories of Dinosaur National Monument and The Museum of the Rockies respectively. Both specimens were collected under permits obtained from the United States Department of the Interior and remain public property of the citizens of the United States. Other referred specimens come from lands administered by the BLM (USMN 544100 and SDSM 30510) and National Forest Service (BYU 4861, 5164, 5268, 5292, 5583, 11936, 13621, 16942, 17106, 17281, and other Dry Mesa Quarry allosaur materials) and were collected under research and excavation permits by other researchers. SMA 0005 was collected on private land and is reposited in the collection of the Sauriermuseum of Aathal in Switzerland. Locality information for each specimen is available from the specific repository institutions as per institutional policy. All necessary permits were obtained for the described study, which complied with all relevant regulations.

### Nomenclatural acts

The electronic version of this article in Portable Document Format (PDF) will represent a published work according to the ICZN, and hence the new names contained in the electronic version are effectively published under that Code from the electronic edition alone. This published work and the nomenclatural acts it contains have been registered in ZooBank, the online registration system for the ICZN. The ZooBank LSIDs (Life Science Identifiers) can be resolved and the associated information viewed through any standard web browser by appending the LSID to the prefix http://zoobank.org/. The LSID for this publication is: urn:lsid:zoobank.org:pub:DF37FD14-171C-4C02-8A5B-D2FCE929AABF. The online version of this work is archived and available from the following digital repositories: PeerJ, PubMed Central and CLOCKSS. The LSID for *Allosaurus jimmadseni* is: urn:lsid:zoobank.org:act:4D577308-64BC-4F87-A1F6-EE0467CF1A2F.

### Comparative material

In addition to the two specimens referenced above, we compared *Allosaurus* materials with a wide range of theropod taxa and accessed the ever-expanding literature focused specifically on non-coelurosaurian theropod dinosaurs. The authors have had the opportunity to study firsthand much of the theropod material collected globally over the past 20 years. Where published illustrations and descriptions were used to supplement data obtained through direct observation, appropriate references follow the specimen numbers. Where only the illustrations and descriptions of published works were used, only the references are cited. Theropods we compared *Allosaurus* material with include the basal theropods: *Tawa hallae* (GR 241, 155, 242, 243, 244), *Coelophysis bauri* (AMNH FR 7223, 7224, 7239, 7241, 7242; MNA V3315), *Dilophosaurus wetherilli* (UCMP 37302, 37303, 77270), *Ceratosaurus nasicornis* (USNM 4735; BYU 12893; BYU 13024; DINO 972; MWC 1, UMNH VP 5278), *Dubreuillosaurus valesdunensis* (MNHN 1998-13 (Allain, 2002; Allain, 2005)), *Piatnitzkysaurus floresi* (PVL 4073; MACN Pv CH895 (Bonaparte, 1986; Rauhut, 2004)), *Eustreptospondylus oxoniensis* (OUMNH J.3311 (Sadlier, et al., 2008)), *Monolophosaurus jiangi* (IVPP 84019 (Zhao & Currie, 1993; Brusatte, Benson & Xu, 2010; Zhao et al., 2010)), and the allosauroids *Sinraptor dongi* (IVPP 10600 (Currie & Zhao, 1993a)), *Sinraptor hepingensis* (Gao, 1992), *Acrocanthosaurus atokensis* (NCSM 14345 (Eddy & Clarke, 2011) and OMNH 10146 (Stovall & Langston, 1950; Harris, 1998; Currie & Carpenter, 2000)); and *Carcharodontosaurus saharicus* (SGM-Din 1). Additionally we have compared *Allosaurus* materials to the basal coelurosaurs *Tanycolagreus topwilsoni* (TPII 2000-09-29), *Coelurus fragilis* (YPM 1991–1995, 2010, 9162 (Carpenter et al., 2005)), *Sinosauropteryx prima* (NIGP 127586; NIGP 127587)*,Compsognathus longipes* (BSP AS I 563; MNHN CNJ79), *Juravenator starki* (Chiappe & Göhlich, 2010), *Scipionyx samniticus* (Dal Sasso & Maganuco, 2011) and *Ornitholestes hermanni* (AMNH 619); the basal tyrannosauroids *Proceratosaurus bradleyi* (Rauhut, Milner & Moore-Fay, 2009), *Kileskus aristotocus* (Averianov, Krasnolutskii & Ivantsov, 2010), *Guanlong wucaii* (IVPP V14531; V14532), *Iliosuchus incognitus* (Huene, 1932), *Juratyrant langhami* (OUMNH J.3311); *Stokesosaurus clevelandi* (UMNH VP 6051; 6052, 6383; 7434; 7818; 7821); *Dilong paradoxus* (IVPP V11579; V14242; V14243); *Sinotyrannus kazuoensis* (Ji, Ji & Zhang, 2009), and *Yutyrannus huali* (ZDCM 5000, 5001; ELDM V1001); and basal ornithomimids such as: *Aviatyrannis jurassica* (IPFUB Gui Th 1, 2, and 3), *Pelecanimimus polydon* (LH 7777), *Shenzhousaurus orientalis* (NGMC 97-4-002), and *Harpymimus okladnikovi* (IGM 100/29).

Exhaustive examination of *Allosaurus fragilis* for purposes of comparison included the proposed neotype USNM 4734 (Paul & Carpenter, 2010; Carrano, Loewen & Evers, 2018) and material from the CLDQ quarry including: UMNH VP 1251, 3113, 5316, 5326–5328, 5470, 5480, 6317, 6340, 6365, 6400, 6408, 6473, 6475, 6499, 6502, 7190, 7408, 7411, 7794, 7880, 7882, 7884–7885, 7889–7891, 7895, 7898, 7908, 7922, 7926–7930, 7932, 7934, 7937–7938, 7957, 7966, 8102, 8123, 8142, 8151, 8229, 8240–8241, 8355, 8397, 8484, 9103, 9147, 9149, 9162, 9168, 9180, 9191, 9201, 9212, 9323, 9327, 9366, 9376, 9401, 9470, 9473, 9480, 9500, 9502, 9505, 9514, 9709, 10360, 10386, 10779, 11031, 11463, 12231, 16584–16585, and other UMNH CLDQ material. Significant other specimens of *Allosaurus fragilis* excavated from CLDQ at other institutions were examined including specimens at: BYU (not to be confused with the Dry Mesa Quarry material); CEU; FMNH (P1505 and P25114); ROM (12868); and YPM. Other materials examined include AMNH 275, 287, 290, 324, 408,496, 600, 666, 680, 813, 851, 5750, 5753, 5767 (holotype, *Epanterias amplexus*), 6125, and 6128 from BCQ. *Allosaurus* material from the DNMCQ was examined including: CM 11844, and DINO 3984 and 2560 (previously catalogued as UUVP 6000). Articulated skulls MCZ 3897 R; YPM 1893; BYU 2028 (“Easter *Allosaurus*”) and BYU 571-8901 (“Hinkle *Allosaurus*”) were also examined.

### Terminology

We employ traditional, or “Romerian” anatomical and directional terms over veterinary alternatives (Romer, 1956; Wilson, 2006). For example, “anterior” and “posterior” are used as directional terms in lieu of the veterinary alternatives “rostral”, “cranial” and “caudal.” English equivalents of standard Latin terms are used, except for the musculature system, and directional terms follow Clark (1993). Terminology for pneumatic features is that of Witmer (1997a, 1997b).

## Results

### Systematic paleontology

Dinosauria Owen, 1842; sensu Padian & May, 1993
Saurischia Seeley, 1887; sensu Gauthier, 1986
Theropoda Marsh, 1881; sensu Gauthier, 1986
Tetanurae Gauthier, 1986
Allosaurioidea Currie and Zhao, 1994; sensu Carrano, Benson & Sampson, 2012
Allosauria Paul, 1988
Allosauridae Marsh, 1878; sensu Sereno, 2005
*Allosaurus* Marsh, 1877
*Allosaurus jimmadseni* Chure and Loewen sp. nov. (previously *nomen nudum* (Chure et al. 2006))
urn:lsid:zoobank.org:act:4D577308-64BC-4F87-A1F6-EE0467CF1A2F
Figures 1–16

**Etymology**—In honor of the late James H. Madsen, Jr and in recognition of his outstanding contributions to our knowledge of *Allosaurus* through his herculean efforts of protecting, excavating, preparing, and curating of many thousands of *Allosaurus* bones from the Cleveland-Lloyd Dinosaur and his masterful monograph (Madsen, 1976) of that collection.

**Figure 4:**
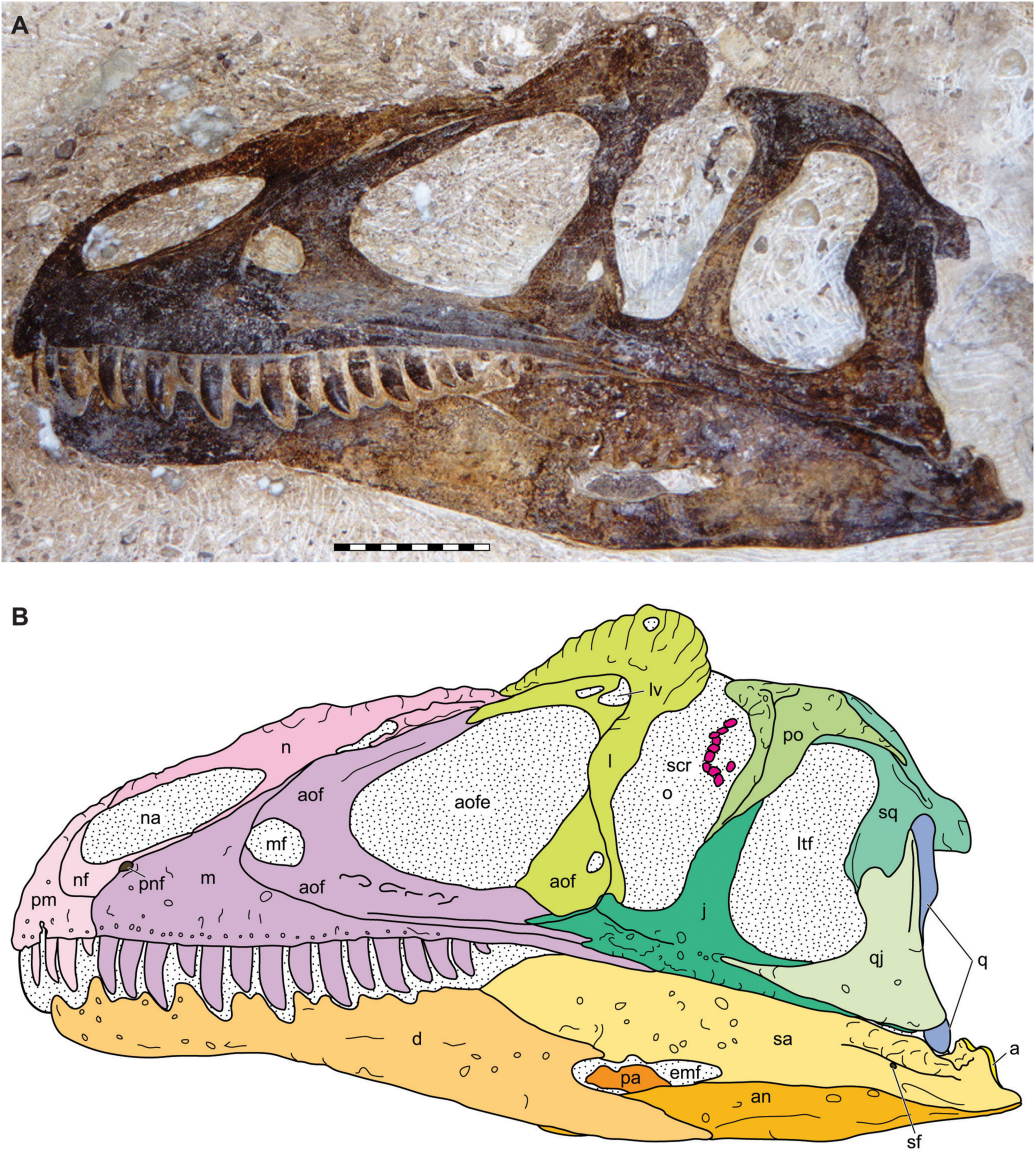
Lateral view of the skull of the holotype specimen of *Allosaurus jimmadseni* (DINO 11541). Photograph of skull (A) in left lateral view and (B) explanatory line drawing. Matrix shown as stippled in B. Photo by Dan Chure. Scale bar equals 10 cm. *Osteological abbreviations*: a, articular; an, angular; aof, antorbital fossa; aofe, antorbital fenestra; d, dentary; emf, external mandibular fenestra; j, jugal; l, lacrimal; lv, lacrimal vacuity; ltf, laterotemporal fenestra; m, maxilla; mf, maxillary fenestra; n, nasal; na, naris; nf, narial fossa (external naris); o, orbit; pa, prearticular; pm, premaxilla; pnf, perinarial fossa; po, postorbital; q, quadrate; qj, quadratojugal; sa, surangular; sf, surangular foramen; scr, sclerotic ring; sq, squamosal.

**Holotype**—DINO 11541 is a nearly complete and articulated skeleton, including: the left half of the skull with an occluded left mandible, an articulated vertebral column from cervical 2 through caudal 8, an isolated midcaudal vertebra, an articulated string of 16 distal caudal vertebrae from near the tip of the tail, cervical and dorsal ribs, a complete gastral basket, right and left scapulae, coracoids and articulated furcula, right and left humeri, left radius and ulna, four left carpals (two proximal (radiale and intermedium) and two distal), complete left tridactyl hand, complete pelvic girdle, right and left femora, tibiae, and fibulae, right astragalus and calcaneum, right and left distal tarsal III, left distal tarsal IV, right metatarsals I–IV, proximal half of left metatarsals II–IV, right pedal phalanges II and III-1 through 2, and right pedal phalanges IV-1 through IV-5 (Figs. 1, 3, 4, 6–13 and 16).

**Figure 5:**
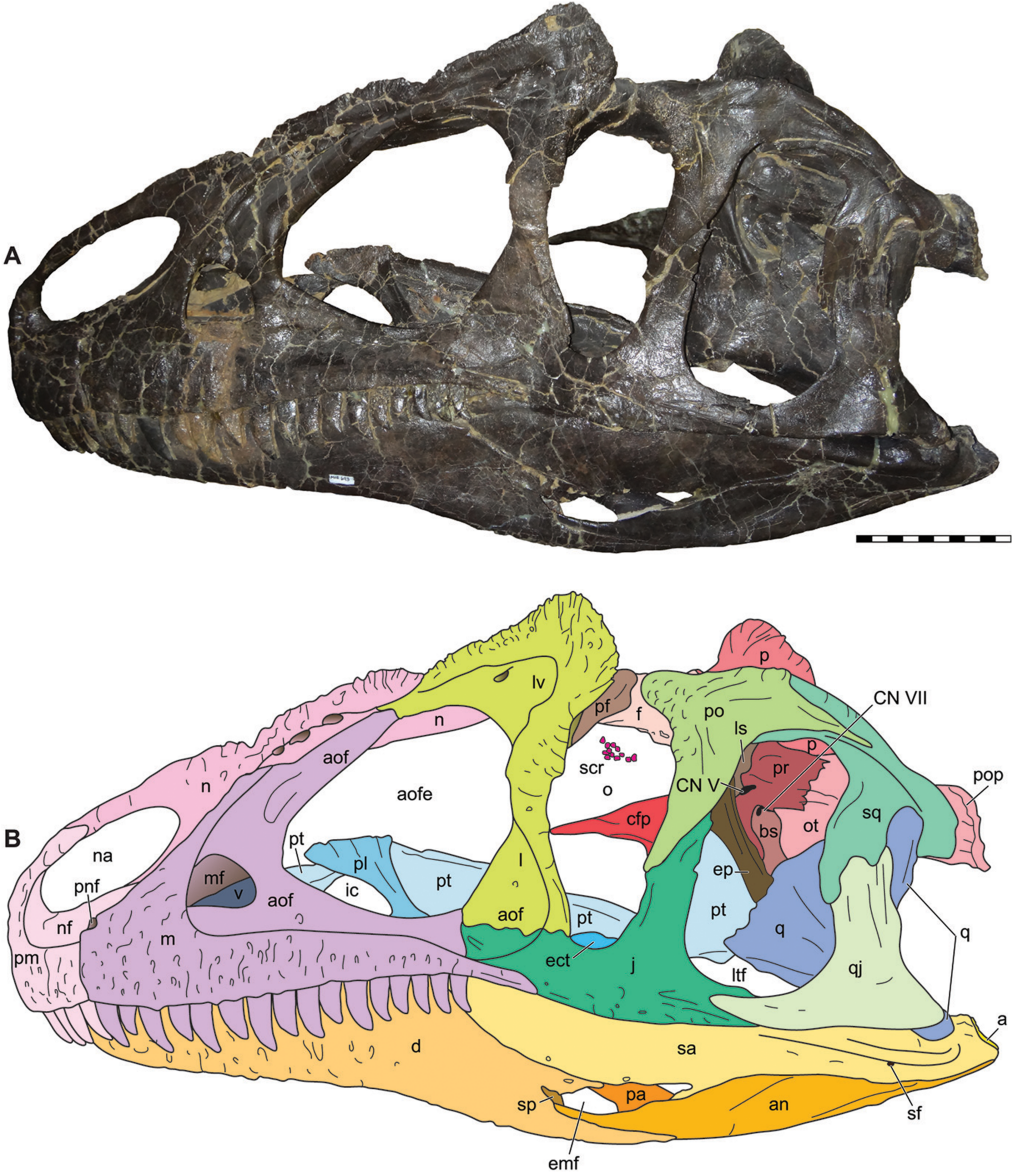
Lateral view of the skull of the referred specimen of *Allosaurus jimmadseni* (MOR 693). Photograph of the skull (A) in left lateral view and (B) explanatory line drawing. Photo by Mark Loewen. Scale bar equals 10 cm. *Osteological abbreviations*: a, articular; an, angular; aof, antorbital fossa; aofe, antorbital fenestra; bs, basisphenoid; cfp, cultriform process of the parasphenoid; CN V, trigeminal foramen; CN VII, facial nerve; d, dentary; ect, ectopterygoid; emf, external mandibular fenestra; ep, epipterygoid; f, frontal; j, jugal; l, lacrimal; lv, lacrimal vacuity; ltf, laterotemporal fenestra; m, maxilla; mf, maxillary fenestra; n, nasal; na, naris; nf, narial fossa (external naris); o, orbit; ot, otoccipital; p, parietal; pa, prearticular; pf, prefrontal; pl, palatine; pm, premaxilla; pnf, perinarial fossa; po, postorbital; pop, paroccipital process of the otocippital; pr, prootic; pt, pterygoid; q, quadrate; qj, quadratojugal; sa, surangular; scr, sclerotic ring; sf, surangular foramen; sq, squamosal.

**Figure 6:**
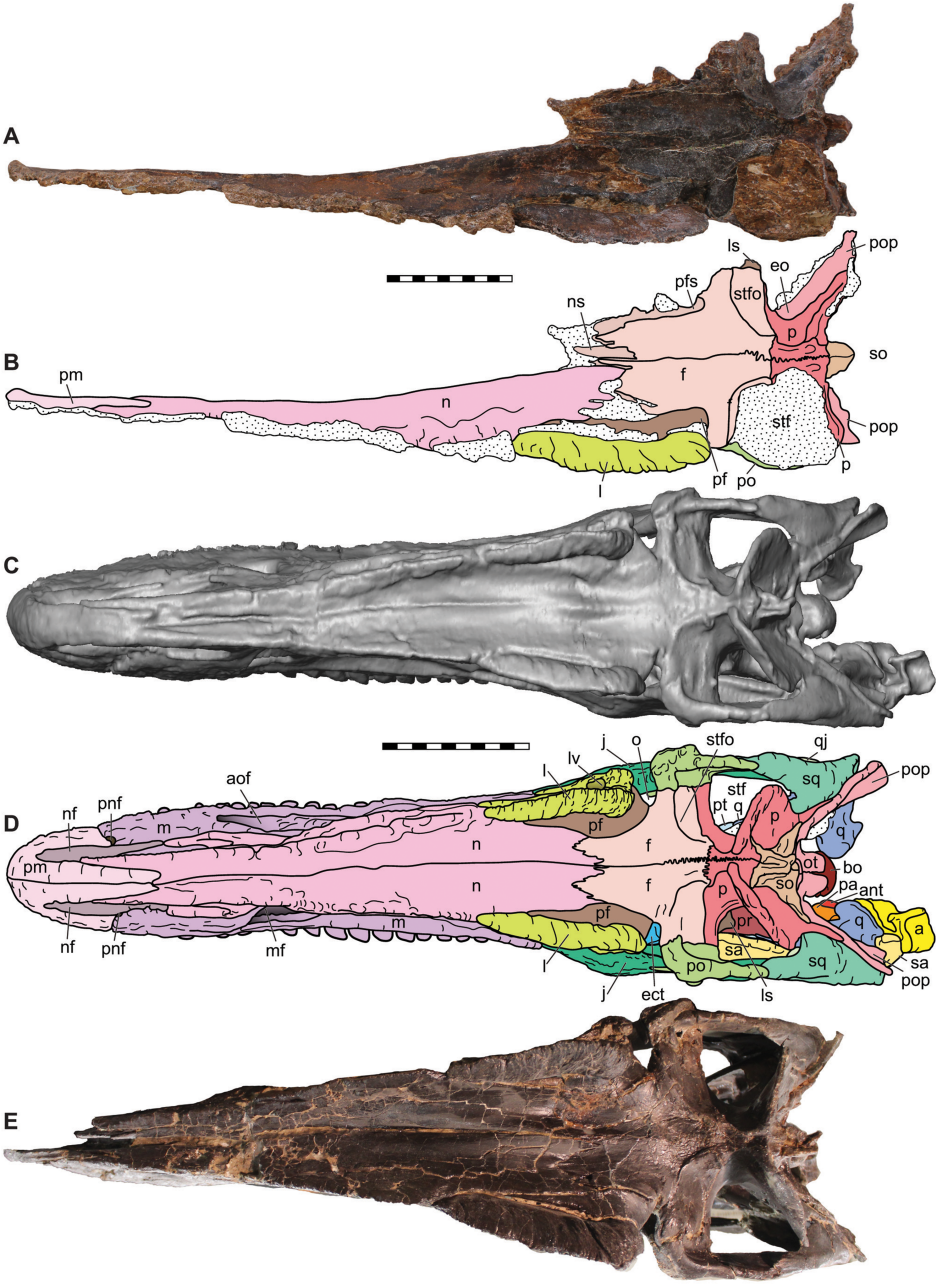
Dorsal view of the skulls of *Allosaurus jimmadseni* (DINO 11541 and MOR 693). Photograph of the skull (A) and explanatory line drawing (B) of the holotype of *Allosaurus jimmadseni* (DINO 11541). A CT surface scan of a cast of the skull of MOR 693 (C) and explanatory line drawing (D). Also included is a photograph of MOR 693 (E) that includes lense parallax when compared to the CT surface scan (C). Matrix shown as stippled. CT surface scan courtesy Eric Snively and Larry Witmore. Photos by Mark Loewen. Scale bars equal 10 cm. *Osteological abbreviations*: a, articular; ant, antarticular; aof, antorbital fossa; bs, basisphenoid; ect, ectopterygoid; ep, epipterygoid; f, frontal; j, jugal; l, lacrimal; ls, laterosphenoid; lv, lacrimal vacuity; m, maxilla; mf, maxillary fenestra; n, nasal; nf, narial fossa (external naris); o, orbit; oc, occipital condyle; ot, otoccipital; p, parietal; pa, prearticular; pf, prefrontal; pm, premaxilla; pnf, perinarial fossa; po, postorbital; pop, paroccipital process of the otocippital; pr, prootic; pt, pterygoid; q, quadrate; qj, quadratojugal; sa, surangular; sq, squamosal; so, supraoccipital; stf, supratemporal fenestra; stfo, suprtemporal fossa.

**Referred material**—Referred specimens include: MOR 693 (“Big Al”), a nearly complete associated skeleton, including an articulated skull (Figs. 2, 3, 5, 6, 8, 10–12 and 14–16); SMA 0005 (“Big Al II”), a nearly complete associated skeleton, including disarticulated skull and skin impressions on the base of the tail; USMN 544100; SDSM 30510, a juvenile partial skeleton and other disarticulated adult material from the Little Houston Quarry, Wyoming; all allosaurid material from the Dry Mesa Quarry, CO curated at BYU including: BYU 4861, 5164, 5268, 5292, 5583, 11936, 13621, 16942, 17106, 17281; and unpublished material from the Meilyn Quarry reposited as casts at the NHMU (UMNH VPC 481).

**Holotype locality**—DINO 11541 was recovered from locality DNM 116, east of the enclosed Carnegie Quarry in the Utah part of Dinosaur National Monument. Exact locality data are on file at Dinosaur National Monument.

**Holotype horizon**—DINO 11541 was recovered from the Salt Wash Member of the Upper Jurassic (Kimmeridgian) Morrison Formation. All referred specimens occur in the stratigraphically equivalent lower part of the Morrison Formation in Wyoming.

**Referred localities**—Localities include: The Big Al Quarry (BAQ), Big Horn County, Wyoming; Dry Mesa Quarry (DMQ), Colorado; DNM-116 at Dinosaur National Monument (DNMSW), Salt Wash Member, Uinta County, Utah; Dana Quarry (DQ), Washaki County, Wyoming; Howe Ranch Quarry (HQ), Howe Stephens Quarry (HSQ), Big Horn County, Wyoming; and Little Houston Quarry (LHQ), Crook County, Wyoming.

**Regional horizon**—*Allosaurus jimmadseni* was found in the Salt Wash Member of the Morrison Formation in Utah and lower part of the Brushy Basin Member of the Morrison Formation in Wyoming and South Dakota. *Allosaurus jimmadseni* occurs below the “clay change” of Turner & Peterson (1999), except for at DMQ, which occurs only two m above the “clay change”.

**Age**— *Allosaurus jimmadseni* was found in the Salt Wash Member of the Morrison Formation and its lateral equivalents. The Tidwell Member near the base of the Morrison (below the Salt Wash Member) produced a date of 154.82 ± 0.58 Ma (RAIN-1325-4+4 of Kowallis et al. (1998)) and a date of 150.18 ± 0.51 Ma (LCM-1 of Kowallis et al. (1998)) was recovered at the base of the overlying Brushy Basin Member. These two dates constrain the the Salt Wash Member between them. These single-crystal, laser-fusion ^40^Ar/^39^Ar ages on sanidine crystals were recalibrated (Irmis, Nesbitt & Sues, 2013) to 157.32 ± 0.61 Ma (RAIN-1325-4+4 of Kowallis et al. (1998)) and a date of 152.77 ± 0.3 Ma following the Monte Carlo method of Renne et al. (2010). This places it in the Kimmeridgian Age of the Late Jurassic Epoch (Walker et al., 2012).

**Diagnosis**—*Allosaurus jimmadseni* is distinguished from other basal tetanurans by the following unique combination of characters: (1) in lateral view, a row of neurovascular foramina pierce the medioventral wall of the maxillary antorbital fossa; (2) straight posteroventral jugal ramus of maxilla where it articulates with jugal; (3) laterodorsal margin of nasal “pinched” into low crest continuous from premaxilla to lacrimal; (4) posterior portion of dorsal surface of nasal cup-shaped, producing a median peak in region of nasofrontal contact; (5) relatively taller lacrimal horns than in *Allosaurus fragilis*; (6) jugal with relatively straight ventral margin and straight-to-slightly-curved outline in dorsal view; a well-developed distinct antarticular, and (7) axial intercentrum is rotated dorsally and has a flared rim in lateral view.

**Figure 7:**
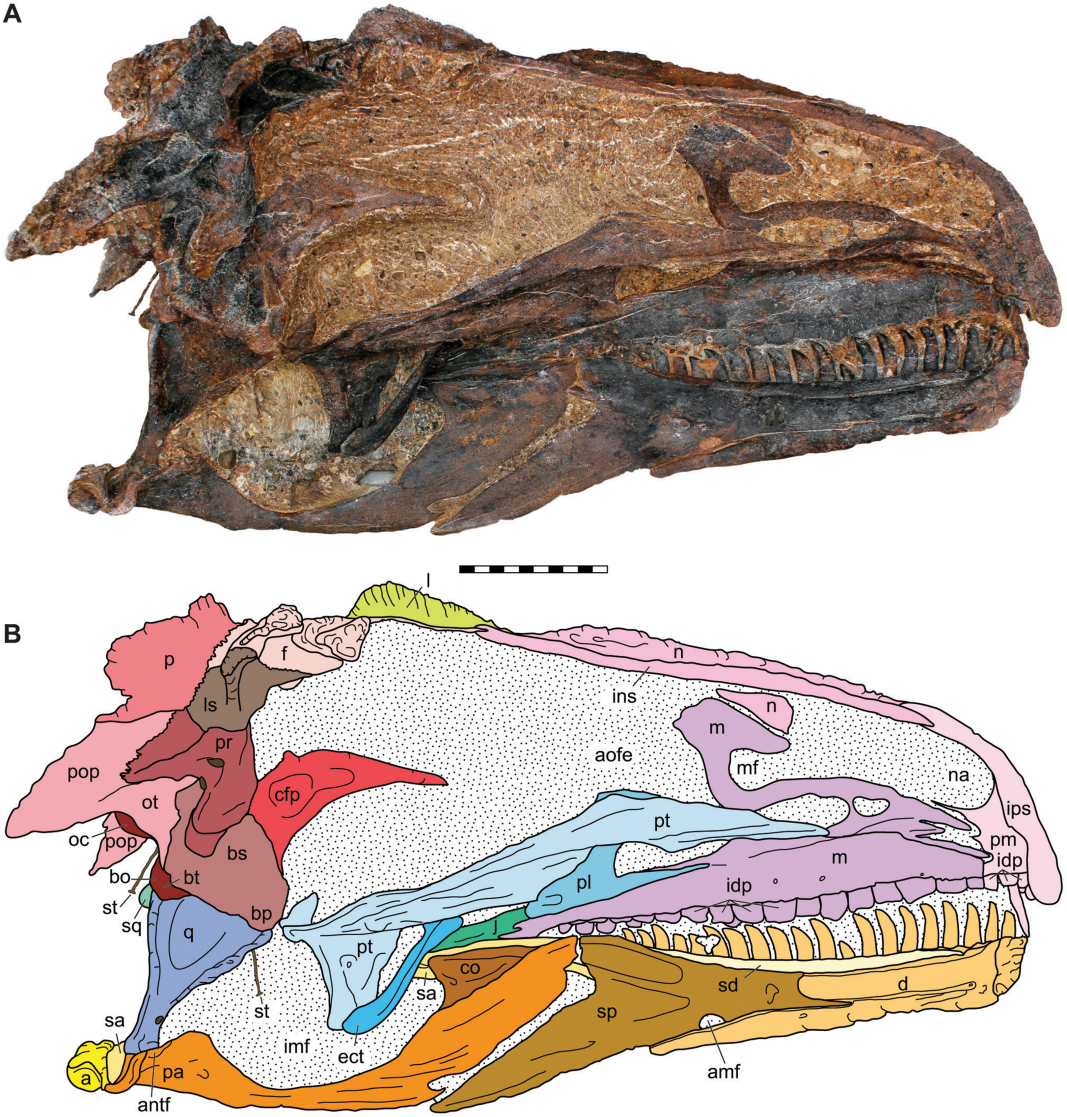
Medial view of the skull of the holotype specimen of *Allosaurus jimmadseni* (DINO 11541). Photograph of skull (A) in dorsal view and (B) explanatory line drawing. Matrix shown as stippled in (B). Photo by Serjoscha Evers. Scale bar equals 10 cm. *Osteological abbreviations*: a, articular; amf, anterior mylohyoid foramen; an, angular; antf, antarticular facet; aofe, antorbital fenestra; bo, basioccipital; bp, basipterygoid; bs, basisphenoid; bt, basal tubera; cfp, cultriform process of the parasphenoid; co, coronoid; d, dentary; ect, ectopterygoid; f, frontal; idp, intradental plates; imf, internal mandibular fenestra; ins, internarial suture; ips, intrapremaxillary suture; j, jugal; l, lacrimal; ls, laterosphenoid; m, maxilla; mf, maxillary fenestra; n, nasal; na, naris; oc, occipital condyle; ot, otoccipital; p, parietal; pa, prearticular; pl, palatine; pm, premaxilla; po, postorbital; pop, paroccipital process of the otocippital; pr, prootic; pt, pterygoid; q, quadrate; qj, quadratojugal; sa, surangular; sd, supradentary; sp, splenial; sq, squamosal; st, stapes.

**Figure 8:**
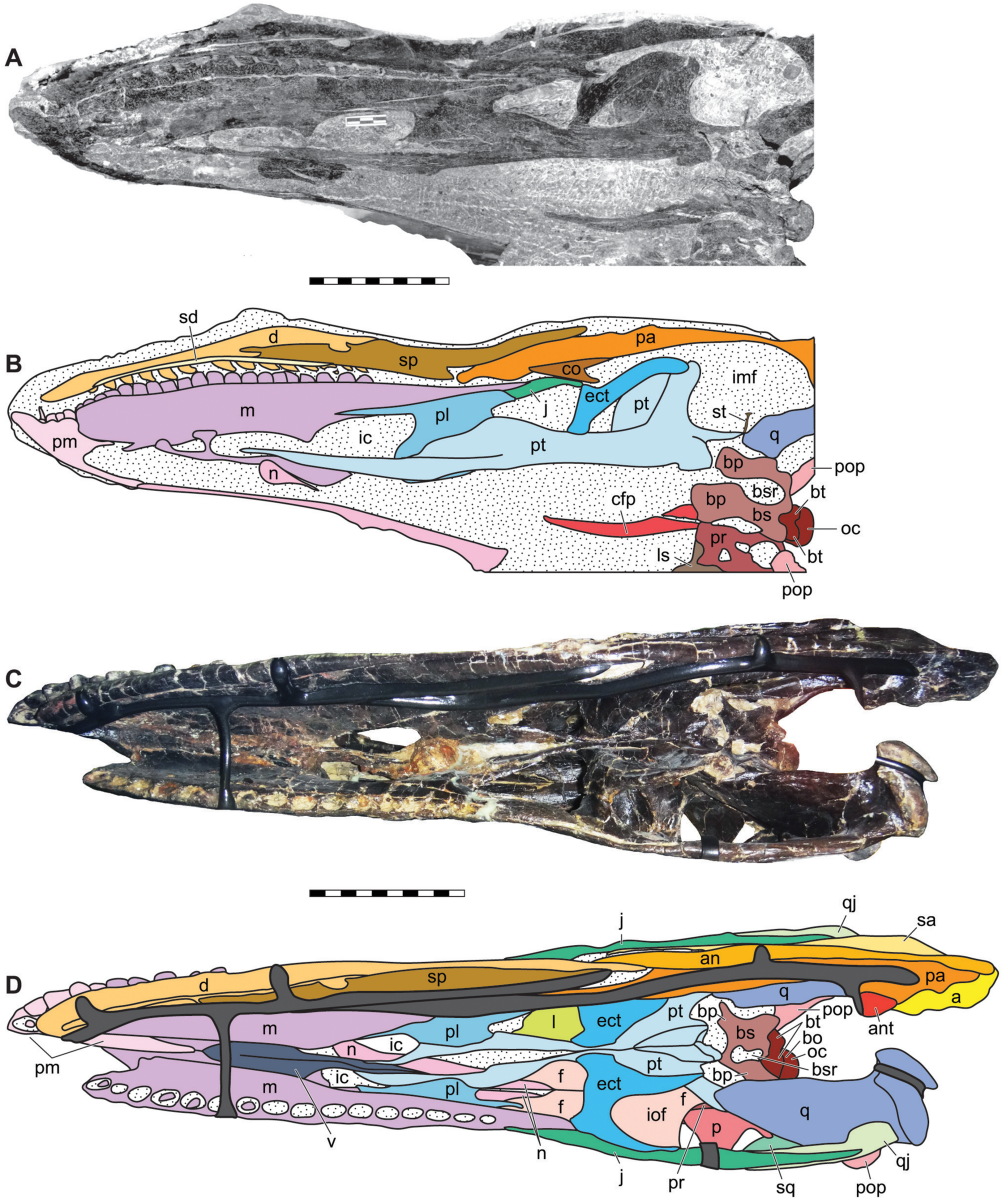
Ventral view of the skulls of *Allosaurus jimmadseni* (DINO 11541 and MOR 693). Ventral oblique photograph of the skull of DINO 11541 (A) and interpretative line drawing (B). Ventral photograph of the skull of MOR 693 (C) and explanatory line drawing (D). Matrix shown as stippled, dark grey represents skull mounting armature. Photos by Dan Chure and Serjoscha Evers. Scale bars equals 10 cm. *Osteological abbreviations*: a, articular; an, angular; ant, antarticular; bo, basioccipital; bp, basipterygoid; bs, basisphenoid; bsr, basisphenoid recess; bt, basal tubera; cfp, cultriform process of the parasphenoid; d, dentary; ect, ectopterygoid; f, frontal; ic, internal choanae; imf, internal mandibular fenestra; iof, infraorbital fenestrae; j, jugal; l, lacrimal; ls, laterosphenoid; m, maxilla; n, nasal; oc, occipital condyle; p, parietal; pa, prearticular; pl, palatine; pm, premaxilla; pop, paroccipital process of the otocippital; pr, prootic; pt, pterygoid; q, quadrate; qj, quadratojugal; sa, surangular; sd, supradentary; sq, squamosal; sp, splenial; sq, squamosal; st, stapes; v, vomer.

**Figure 9:**
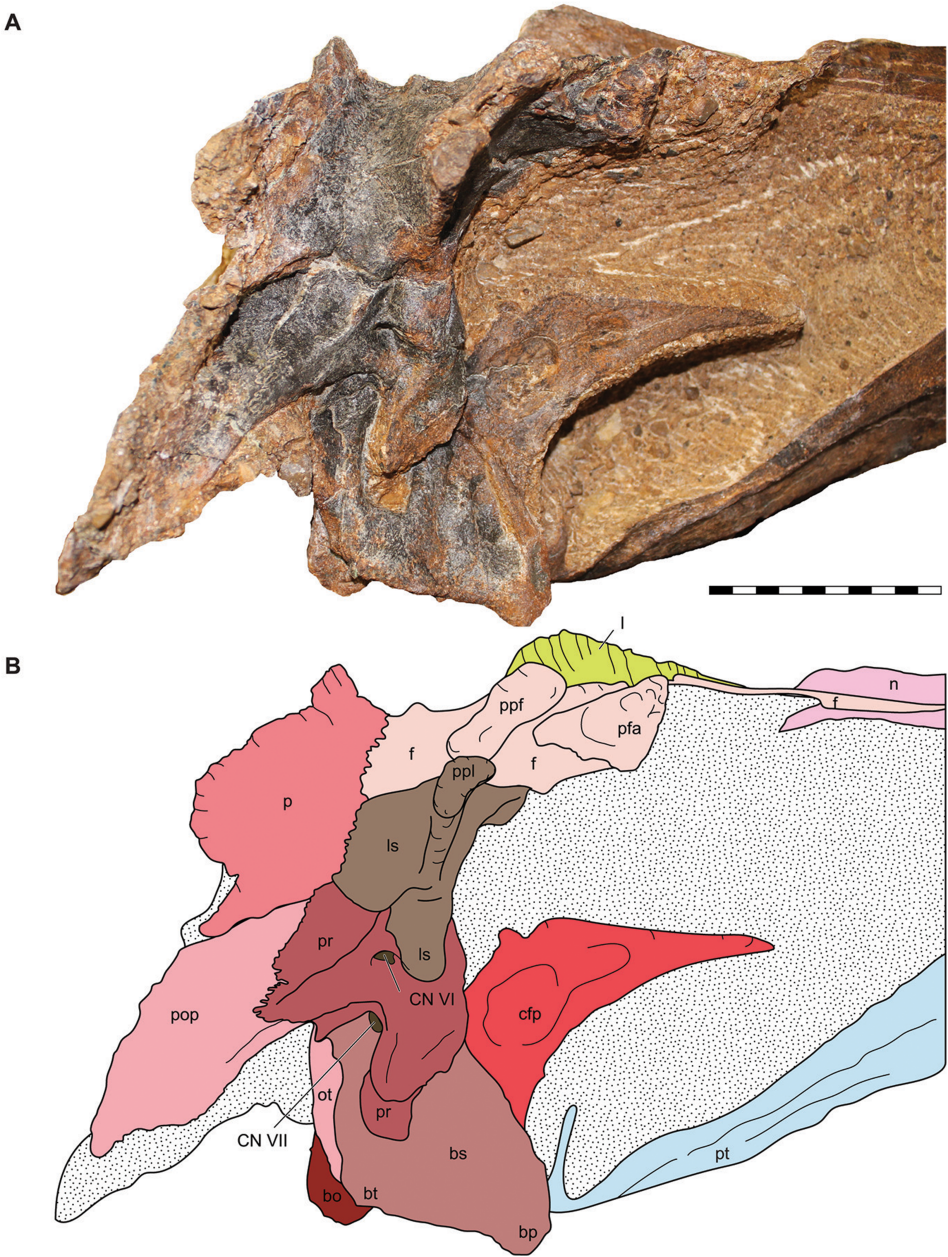
Lateral view of the braincase of the holotype specimen of *Allosaurus jimmadseni* (DINO 11541). Photograph of the braincase of DINO 11541 (A) by Serjoscha Evers and explanatory line drawing (B). Matrix shown as stippled. Scale bars equals 10 cm. *Osteological abbreviations*: bo, basioccipital; bp, basipterygoid process; bt, basal tubera; bs, basisphenoid; cfp, cultriform process of the parasphenoid; CN V, trigeminal foramen; CN VII, facial nerve; l, lacrimal; ls, laterosphenoid; oc, occipital condyle; ot, otoccipital; p, parietal; pfa, prefontal articulation; pop, paroccipital process of the otocippital; ppf, postorbital process of the frontal; ppl, postorbital process of the laterosphenoid; pr, prootic; pt, pterygoid.

**Figure 10:**
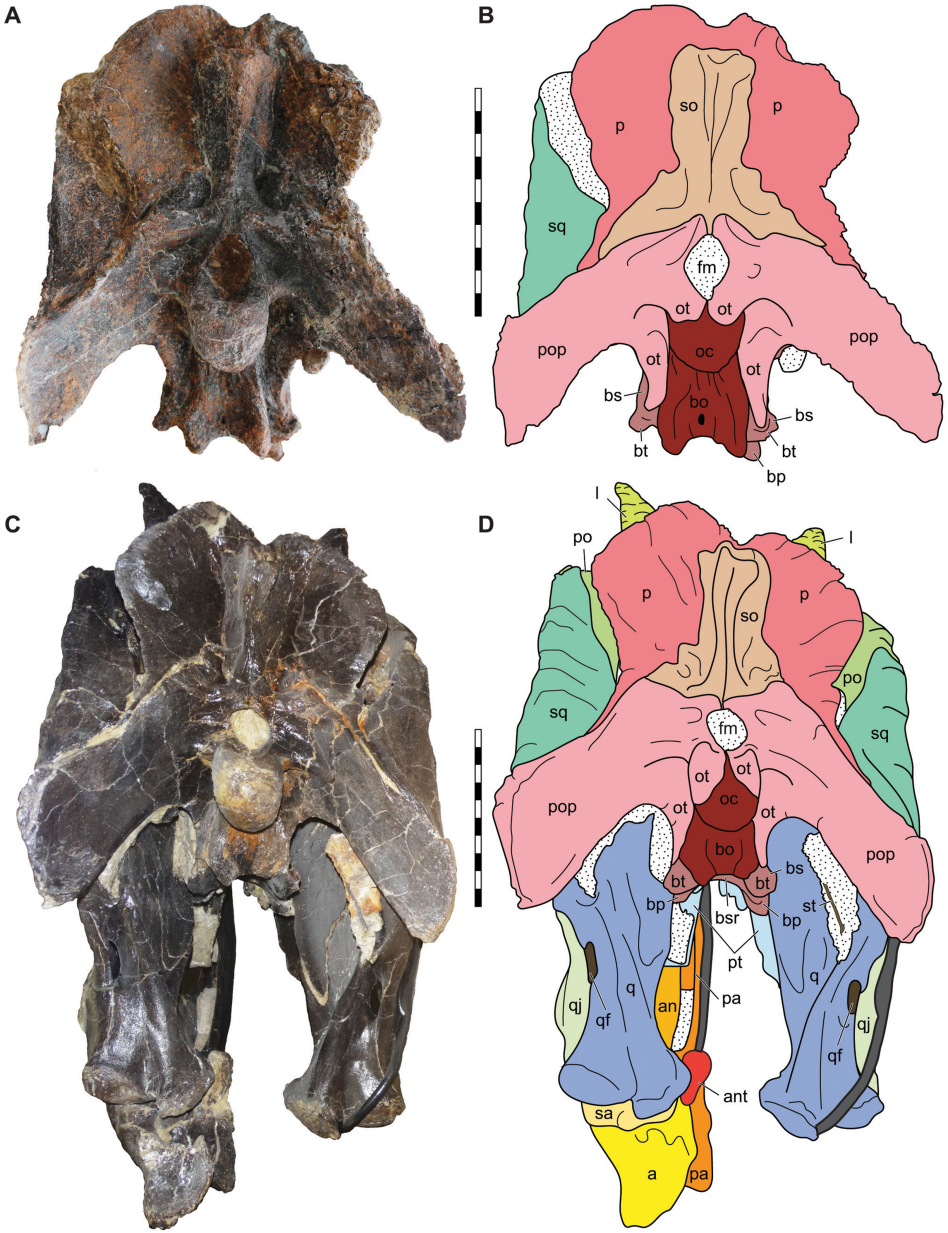
Posterior view of the skulls of *Allosaurus jimmadseni* (DINO 11541 and MOR 693). Photograph of the skull of DINO 11541 (A) and MOR 693 (C) and explanatory line drawings (B) and (D). Matrix shown as stippled; dark grey represents skull mounting armature. Scale bars equals 10 cm. Photos by Mark Loewen and Serjoscha Evers. *Osteological abbreviations*: a, articular; an, angular; ant, antarticular; bo, basioccipital; bp, basipterygoid; bt, basal tubera; bs, basisphenoid; bsr, basisphenoid recess; fm, foramen magnum; l, lacrimal; oc, occipital condyle; ot, otoccipital; p, parietal; pa, prearticular; po, postorbital; pop, paroccipital process of the otocippital; pt, pterygoid; q, quadrate; qf, quadrate foramen; qj, quadratojugal; sa, surangular; sq, squamosal; so, supraoccipital; st, stapes.

**Figure 11:**
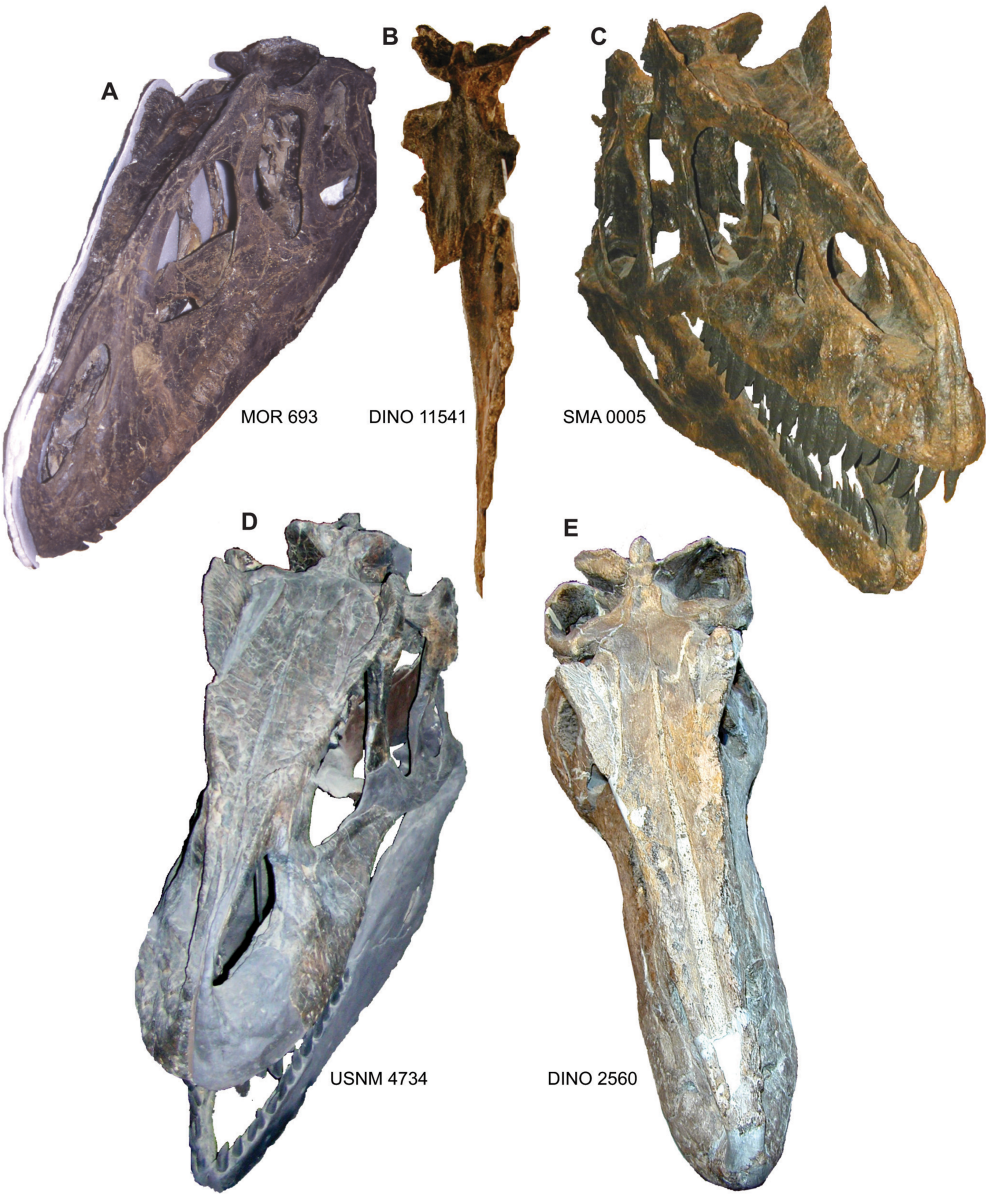
Comparison of the nasals of *Allosaurus*. Nasals of *Allosaurus jimmadseni* including: MOR 693 (A), DINO 11541 (B), and SMA 0005 (C) in oblique and dorsal views illustrating the pinched nature of the nasal crest along the lateral margin of the nasal. Jugals of *Allosaurus fragilis* from USNM 4734 (D) and DINO 2560 (E). Photos by Mark Loewen.

**Figure 12:**
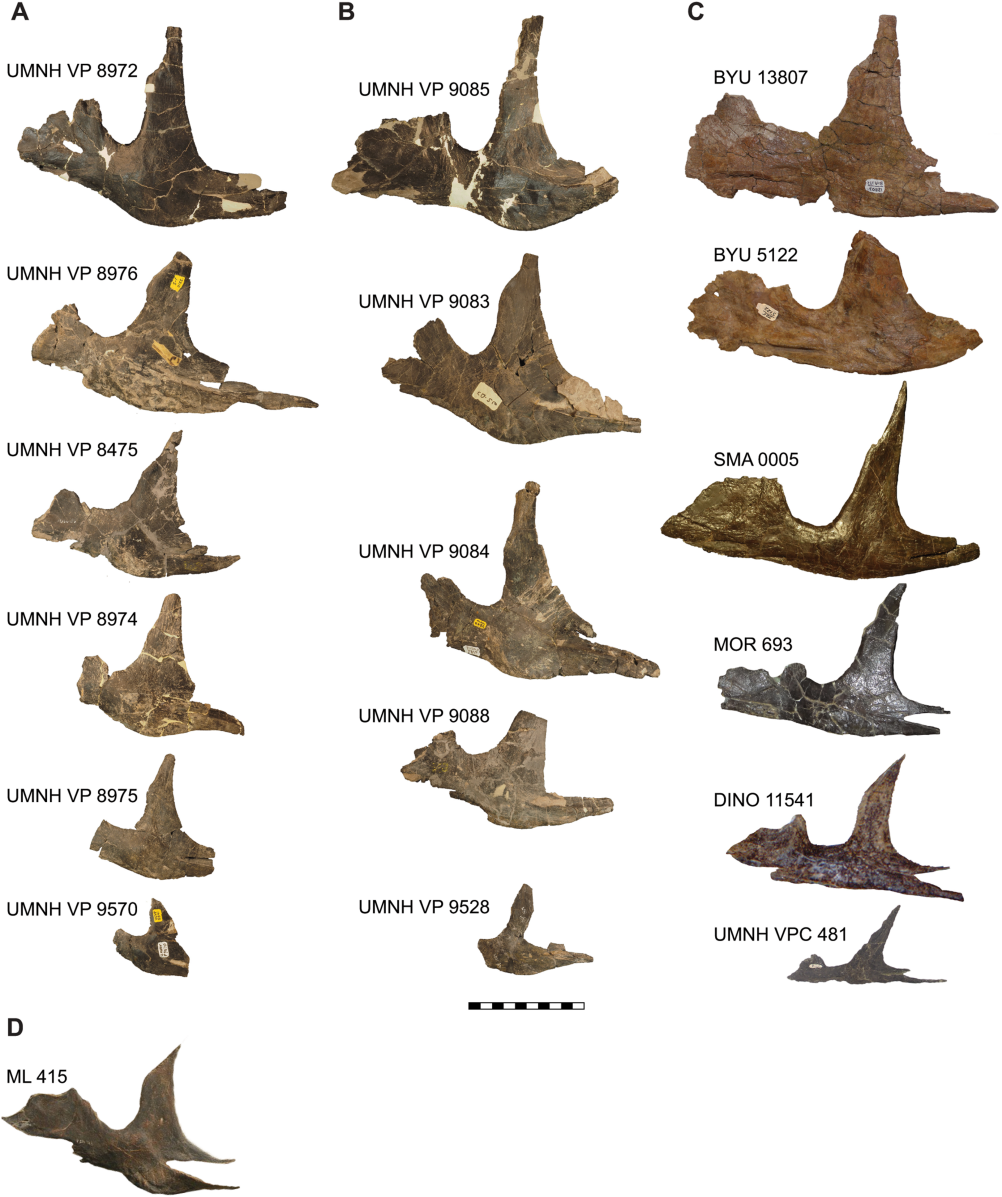
Comparison of the jugals of *Allosaurus*. (A) Left jugals representing an ontogenetic series of *Allosaurus fragilis* from the Cleveland-Lloyd Dinosaur Quarry in left lateral view, note the highly sigmoidal ventral margin. (B) Right jugals representing an ontogenetic series of *Allosaurus fragilis* from the Cleveland-Lloyd Dinosaur Quarry that have been photoreversed for comparison. (C) Jugals of *Allosaurus jimmadseni* from top to bottom in order of descending size: BYU 13807 (photoreversed), BYU 5122, SMA 0005, MOR 693, DINO 11541, and UMNH VP C481 (photoreversed). (D) The left jugal of *Allosaurus europeaus* ML 415. The jugals of *Allosaurus jimmadseni* have a much flatter ventral margin. Photos by Mark Loewen. Scale bar equals 10 cm.

**Figure 13:**
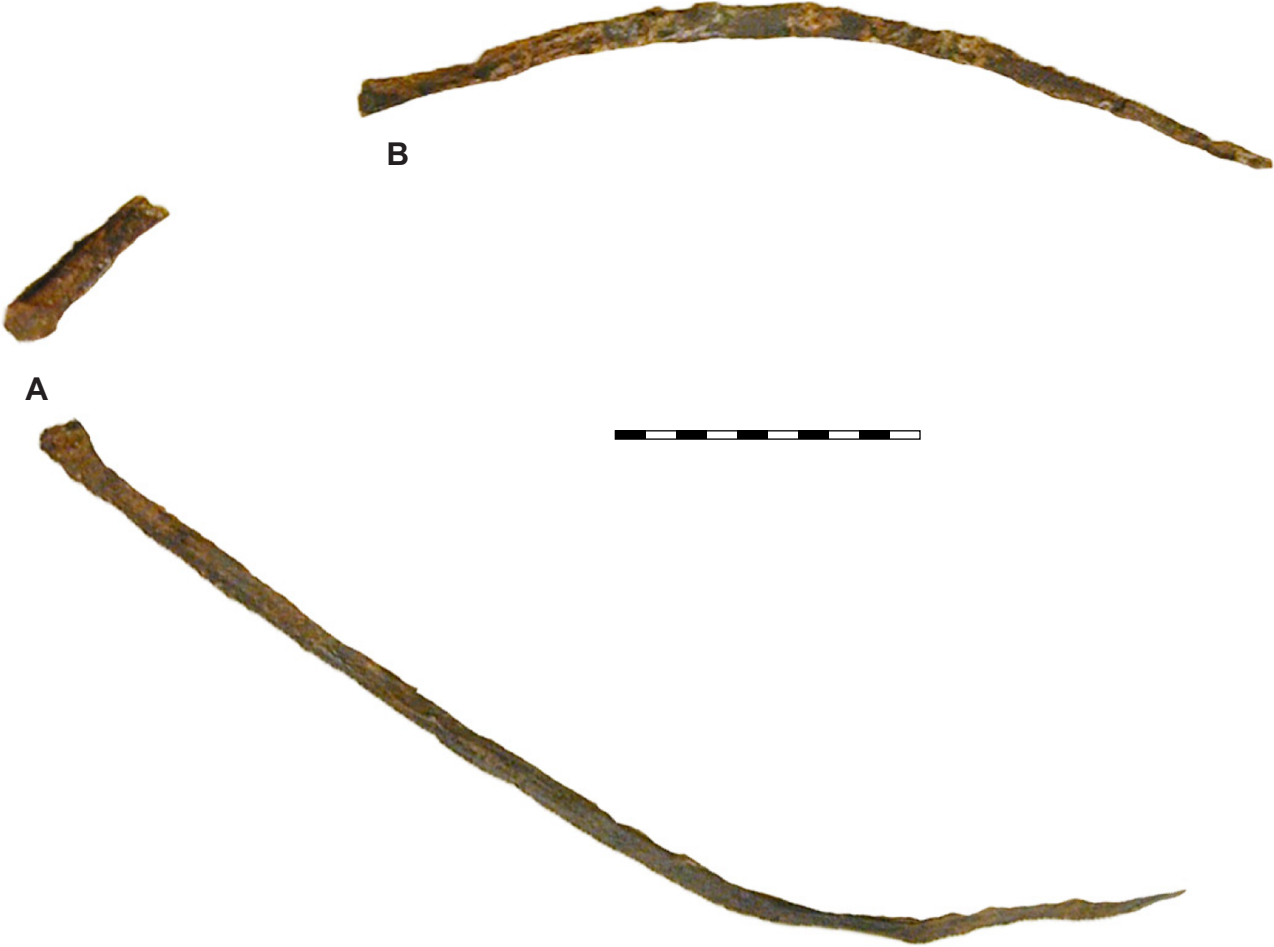
Hyoids of *Allosaurus jimmadseni*. (A) Left? hyoid and anterior end of right hyoid as preserved together in articulation on skull of DINO 11541. (B) Displaced posterior portion of right? hyoid. Photos by Mark Loewen. Scale bar equals 10 cm.

**Figure 14:**
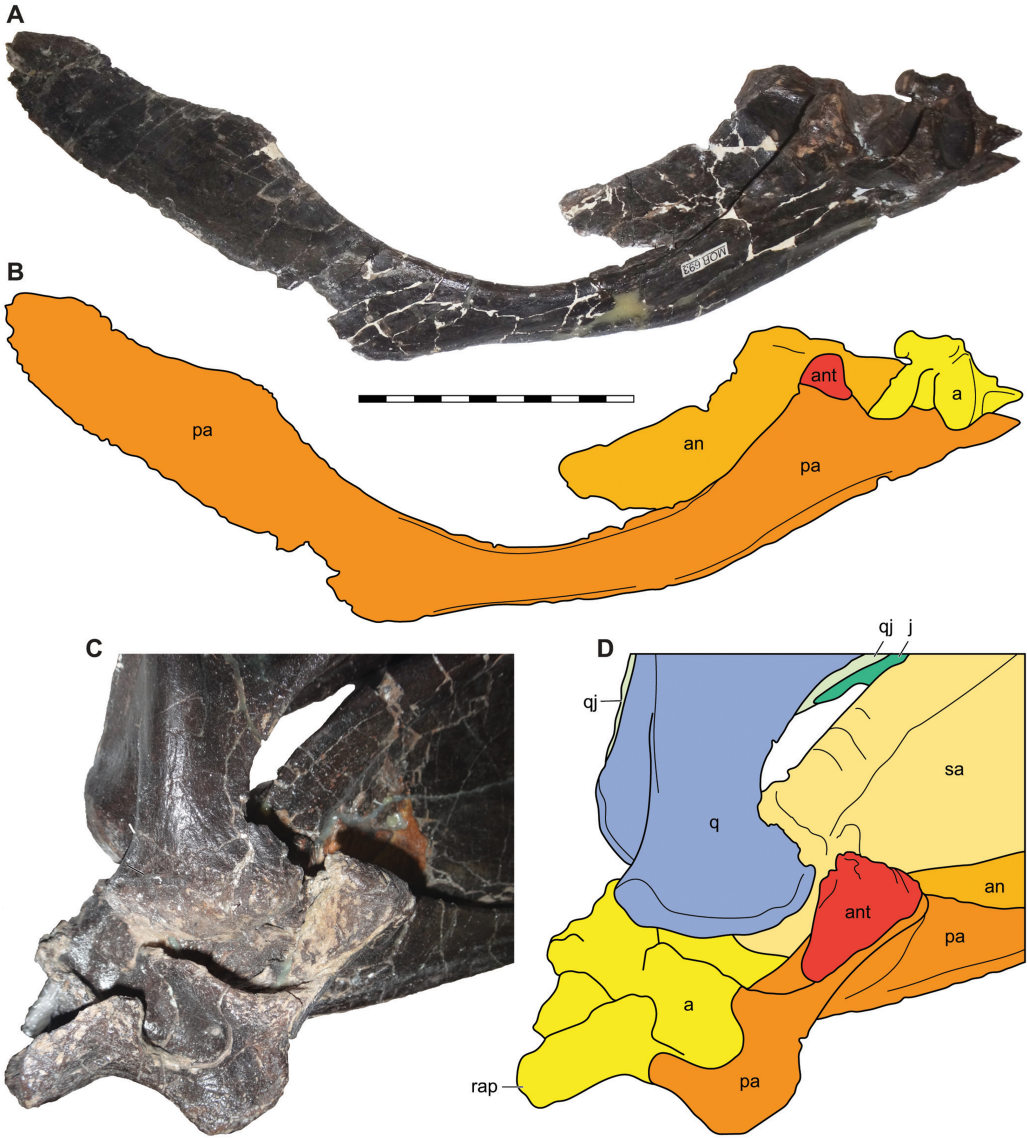
Antarticular of *Allosaurus jimmadseni*. Medial view of disarticulated posterior right mandible of MOR 693 (A) and explanatory drawing (B) showing the relative position of the articulated antarticular. Oblique posteromedial view of the articulated left mandible of MOR 693 (C) and (D) explanatory drawing illustrating the position of the antarticular on the prearticular. Photos by Mark Loewen. Scale bar equals 10 cm. *Osteological abbreviations*: a, articular; an, angular; ant, antarticular; j, jugal; pa, prearticular; q, quadrate; qj, quadratojugal; rap, retroarticular process.

**Figure 15:**
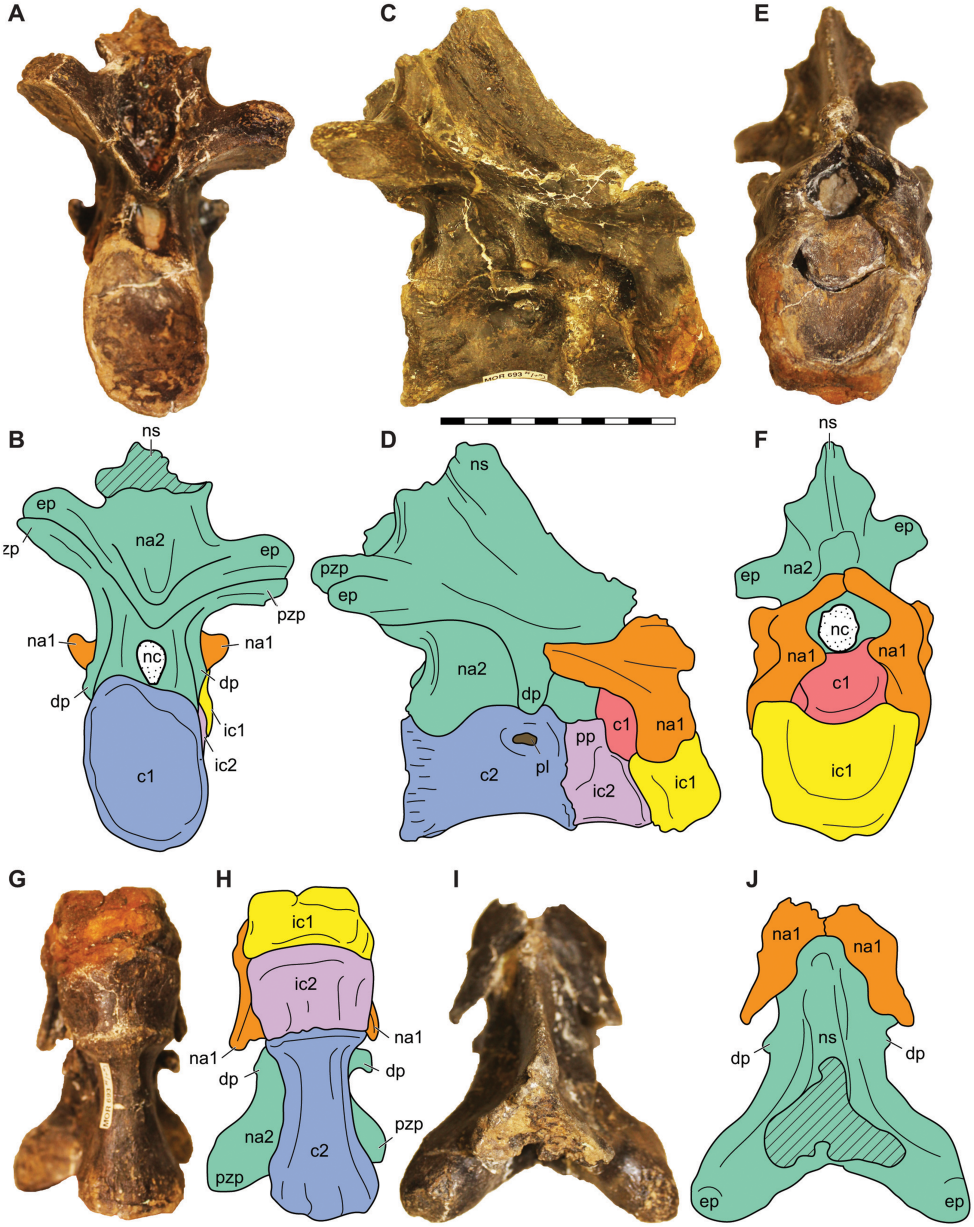
Atlantoaxial complex of *Allosaurus jimmadseni*. Atlantoaxial complex of *Allosaurus jimmadseni* in posterior view (A) with explanatory drawing (B), in right lateral view (C) with explanatory drawing (D); in anterior view (E), with explanatory drawing (F); in ventral view (G) with explanatory drawing (H); and in dorsal view (I) with explanatory drawing (J). Photos by Sorjosha Evers. Scale bar equals 10 cm. *Osteological abbreviations*: c1, atlantal centrum or odontoid; c2, axial centrum; dp, diapophysis; ep, epipophysis; ic1, atlantal intercentrum; na1, atlantal neural arch; nat, axial neural arch; nc, neural canal; ns, neural spine; pzp, postzygapophysis.

**Figure 16:**
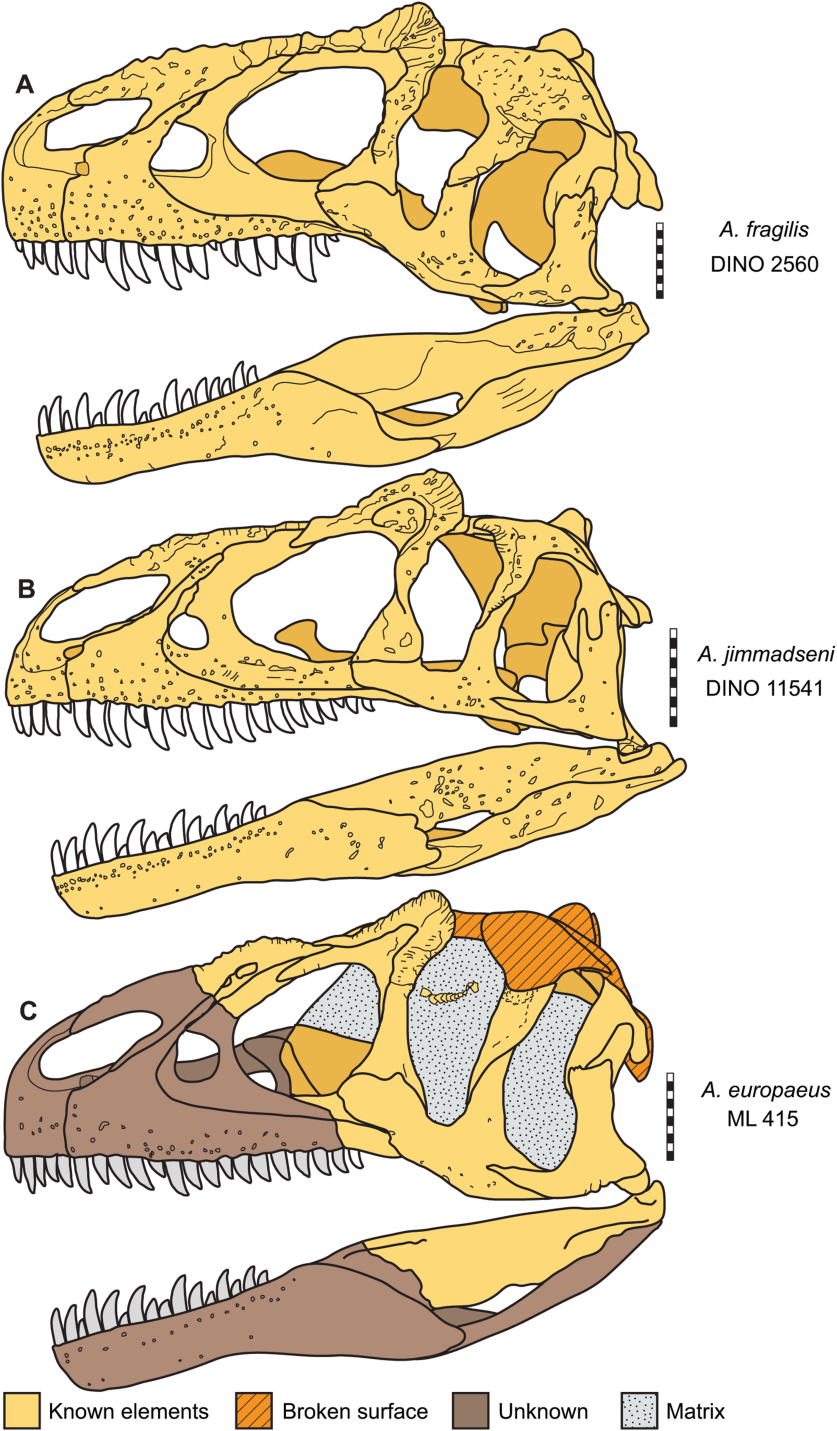
Skulls of *Allosaurus* in left lateral view. (A) *Allosaurus fragilis* (DINO 2560). (B) *Allosaurus jimmadseni* (DINO 11541). (C) *Allosaurus europeaus* (ML 415). Scale bars equal 10 cm.

## Description and Comparisons of *Allosaurus jimmadseni*

### General description of the skull and lower jaw

The skull of DINO 11541 was found approximately two m downstream from the axis, resting on its left side (Fig. 1). The skull is separated along the midline suture of the premaxillae and nasals and lacks the right side of the skull and the palate. The braincase, frontals and parietals are preserved slightly rotated out of their natural position. The prepared areas of the skull include: the dorsal and left side of the skull, the occipital, the right side of the braincase, and the lateral and medial surfaces of the left hemimandible.

The skull of MOR 693 was found in articulation on its left side, with the right premaxilla and mandible slightly disarticulated and displaced. The skull was still attached to the first six cervical vertebrae and included sclerotic ring segments on the left side and the hyoid bones in place (Figs. 1 and 4).

As is typical in theropods and many predatory archosaurs, the premaxillary and maxillary teeth project lateral to the dentary, obscuring the dorsal margin of the dentary and its dentition when the jaws were occluded. The lower jaw is shorter than the skull, resulting in an overbite as present in almost all ornithodires. Distinct external and internal mandibular fenestrae are present as in most theropods.

### Major cranial fenestrae, foramina, and fossae

**External narial fossae**—The external narial fossa (Figs. 3–6) in *Allosaurus jimmadseni* is restricted to the body of the premaxilla and to the narial process of the premaxilla. It is expressed as a smooth, semicircular area around the anteroventral margin of the external nares. This fossa is present in many theropods and is usually delineated by a marked groove, although it appears to be absent in *Neovenator* (Hutt, Martill & Barker, 1996; Brusatte, Benson & Hutt, 2008). This fossa is less well delineated in *Allosaurus jimmadseni* than in *Allosaurus fragilis*, but still well defined. The ventral extent of the narial fossa extends 1/3 of the length of the body of the premaxilla, in contrast to a more dorsal position in *Monolophosaurus* and *Acrocanthosaurus*.

**External nares**—The external nares (Figs. 3–6) are large and elliptical, oriented roughly at 20° from horizontal. The external naris is made up of the narial process of the premaxilla anteriorly and a quarter of the dorsal surface of the external naris, and by the body of the premaxilla for half of its ventral extent. The remainder of the external naris is defined by the premaxillary process and subnarial process of the nasal. The maxilla is excluded from the margin of the external nares by the subnarial processes of the premaxilla and nasal. *Allosaurus fragilis* is reported to have the maxilla participating in the margin of the external nares (Madsen, 1976), but the specimen illustrated is, as stated in the caption, a composite skull. Witmer (1997a, 1997b) modified this drawing and illustrated the vestibular bulla of the maxilla as forming the maxillary margin of the external nares. Osborn (1903) showed a larger part of the maxilla forming part of the margin in AMNH 600 and 666. Examination of the left maxilla of AMNH 600 shows grooves for the subnarial processes of the premaxilla and nasal overlap where they meet, with the premaxillary groove running medial to the nasal groove. Thus, the maxilla is excluded from the margin of the external nares in AMNH 600 (*contra* Osborn (1903)). Other well-preserved skulls of *Allosaurus fragilis* (BYU 9466 and DINO 2560) also show that condition.

In basal theropods the maxilla is primitively excluded from the narial margin (*Herrerasaurus ischigualastensis*, *Dilophosaurus wetherilli*), although it is included in the narial margin in *Coelophysis bauri* and *Coelophysis rhodesiensis* (Colbert, 1989; 1990). However, that condition is variable among allosauroids. The maxilla is excluded from the narial border in *Sinraptor dongi* (Currie & Zhao, 1993a, 1993b), *Yangchuanosaurus shangyuensis*, and *Yangchuanosaurus shangyouensis* (Dong, Zhou & Zhang, 1983) and *Monolophosaurus jiangi* and *Neovenator salerii* (Hutt, Martill & Barker, 1996; Brusatte, Benson & Hutt, 2008). The condition is unknown in *Cryolophosaurus ellioti*, *Giganotosaurus carolinii, Piatnitzkysaurus floresi*, and *Yangchuanosaurus* (Smith et al., 2007; Coria & Salgado, 1995; Dong, 1987; Dong, Zhou & Zhang, 1983). Because the premaxilla is displaced, the condition is difficult to determine in *Sinraptor hepingensis*, but it appears that the maxilla did contribute to the narial margin (Gao, 1992).

**Perinarial fossae**—As in most theropods, the perinarial fossa (Figs. 4–6) is located on the premaxilla-maxilla suture close to the ventral margin of the external nares, unlike *Sinraptor dongi*, where the perinarial fossa is primarily on the lateral surface of the subnarial process of the nasal (Currie & Zhao, 1993a), and to *Neovenator* and *Eocharcharia* (Sereno & Brusatte, 2008) in which the fossa is nearly completely surrounded by the maxilla (Brusatte, Benson & Hutt, 2008). The position of the perinarial fossa in *Sinraptor hepingensis* is difficult to determine with certainty, but it appears to be similar to that in *Sinraptor dongi*.

**Antorbital fossae**—The antorbital fossa (Figs. 3–6) is large in *Allosaurus jimmadseni* and its margin is marked by a low, weak ridge. This ridge is expressed on the lateral surface of the maxilla from the anterior extent of the jugal suture at the posterior end of the maxilla and arcs gently anteriorly to the level of the maxillary fenestra and then rises vertically until it meets the nasal. The antorbital fossa extends onto the nasal, across the lacrimal, circumnavigating the lacrimal vacuity, and down the ventral process of the lacrimal. The only jugal contribution to the antorbital fossa is a two mm wide strip of the anterormost surface of the jugal. The path of the ridge is diagnostic for *Allosaurus* among Morrison theropods (Britt, 1991). The maxilla inside the antorbital fossa forming the wall of the antorbital cavity is generally smooth. However, in DINO 11541 ventral to the internal antorbital fenestra there is a series of five small depressions, each with a foramen in its roof; there is one of these depressions present on the left side of MOR 693. These neurovascular foramina in DINO 11541 and MOR 693 differ in size and depth from the single openings lacking larger depressions that are rarely present in specimens of *Allosaurus fragilis* (e.g., UMNH VP 5427) or other allosauroids.

**Antorbital fenestrae**—The internal antorbital fenestra (Figs. 3–7) is large, and sub-triangular. A maxillary fenestra is present, with the anterior surface of the maxillary fenestra tucked inside the antorbital fossa. Neither the promaxillary strut nor the promaxillary fenestra is visible in lateral view, although they can be seen in medial view. As in *Allosaurus fragilis* (Witmer, 1997a, 1997b) the promaxillary fenestra and strut are in a recess on the anterior surface of the maxillary fenestra and medial to the lateral surface of the maxilla.

In *Monolophosaurus* (Zhao & Currie, 1993) the position and size of the maxillary fenestra is like that in *Allosaurus* (although Witmer (1997a, 1997b) suggests that this might be the promaxillary fenestra). It also appears to be similar in *Giganotosaurus* (Coria & Salgado, 1995), although a detailed description has yet to be provided. The condition in *Sinraptor* and *Yangchuanosaurus* has been variously interpreted. *Sinraptor dongi* (Currie & Zhao, 1993a) has a large maxillary fenestra, entirely visible in lateral view, with a small accessory pneumatic foramen posterior to it. The maxilla of *Yangchuanosaurus shangyuensis* is not significantly different from that of *Sinraptor dongi* (Dong, Zhou & Zhang, 1983). However, Witmer (1997a, 1997b) has interpreted the large fenestra in sinraptorids (*Yangchuanosaurus* and *Sinraptor*) as the promaxillary fenestra and the smaller posterior “accessory fenestra” to be a reduced maxillary fenestra. It appears that in *Sinraptor hepingensis* (Gao, 1992) the reduced maxillary fenestra is completely lost and the large fenestra is present in the promaxillary fenestra (Witmer, 1997a, 1997b). In both *Sinraptor* and *Yangchuanosaurus* the promaxillary fenestra is wholly or nearly completely visible in lateral view. In *Acrocanthosaurus* the maxillary fenestra is larger than the promaxillary fenestra, but both are visible in lateral view. *Carcharodontosaurus* is reported as having a “maxillary fenestra” that is barely visible in lateral view (Sereno et al., 1996). The maxillary fenestra is not anteriorly located in other allosauroids and in light of Witmer’s interpretation of the condition in sinraptorids (especially *Sinraptor hepingensis*), it is reasonable to interpret the fenestra in *Carcharodontosaurus* as a promaxillary fenestra and the maxillary fenestra as being lost. In *Neovenator* there is a large maxillary fenestra but the margins are not complete (Hutt, Martill & Barker, 1996; Brusatte, Benson & Hutt, 2008). *Piatnitzkysaurus* is strikingly different from other allosauroids in having a medial wall to the maxillary sinuses (Bonaparte, 1986), as in *Marshosaurus bicentesimus*, *Afrovenator abakensis*, *Deinonychus antirrhopus*, and *Ornitholestes hermanni* (Sereno et al., 1994; Ostrom, 1969; Witmer, 1997a, 1997b).

**Orbital fenestrae**—The orbit is formed by the lacrimal anteriorly, the jugal ventrally and by the jugal and postorbital posteriorly (Figs. 3–6). The lacrimal and postorbital form most of the dorsal portion of the orbit, except for the mid dorsal region in which there is a medial notch that is walled by the frontal and prefrontal. There is a distinct suboccular process on the preorbital ramus of the lacrimal below the position of the eye. This projection probably marks, as in birds, the anterior insertion of the *Ligamentum suborbitale* (Baumel & Witmer, 1993), and therefore delineates the anteroventral margin of that part of the orbit occupied by the eye (Chure, 2000b). In most specimens of *Allosaurus fragilis* the posterior margin of the preorbital ramus of the lacrimal is concave. There is no posterior process of the lacrimal, and there is no posterior process in any other species of *Allosaurus*.

The orbit in *Allosaurus jimmadseni* forms a dorsoventrally oriented oval, which is similar to that of *Allosaurus fragilis*. The primitive orbit shape in theropods is a large, circular orbit. In allosauroids, the basic shape is dorsoventrally elongated. However, this elongated shape also occurs in more basal tetanurans (*Torvosaurus tanneri*, *Afrovenator abakensis*, *Baryonyx walkeri*) as well as the coelurosaurian tyrannosaurids and ceratosaurian abelisaurids. There is some variation in orbital shape in allosauroids (Chure, 2000a), here we recognize three basic shapes in the group:

1. Oval and unrestricted: *Allosaurus fragilis*, *Allosaurus jimmadseni*, *Ceratosaurus*, *Saurophaganax*, *Sinraptor dongi*, and *Yangchuanosaurus shangyuensis (*also present in the spinosauroid *Baryonyx walkeri* and the megalosaurids *Afrovenator abakensis* and *Torvosaurus tanneri)*.
2. Circular orbit with markedly tapering ventral portion: *Monolophosaurus*, and possibly *Cryolophosaurus*,
3. Anterior projection of the postorbital restricts the orbit at approximately mid-height: *Acrocanthosaurus atokensis*, *Carcharodontosaurus saharicus*, *Giganotosaurus carolinii*, and, to a lesser degree, *Sinraptor hepingensis* (also present in the abelisaurids *Abelisaurus comahuenis*, *Carnotaurus sastrei*, *Majungasaurus* and the tyrannosaurids *Bistahieversor*, *Teratophoneus*, *Lythronax*, *Tyrannosaurus rex*, and *Tarbosaurus bataar*).

The sclerotic ring is preserved in the dorsal portion of the orbit in both DINO 11541 and MOR 693 (Figs. 4 and 5). In DINO 11541 only part of the sclerotic ring is visible. Sclerotic elements continue underneath the descending ramus of the postorbital and are obscured by matrix that still remains in the orbit. While preservation of the ring is not good (the ring is distorted and the pattern of overlap in the plates can’t be determined), there are at least eight plates present which probably represent one quadrant of the sclerotic ring. The sclerotic ring is clearly small and the eye would occupy only the dorsalmost part of the orbit (Chure, 2000b). In MOR 693 the sclerotic ring was collapsed on itself and subsequently prepared out of the skull. The position of the sclerotic ring in Fig. 5 is based on a photo of the specimen during preparation. Both MOR 693 and DINO 11541 indicate that the eye was subequal in size to the maxillary fenestra in *Allosaurus jimmadseni*.

**Supratemporal fossae**—The supratemporal fossae (Fig. 6) is the surface expression of the origin of *M. pseudotemporalis*. The supratemporal fossa is expressed as a ridge on the frontal, postorbital, squamosal and on the parietal. In *Allosaurus jimmadseni* the supratemporal fossa are separated medially by a small flat skull table. The skull table between the supratemporal fossae is narrower than in *Acrocanthosaurus* (Eddy & Clarke, 2011) but wider and more elongate than those of *Sinraptor* (Currie & Zhao, 1993a), which also has a notchlike configuration constrained to a narrow crescentic notch across the length of the parietals. The supratemporal fossa in *Allosaurus* and *Acrocanthosaurus* (Eddy & Clarke, 2011) is suboval rather than the posteriorly elongated teardrop shape in *Sinraptor* (Currie & Zhao, 1993a).

**Supratemporal fenestrae**—The supratemporal fenestra (Fig. 6) is the dorsal opening of the skull posterior to the orbits and is bordered by the frontal anteriorly, the parietal medially and posteriorly, and by the postorbital and squamosal laterally. The dorsal rim of this fenestra is formed by a rim of the supratemporal fossa.

The supratemporal fenestrae are typical for theropod dinosaurs with minor differences from those of other allosauroids. The overall shape of the supratemporal fenestrae in *Allosaurus* and *Acrocanthosaurus* (Eddy & Clarke, 2011) is suboval compared to the posteriorly elongated teardrop shape in *Sinraptor* (Currie & Zhao, 1993a).

**Laterotemporal fenestrae**—The laterotemporal fenestra (Figs. 3–5) is bordered by the jugal and postorbital anteriorly, the postorbital and squamosal dorsally, the squamosal, and quadratojugal anteriorly and the jugal and quadratojugal ventrally. It is suboval in shape, with a slight medial constriction formed by the ventral process of the squamosal lapping onto the anterior surface of the dorsal process of the quadratojugal. Two thirds of the anterior margin is formed by the jugal ventrally and one third by the postorbial dorsally. The dorsal surface is almost entirely formed by the squamosal in DINO 11541 and subequally by the postorbital and squamosal in MOR 693. The posterior margin is subequally formed by the squamosal and quadratojugal and the ventral border is subequally formed by the jugal and quadratojugal. The laterotemporal fenestra is subequal in size to the orbital fenestra and is identical to the laterotemporal fenestra in *Allosaurus fragilis*.

The laterotemporal fenestra differs in size from *Sinraptor dongi* (Currie & Zhao, 1993a) in which the laterotemporal fenestra is nearly twice the size of the orbit, and the inflection of the ventral squamosal process is much more dorsally situated. The fenestra differs from *Acrocanthosaurus atokensis* (Eddy & Clarke, 2011) in both the jugal contribution to the anterior border which makes up 85%, and in its dorsal position of the ventral squamosal process inflection. The posterior inflection is much slighter than in tyrannosauroids.

**Internal choanae**—The internal choanae (Fig. 8) are positioned at the level of the anterior end of the antorbital fenestra and expressed in the roof of the mouth. They are formed by the maxilla anterolaterally and by the vomer, palatine, and vomerine process of the pterygoid medially. The posterior portion of the internal choanae is formed by the palatines. They do not differ significantly form the conformation present in other allosauroids in which they are preserved.

**Infraorbital fenestrae**—The infraorbital fenestrae (Fig. 8) are positioned below the orbital fenestrae on the ventral surface of the skull. They are formed by the palatines anteriorly, the pterygoids medially, the ectopterygoids posteriorly, and by the pterygoid and maxilla laterally. They are similar to those of other allosauroids in which they are preserved.

**Foramen magnum**—The foramen magnum (Figs. 9 and 10) is formed by the exoccipitals and supraoccipital dorsally, the exocippitals laterally and by the basioccipital ventrally. The supraoccipital contribute less than 2% to the margin of the foramen magnum dorsally. This is similar to the condition in *Allosaurus fragilis* in which the supraoccipital contributes to the dorsal margin of the foramen magnum. The foramen magnum is 50% the size of the occipital condyle and is oriented vertically.

The basioccipital and supraoccipital contribute to the ventral and dorsal margins of the foramen magnum in *Allosaurus fragilis* in contrast to the illustrations by Madsen (1976). The supraoccipital and basioccipital are excluded from the foramen magnum in *Sinraptor dongi* (IVPP V10600) and *Acrocanthosaurus atokensis* (Eddy & Clarke, 2011). The supraoccipital contributes a small area along the dorsal surface of the foramen magnum in *Acrocanthosaurus atokensis* (Eddy & Clarke, 2011; Currie & Carpenter, 2000).

**External mandibular fenestrae**—Each external mandibular fenestra (Figs. 4 and 5) is positioned on the lateral surface of the mandibular between the dentary, angular, and surangular. The prearticular can be seen interiorly along with the extreme posterior surface of the splenial (MOR 693). Externally, the anterior surface of the external mandibular fenestra is formed by the dentary which includes a concave excavation posteriorally that forms the anterodorsal one fourth of the fenestra. The remainder of the dorsal posterior margins of the fenestra is formed by the surangular which also forms the posterior margin of the fenestra. Ventrally, the angular forms most of the margin of the fenestra. Medially, the external mandibular fenestra opens into a larger fenestra created by the prearticular coronoid and surangular which is often called the internal mandibular fenestra and functions as an insertion point and expansion chamber for the mandibular musculature (Fig. 7).

The external mandibular fenestra is much smaller than those in *Sinraptor dongi* (Currie & Zhao, 1993a) and *Acrocanthosaurus atokensis* (Eddy & Clarke, 2011). The external mandibular fenestra is indistinguishable between species of *Allosaurus*, in spite of the extremely small external mandibular fenestra figured in Madsen (1976).

### Bones of the dermatocranium

**Premaxillae**—The body of each premaxilla (Figs. 4–8) is rectangular. Its lateral surface bears scattered foramina for branches of the medial ethmoidal nerve and the subnarial artery (Currie & Zhao, 1993a). The nasal or supranarial process of the premaxilla extends one quarter of the length of the dorsal margin of the external nares. It fits into a notch in the nasal and is overlapped medially and laterally by that bone. The subnarial process of the premaxilla extends along just over one half the length of the ventral margin of the external nares, contacting the subnarial process of the nasal and excluding the maxilla from the narial margin. The contact with the body of the maxilla is nearly vertical.

The medial surface for the contact with the opposite premaxilla is flat and smooth along the interpremaxillary suture (Fig. 7). The contact between the premaxillae is flat, as also evidenced by the lack of fusion in complete skulls, along with the fact that the premaxillae are often separated along their midline suture (DINO 11541 and MOR 693). The supranarial process inserts posteriorly into the anterior dorsal process of the nasal that laps laterally and medially around the premaxilla. Only one premaxillary tooth is visible in medial view in DINO 11541; there are five alveoli. MOR 693 also has five alveoli. There are four erupted teeth in the left premaxilla whereas the right one preserved two. The premaxillary paradental plates are fused as in most theropods; although they are not fused in *Sinraptor dongi* (Currie & Zhao, 1993a). Fusion of paradental plates occurs early in ontogeny of *Allosaurus fragilis*, as shown in the juvenile premaxillae UMNH VP 3113 and UMNH VP 9268.

**Maxillae**—The maxilla (Figs. 4–8) contacts the premaxilla, nasal, lacrimal, and jugal in lateral view (Figs. 4–6) and with the vomer and palatine medially (Figs. 7 and 8). Posteriorly it extends nearly to the posterior margin of the orbit. This is the case in both DINO 11541 and MOR 693 as evidenced by the sutural contact on the jugals. The anterior process of the maxilla is short and does not extend as far anteriorly as in spinosauroids and other basal theropods such as, *Piatnitzskysaurus*, *Torvosaurus, Neovenator* and *Yangchuanosaurus* (Bonaparte, 1986; Brusatte, Benson & Hutt, 2008; Rauhut, 2003; Dong, Zhou & Zhang, 1983). The short anterior process with a slightly concave anterodorsal margin is more pronounced than in *Sinraptor* or *Acrocanthosaurus*. The maxilla makes up only the posterior margin of the perinarial fossa. The superior labial foramina for the superior alveolar nerve and maxillary artery (Currie & Zhao, 1993a) are immediately dorsal to the ventral margin of maxilla on the lateral surface. There are sixteen maxillary alveoli.

In *Allosaurus jimmadseni* the nasal process of the maxilla rises at an angle of about 35°. It is long, tapering, and forked posteriorly for reception of the lacrimal. The pneumatic excavation of the nasal ramus (Witmer, 1997a, 1997b) is very shallow, poorly defined, and imperforate. It is adjacent to and associated with the pneumatic foramina in the nasal. It resembles *Allosaurus fragilis*, *Carcharodontosaurus saharicus*, *Monolophosaurus jiangi* (Brusatte, Benson & Xu, 2010), *Neovenator salerii* (Hutt, Martill & Barker, 1996; Brusatte, Benson & Hutt, 2008), and *Giganotosaurus carolinii*. In *Acrocanthosaurus atokensis* the pneumatic excavation is better defined but still imperforate. Both *Sinraptor* and *Yangchuanosaurus* have deep, well-defined excavations, which are pierced by multiple pneumatic fenestrae, a condition unknown in other allosauroids (Currie & Zhao, 1993a; Gao, 1992).

The posterior jugal process of the maxilla thins to slot into a groove on the anterolateral surface of the jugal. This process has only a slight ventral arc in *Allosaurus jimmadseni*, unlike the pronounced ventral deflection of the posteriormost portion of the jugal process in *Allosaurus fragilis* (Figs. 4, 5 and 13).

In medial view, the nasal process of DINO 11541 is covered by matrix at its dorsal and anteroventral regions, although part of its contact with the subnarial process of the nasal is visible (Figs. 7 and 8). The postantral strut is obscured by the anterior process of the pterygoid. The maxillary antrum is large and elliptical, and much larger than in *Allosaurus fragilis* (Madsen, 1976; Gilmore, 1920). The promaxillary strut and vestibular bulla are also visible. Both the maxillary antrum and the promaxillary recess are filled with sediment. The anteromedial process of the maxilla abuts against the medial surface of the premaxilla, overlapping and hiding the medial surface of the suture between the premaxilla and maxilla. The medial surface of the rostromedial process has two distinct grooves, as in *Sinraptor dongi*.

In medial view the maxillary paradental plates are fused to each other and there is a groove for the paradental artery along their contact with the maxilla (Fig. 7). The paradental plates are tallest and widest anteriorly and become smaller posteriorly. Maxillary paradental plates are also fused in *Allosaurus fragilis*, *Neovenator salerii*, *Acrocanthosaurus* and *Giganotosaurus carolinii*. The paradental plates are unfused in *Piatnitzkysaurus*, *Monolophosaurus*, and *Carcharodontosaurus*.

In medial view, the tips of the dentary teeth are at the level of the ventral margin of the premaxillary and maxillary paradental plates in the occluded left sides of both DINO 11541 and MOR 693. The medial surface of the maxilla does not have depressor pits to accommodate the anterior dentary teeth as seen in many tyrannosauroids including *S. clevelandi*, *Xiongguanlong baimoensis, Lythronax argestes* and *Tyrannosaurus rex* (Loewen et al., 2013).

**Nasals**—The nasals (Figs. 4–7 and 11) are elongate, splint-like bones with triangular cross-sections along most of their length. The medial contact between the nasals forms a long, thin, surface that is smooth. The result is a loose internasal contact that may have allowed for some movement between the nasals. This contact is similar in *Allosaurus fragilis*, as evidenced by complete skulls (DINO 2560; BYU 9466) in which the nasals are separated by a sediment-filled gap. Unfused nasals are primitive in Dinosauria and are found in nearly all basal tetanurans including: *Monolophosaurus* (Currie & Zhao, 1993a), *Sinraptor dongi*, *Yangchuanosaurus* (Dong, Zhou & Zhang, 1983), *Carcharodontosaurus*, and *Acrocanthosaurus*.

Anteriorly, the premaxillary process of the nasal forms two thirds of the dorsal surface of the external naris. The premaxillae insert into a groove on the anterior dorsal surface. The premaxillary processes of the nasal intervene between the articulated premaxillae medially and the lateral surface of the premaxillary process of the nasal overlaps the nasal process of the premaxilla laterally. The subnarial process of the nasal, meets the subnarial process of the premaxilla ventrally to exclude the maxilla from the naris. There is no indication of a narial fossa on the nasal.

The dorsal surface of the nasals is smooth in *Allosaurus jimmadseni*, as they are in *Allosaurus fragilis*, *Neovenator*, *Sinraptor dongi*, and *Acrocanthosaurus*. The nasals in *Monolophosaurus* are part of the sagittal crest and are arched, coossified (although a midline suture can still be traced) and have a rugose surface texture (Currie & Zhao, 1993a). In *Carcharodontosaurus* the nasals are unfused, although they have a rugose texture on their dorsal surface. The nasals of *Yangchuanosaurus shangyuensis* are also reported to have a rugose texture (Dong, Zhou & Zhang, 1983).

The nasal overhangs and forms the dorsal margin of antorbital fossa. Its dorsolateral edge is upturned, forming low, but distinct, bilateral nasal crests. This crest is lowest anteriorly and highest (22 mm) slightly anterior to the cornual process or horn of the lacrimal. These crests are present in *Allosaurus jimmadseni* (DINO 11541, MOR 693, SMA 0005, and BYU 5253) as well as in *Allosaurus europaeus* (ML 415) (Mateus, Walen & Antunes, 2006). This dorsolateral nasal crest is not present in *Allosaurus fragilis* (USNM 4734, DINO 2560, BYU 9466, UMNH VP 7748, and UMNH VP 9149- see figure 11), and is not present in *Sinraptor dongi*, *Neovenator*, *Acrocanthosaurus* or *Carcharodontosaurus*. Instead, these taxa exhibit a thickened blocky lateral overhang above the antorbital fossa. In *Yangchuanosaurus shangyuensis* the posterior half of the dorsal surface of the nasal overhangs the antorbital fossa but the anterior half is nearly vertical and its lateral surface bears several large fossa which are probably pneumatic (Dong, Zhou & Zhang, 1983; Dong, 1987). The posterolateral margin of the nasal crest continues to form a shelf that overhangs the anterior process of the lacrimal.

The nasal forms part of the antorbital fossa and overhangs that cavity. Beneath this shelf there is a deep elliptical pocket with a pneumatic fenestra, the nasal pneumatic recess, near the dorsal surface of the antorbital fossa. This fenestra penetrates the nasal and is subdivided into two recesses. Immediately posterior to this recess there is a slight depression which does not penetrate the nasal.

The nasal pneumatic recesses are variably developed in *Allosaurus fragilis*. The most common condition is with two recesses per nasal. However, in DMNH 2419 there is only one pneumatic recess while in USNM 4734 there are two recesses in the right nasal and three in the left. Pneumatic features can be variable in development and asymmetry is not unexpected (Currie & Zhao, 1993a; Witmer, 1997a, 1997b; Britt, 1993). We interpret the variation in the nasal pneumatic recesses in *Allosaurus* due individual variation and of no systematic implication.

Nasal pneumatic recesses are variably developed in allosauroids. In *Monolophosaurus* the nasals are highly modified to form a sagittal crest, and there are three very large, lateral pneumatic recesses on each nasal (Zhao & Currie, 1993). *Sinraptor dongi* has two recesses per side (Currie & Zhao, 1993a), as apparently does *Sinraptor hepingensis* (Gao, 1992). No nasal pneumatic recesses are shown in *Carcharodontosaurus*. In *Acrocanthosaurus* there are depressions in the nasal, but they do not penetrate the bone.

The nasal in *Allosaurus jimmadseni* contacts the frontal the prefrontal posteriorly, and the base of the cornual process of the lacrimal posterolaterally. The suture with the frontal is irregular and interdigitate.

**Lacrimals**—The lacrimals (Figs. 4–7) form part of dorsal margin and the posterodorsal corner of the antorbital fossa and the antorbital fenestra. The antorbital fossa extends onto the lateral surface of the lacrimal from the posterior end of the lateral surface of the nasal above the lacrimal vacuity and extends ventrally from there. There is an anterior deflection on the mid-point of the ventral ramus of the lacrimal. The antorbital fossa extends anterior to the posteriormost point of the antorbital fenestra, as the antorbital fenestra is tucked medially behind this inflection, then curves posteriorly to meet the jugal ventrally. The preorbital ramus is narrowest at midheight, just ventral to the anterior inflection of the antorbital fossa. The preorbital ramus forms part of the medial wall of the antorbital cavity. Ventrally, the lacrimal has a long contact with maxilla and a short contact with the jugal. In both DINO 11541 and MOR 693 the lacrimal and maxilla exclude the jugal from contributing to the antorbital fenestra at least laterally. Instead, the jugal does contribute to the antorbital fossa. This is also the case in *Allosaurus fragilis*, but this is contrary to the published description of *Allosaurus europaeus*, in which the jugal intervenes between the lacrimal and maxilla (Mateus, Walen & Antunes, 2006).

The anterior ramus of the lacrimal is overlapped anterolaterally by a thin process of the nasal, and the ventral end of the preorbital ramus is overlapped laterally by the posterior end of the nasal process of the maxilla. Medially, it contacts the frontal and prefrontal.

The cornual process or lacrimal horns are sub-circular in outline in lateral view. This differs slightly from MOR 693 and SMA 0005, which have more angular cornual processes. A lacrimal cornual process is absent in most theropods. It is variably developed even within Tetanurae. No lacrimal is known for *Piatnitzkysaurus* or *Szechuanoraptor*. In *Monolophosaurus* the dorsal extension of the lacrimal is incorporated into the sagittal crest, along with the premaxillae and nasals (Zhao & Currie, 1993). However, the crest component of the lacrimal in *Monolophosaurus* is not extensive and is restricted to the caudolateral parts of the crest. The shape of the dorsal extension in *Monolophosaurus* is reminiscent of that in *Cryolophosaurus* in being dorsoventrally taller than anteroposterally long. *Cryolophosaurus* has a pneumatic lacrimal and there is a narrow vertical projection at the top of the lacrimal which appears to be a lacrimal cornual process (Hammer & Hickerson, 1994; Smith et al., 2007). The cornual process is low in *Sinraptor dongi*, *Sinraptor hepingensis* (Currie & Zhao, 1993a), *Acrocanthosaurus atokensis*, and *Carcharodontosaurus saharicus*. It is slightly dorsally taller in *Yangchuanosaurus shangyuensis* (Dong, Zhou & Zhang, 1983) and *Giganotosaurus carolinii* (Coria & Salgado, 1995). A well-developed cornual process, longer than high, and substantially projected above the skull table is found only in *Allosaurus* among allosauroids.

At the base of cornual process, on its lateral surface within the antorbital fossa, there is a lacrimal recess and vacuity that splits into two pneumatic recesses, which in turn penetrate into the cornual process. The lacrimal recess is vertically elliptical and penetrates medial to the lateral surface of the lacrimal along its dorsal and posterior margins. There is a ridge that runs vertically from between the recesses on the lateral surface of the lacrimal vacuity. This ridge disappears about half way to the dorsal margin of cornual process.

Lacrimal pneumatic recesses are common in theropods. Sereno (1997, 1999) considers this feature as a synapomorphy for Tetanurae. *Cryolophosaurus* has a lacrimal that is described as pneumatic but lacks a large pneumatic recess in lateral view (Hammer & Hickerson, 1994; Smith et al., 2007).

**Prefrontals**—The prefrontals (Figs. 5 and 6) are arcuate bones that contact and intervene between the lacrimal (laterally) and frontal and nasal (medially) in dorsal view. In lateral view, the posterior process of the prefrontal is visible at the anteromost dorsal portion of the orbit and makes up nearly as much of the top of the orbit as does the frontal. The medial surface contacts the nasal and frontal in nearly equal proportions. The frontal interfingers with both the nasal and prefrontal. The prefrontal forms over half of the dorsal orbital notch in dorsal view.

The prefrontal of *Allosaurus jimmadseni* is indistinguishable from that of *Allosaurus fragilis* and is more splint-like in dorsal view than the wedge-shaped prefrontal of *Sinraptor dongi* (Currie & Zhao, 1993a) and *Acrocanthosaurus atokensis* (Eddy & Clarke, 2011).

**Frontals**—The frontals (Figs. 5–8) of *Allosaurus jimmadseni* are unfused, with a straight anterior interfrontal suture along the anterior three quarters of the element and the posterior quarter of the interfrontal suture tightly interdigitated. The suture with the parietal is also highly interdigitated. In medial view, a thin (1 mm thick) process of the frontal extends about 70 mm beneath the posterior end of the nasal and forming a contact surface for that nasal.

Dorsally, a pair of curved ridges marks the margins of the *M. pseudotemporalis* origin on the frontal, forming the anterior end of the supratemporal fossa. These ridges continue onto the parietal; however, they do not meet to form a peaked sagittal crest. Instead there is a horizontal surface forming a narrow (15 mm) flat skull table between the supratemporal fossae and fenestrae. There is a narrow frontal incisure (Russell, 1970) and the frontals form a small part of the orbital margin. The frontals are widest across the postorbital process. The postorbital process of the frontal is supported posteroventrally by the lateral ramus of the laterosphenoid.

The lateral sutural surface of the frontal reveals an anterior half that is thin and underlies the nasals and a posterior half that is dorsoventrally thicker to support the postorbital articulation. These two regions of the lateral articular surface of the frontal are separated by a transitional area with a crescentic notch to support the articulation with the prefrontal.

The frontals are unfused in all allosauroids, except for *Carcharodontosaurus* (Sereno et al., 1996) and *Acrocanthosaurus atokensis* (OMNH 10146 *contra* Stovall & Langston (1950)), and *Giganotosaurus* (Coria & Currie, 2002) where the frontals are fused both with one other as well as with the parietals. The frontal of *Piatnitzkysaurus* (Bonaparte, 1986) shares features with more basal theropods, such as a blunt anterior margin, a very small subnasal process, lacking a pronounced postorbital process, and making a major contribution to the orbital rim. In these features, *Piatnitzkysaurus* more closely resembles *Coelophysis rhodesiensis* (Raath, 1977) and *Eustreptospondylus oxoniensis* than allosauroids. *Monolophosaurus* is primitive in its subequally wide and long frontal contribution to the skull table (Zhao & Currie, 1993; Brusatte, Benson & Xu, 2010). In *Cryolophosaurus* (Hammer & Hickerson, 1994; Smith et al., 2007) the frontals make a larger contribution to the orbital rim than any other basal tetanuran except *Monolophosaurus*. In addition, the frontals in *Cryolophosaurus* are uniquely developed into a large, anteriorly concave, transverse crest (Hammer & Hickerson, 1994; Smith et al., 2007).

The interfrontal suture in nearly all allosauroids is straight anteriorly and interdigitated posteriorly, with the exception of *Monolophosaurus jiangi*, where the suture is relatively straight.

**Parietals**—The parietals contact the frontals anteriorly, each other medially, the postorbital and squamosal laterally, the laterosphenoid and prootic ventrally and the supraoccipital and exoccipital posteriorly (Figs. 4–10). They form the majority of the dorsal surface of the endocranial cavity which held the brain. The frontoparietal suture is anterodorsally inclined in lateral view and highly interdigitated and do not disarticulate in adult specimens. In *Allosaurus jimmadseni*, as in many tetanurans, the interparietal suture is strongly interdigitated and the parietals typically stay articulated long after other skull bones disarticulate (as seen in DINO 11541). The dorsal surface of the parietals is extensively invaded by the *M. pseudotemporalis* and the margin of this invasion is marked by a ridge that continues onto the frontals forming the margin of the supratemporal fossa. Laterally, the parietal contacts the laterosphenoid and prootic with interdigitated sutures. Posteriorly, the parietals rise dorsally to form the transverse parietal crest, the middle posterior surface of which surrounds the supraoccipital. Laterally, on the posterior surface, the parietal overlaps the dorsomedial surface of the paroccipital process.

In occipital view, the transverse parietal crest is well developed and extends dorsal to the skull table. The broad, flat, posterior surface of the transverse parietal crest is the origin for the *M. longissimus capitis* (Raath, 1977). There is a median notch in the transverse parietal crest, dorsal to the supraoccipital postnuchal crest. In occipital view, the parietals contact the squamosals laterally and the supraoccipital medially and ventrally. A thin slip of the parietal separates the squamosal and supraoccipital along more than half of the length of each paroccipital process.

Parietal articulation is co-related to ontogenetic maturity and is variable across theropoda. In *Allosaurus jimmadseni*, *Allosaurus fragilis*, *Monolophosaurus jiangi* (Zhao & Currie, 1993), *Piatnitzkysaurus* (Bonaparte, 1986), *Sinraptor dongi* (Currie & Zhao, 1993a), *Sinraptor hepingensis* (Gao, 1992), and *Yangchuanosaurus shangyuensis* (Dong, Zhou & Zhang, 1983) the parietals are locked tightly together in adults, but there is still an obvious visible interdigitate suture between them. The parietals are fused and their interparietal suture is obliterated in *Acrocanthosaurus* (OMNH 10146 and NCSM 14345) (*contra* Stovall & Langston, 1950), *Giganotosaurus* (MUCPv-CH-1) (Coria & Currie, 2002) and *Carcharodontosaurus* (SGM-Din 1) (Sereno et al., 1996).

Excluding *Allosaurus*, there is no deep notch in the parietals above the supraoccipital in other allosauroids. The parietals meet above the nuchal crest of the supraoccipital in *Acrocanthosaurus* (Eddy & Clarke, 2011), *Carcharodontosaurus* (Sereno et al., 1996), *Monolophosaurus* (Zhao & Currie, 1993), *Sinraptor dongi* (Currie & Zhao, 1993a), and *Sinraptor hepingensis* (Gao, 1992).

In *Allosaurus*, *Acrocanthosaurus* (Eddy & Clarke, 2011), and *Sinraptor dongi* (Currie & Zhao, 1993a) there is a long process of the supraoccipital which separates the squamosal from the dorsomedial edge of the paroccipital process. In *Monolophosaurus* (Zhao & Currie, 1993) the parietals do not exclude the squamosals from their contact with the exoccipital process. The condition is unknown or unreported for other allosauroids. In primitive theropods such as *Herrerasaurus* (Sereno & Novas, 1993) and *Coelophysis rhodesiensis* (Raath, 1977) the parietals run the entire length of the dorsal margin of the exoccipital process. In *Coelophysis bauri* (Colbert, 1989) there is a small contact between the squamosal and the dorsal margin of the exoccipital.

In occipital view, the parietals of *Allosaurus jimmadseni* are slightly taller than wide, whereas in *Allosaurus fragilis*, *Monolophosaurus* (Zhao & Currie, 1993), and *Sinraptor dongi* (Currie & Zhao, 1993a) they are wider than tall, the primitive theropod condition.

**Squamosals**—The squamosals (Figs. 4–10) form the dorsal and dorsoposterior half of margin of the lateral temporal fenestrae. The descending ramus extends more than half the height of the lateral temporal fenestra and contacts the quadratojugal, excluding the quadrate from the fossa. The anterior margin is strongly convex and forms the dorsal surface of the lateral temporal fenestra. The lateral surface of the squamosal has vertical striations immediately dorsal to its contact with the quadratojugal.

*Allosaurus jimmadseni* has a well-developed cotylus for the head of the quadrate. There is also a well-developed posterior process of the squamosal that runs along the posteriodorsal surface of the quadrate. This process is weakly notched at its distal end. The horizontal ramus of the squamosal forks to receive the posterior process of the postorbital. The ventral part of this bifurcation is narrow, whereas the dorsal part is bigger and wide in occipital view. At the base of the bifurcation the squamosal has a short narrow shelf that projects laterally.

In some theropods, such as *Herrerasaurus* (Sereno & Novas, 1993) and *Coelophysis rhodesiensis* (Raath, 1977) the quadrate forms part of the margin of the lateral temporal fenestra, although it does not in *Coelophysis bauri* (Colbert, 1989) or *Coelophysis kayentakatae* (Rowe, 1989). In all allosauroids for which the region is known, the quadrate is excluded from the fenestra margin (*Allosaurus fragilis*, *Allosaurus europaeus*, *Acrocanthosaurus*, *Cryolophosaurus*, *Sinraptor dongi*, *Sinraptor hepingensis*, and *Yangchuanosaurus shangyuensis)*. The ventral ramus of the squamosal projects down the anterior margin of the quadrate less than half the height of the lateral temporal fenestra in *Cryolophosaurus*, *Sinraptor dongi*, *Sinraptor hepingensis*, and *Yangchuanosaurus shangyuensis*. It reaches at least half the height of the fenestra in *Allosaurus jimmadseni*, *Allosaurus fragilis*, *Allosaurus europaeus* and *Monolophosaurus*. In these three taxa the quadratojugal also projects slightly anteriorly, restricting the lateral temporal fenestra. Only *Allosaurus fragilis*, *Allosaurus europaeus* and *Allosaurus jimmadseni* have striations on the lateral surface of the descending process of the squamosal, which appears to be a synapomorphy for the genus.

**Postorbitals**—The postorbital (Figs. 4–6 and 10) forms two thirds of the posterior portion of the orbit and the dorsal third of the anterior margin of the laterotemporal fenestra. It contacts the frontal but does not contact the lacrimal or prefrontal. It is separated from the lacrimal by the prefrontal and the frontal. The ventral process of the postorbital process tapers ventrally. Laterally, it overlaps the ascending process of jugal in a lap joint lateral to the jugal. Dorsally, the jugal suture forms a “U”-shaped cross-section with the postorbital wraping medially and laterally around the anterodorsal part of the dorsal process of the jugal. This contact is loose, as evidenced by the slight separation between these bones in *Allosaurus jimmadseni*, and complete skulls of *Allosaurus fragilis* (DINO 2560; BYU 9466) and in *Allosaurus europeaus* (Mateus, Walen & Antunes, 2006). As in *Allosaurus fragilis*, the postorbital process extends about two-thirds the height of the orbit, whereas in *Allosaurus europaeus* it continues to almost the ventral extent of the orbit. The postorbital does not have an anterior expansion or subocular process constricting the orbit.

The ventral process of the postorbital reaches nearly to the level of the ventral margin of the orbit in *Coelophysis kayentakatae* (Rowe, 1989), *Cryolophosaurus* (Hammer & Hickerson, 1994; Smith et al., 2007), *Monolophosaurus*, and *Allosaurus europeaus* (Pérez-Moreno et al., 1999) but is slightly shorter in *Herrerasaurus* (Sereno & Novas, 1993), *Coelophysis bauri* (Colbert, 1989), *Coelophysis rhodesiensis* (Raath, 1977) and *Yangchuanosaurus shangyuensis* (Dong, Zhou & Zhang, 1983). Among allosauroids, a condition similar to that in *Allosaurus fragilis* and *Allosaurus jimmadseni* is present in *Sinraptor dongi*, *Sinraptor hepingensis* (Gao, 1992) and *Acrocanthosaurus*, *Eocarcharia*, *Concavenator, Giganotosaurus*, and *Carcharodontosaurus*.

The lateral surface of the postorbital along the posterodorsal margin of the orbital margin is thickened and roughened but does not form a postorbital boss as in some tyrannosauroids. This differs from the smooth condition present in *Saurophaganax maximus*, and to some extent *Aerosteon riocoloradense*. A more pronounced ornamentation in this region is present in *Sinraptor dongi*, *Sinraptor hepingensis* (Gao, 1992), and *Yangchuanosaurus shangyuensis* (Dong, Zhou & Zhang, 1983), *Acrocanthosaurus*, *Eocarcharia*, *Concavenator, Giganotosaurus*, and *Carcharodontosaurus*.

There is no suboccular projection into the orbit in *Allosaurus jimmadseni*, in contrast to the more derived condition of the postorbital projecting laterally as a shelf which projects into the orbit below the eyes, as is partially present in *Acrocanthosaurus*, and pronounced in *Carcharodontosaurus*, *Giganotosaurus*, *Sinraptor dongi*, *Sinraptor hepingensis* (Gao, 1992), and *Yangchuanosaurus shangyuensis* (Dong, Zhou & Zhang, 1983).

Anterior expansions of the postorbital which constrict the orbit occur independently in abelisaurids, some allosauroids, and some tyrannosaurids (Chure, 2000b). The primitive condition for the orbit is to be unrestricted as in *Saurophaganax*. However, among allosauroids it is constricted in *Acrocanthosaurus*, *Giganotosaurus*, and *Carcharodontosaurus*.

**Jugals**—The jugal (Figs. 4–8, 10 and 12) of *Allosaurus jimmadseni* forms the ventral margin of the orbit and the ventral one third of the posterior margin of the orbit. It also forms the anterorventral two thirds of the anterior and ventral margins of the lateral temporal fenestra. The ascending ramus extends more than half the height of the postorbital bar and is overlapped laterally by the descending process of the postorbital. Posteriorly, the jugal is bifurcated for reception of the quadratojugal. The dorsal ramus of the quadratojugal forms more than half of the ventral border of the lateral temporal fenestra. In turn, the ventral ramus of the quadratojugal extends posteriorly well beyond the lateral temporal fenestra, but just barely avoids contact with the quadrate. Ventral to the orbit, the lateral surface of the jugal is thickened and sculptured.

The ventral margin of the jugal is straight, as in most basal theropods, and does not show the ventral deflection of the jugal characteristic of *Allosaurus fragilis* and *Allosaurus europaeus* and is one of the diagnostic features distinguishing *Allosaurus jimmadseni* from those species (Fig. 12).

Anteriorly, the jugal overlaps the maxilla medially and has a short contact with the posteroventral edge of lacrimal. The jugal appears to be excluded from the margin of the antorbital fenestra by the maxilla and the lacrimal. This is difficult to interperate because both the lacrimal and the jugal are thin in this region and subject for microfractures that make the suture cryptic. In the case of MOR 693 the skull has been broken and repaired and the preservation reveals many cracks. It is difficult to interperate in SMA 0005 due to disarticularion and fractured surface preservation. There is no specimen of *Allosaurus fragilis* that shows clear contribution of the jugal to the antorbital fenestra. In both species it is possible there is a limited contribution of the jugal to the antorbital fenestra but we interperate it to be excluded. Further study and specimens may addrerss this character more closely. There is a clear contribution of the jugal to the antorbital fenestra in *Allosaurus europeaus* (Mateus, Walen & Antunes, 2006). The jugal fossa forms part of the antorbital fenestraa in many theropods. Among basal theropods, it is does not form part of the fenestra in *Coelophysis bauri* (Colbert, 1989), *Coelophysis rhodesiensis* (Raath, 1977), and *Coelophysis kayentakatae* (Rowe, 1989), but it does so in *Herrerasaurus ischigualastensis* (Sereno & Novas, 1993).

In *Allosaurus fragilis* and *Allosaurus jimmadseni* the jugal appears to be excluded from the margin of the internal antorbital fenestra and from forming part of the medial wall of the antorbital fossa; as a result, there is no jugal recess. Among allosauroids the jugal recess is present in *Acrocanthosaurus atokensis*, *Carcharodontosaurus*, (Hammer & Hickerson, 1994; Zhao & Currie, 1993), *Sinraptor dongi* (Currie & Zhao, 1993a), *Sinraptor hepingensis* (Gao, 1992), *Yangchuanosaurus shangyuensis* and *Yangchuanosaurus shangyouensis* (Dong, Zhou & Zhang, 1983). The jugal forms part of the margin of the antorbital fenestra in *Acrocanthosaurus*, *Carcharodontosaurus*, *Monolophosaurus*, *Sinraptor dongi*, *Sinraptor hepingensis* (Gao, 1992), and *Yangchuanosaurus shangyuensis* (Dong, Zhou & Zhang, 1983).

There is no pneumatic foramen on the lateral surface of the jugal of *Allosaurus jimmadseni*. In *Allosaurus fragilis* the lateral surface is often pierced by several small neurovascular foramina. Although pneumatization in the vertebral column often occurs by utilizing and enlarging such foramina (Britt, 1993), the foramina on the lateral surface of the jugal in *Allosaurus* are small, and not pneumatic (*contra* Currie & Zhao, 1993a and *contra* Witmer, 1997a, 1997b). The jugal in *Allosaurus* is mediolaterally narrow and does not have large internal pneumatic cavities. The jugal pneumatic recess (Witmer, 1997a, 1997b) occurs within the jugal fossa and leads to pneumatic cavities within the jugal. Although a jugal recess is not present in *Allosaurus* and *Cryolophosaurus* (Hammer & Hickerson, 1994; Long, 1998; Tomida, 1998) it is present in *Monolophosaurus* (Zhao & Currie, 1993), *Acrocanthosaurus* (Stovall & Langston, 1950), *Carcharodontosaurus* (Sereno et al., 1996), and *Yangchuanosaurus shangyuensis*. The condition cannot be determined for *Yangchuanosaurus shangyouensis*, even though a jugal is preserved (Dong, Zhou & Zhang, 1983; Dong, 1987). Jugal recesses also occur in other theropods like *Deinonychus antirrhopus*, *Afrovenator abakensis*, and tyrannosauroids (Witmer, 1997a, 1997b).

In *Allosaurus jimmadseni* the ascending ramus of the jugal extends more than half the height of the postorbital bar and is overlapped laterally by the descending process of the postorbital. The lateral surface of the ascending process is imperforate, as in *Allosaurus* and other theropods. However, there are some interesting, albeit abnormal, specimens of *Allosaurus fragilis*. In YPM 1890, (the holotype of *Creosaurus atrox*, a junior subjective synonym of *Allosaurus fragilis*) there is a large elliptical hole in the middle of the lateral surface of postorbital process of the right jugal (Chure, 2000a). This hole extends ventromedially into the jugal. The bone texture around this hole is normal. Unfortunately, the left jugal is not preserved in this specimen. In AMNH 5753 (Chure, 2000a) there is a large elliptical depression with a raised rim on the ascending process of the left jugal, but it does not penetrate the jugal. The bone texture around this depression is normal. The right jugal is not preserved in this specimen. All other *Allosaurus* jugals examined in this study lack a foramen or depression on the postorbital process of the jugal. In both YPM 1890 and AMNH 5753 the anomalous features are not within the antorbital fossa. Based on this, the features are not related to the craniofacial pneumatic system and we interpret them as either individual variation or pathological.

**Quadratojugals**—The quadratojugal (Figs. 4–6, 8 and 10) forms the posterioventral corner of the lateral temporal fenestra. It extensively overlaps the lateral surface of the quadrate and jugal. The anterior ramus tapers and extends roughly to the anterior extent of the lateral temporal fenestra. There is no posterior ramus as in tyrannosauroids.

The ascending process is anteroposterally broad and its posterior margin slopes caudodorsally. The dorsal ramus contacts the squamosal dorsally. The quadratojual laps onto the lateral surface of the quadrate. The suture with the quadrate runs along the apex of a pronounced, vertically directed ridge. The quadratojugal does not contribute to the process which projects into the posterior end of the lateral temporal fenestra.

Among early theropods, the quadratojugal does extend anterior to the lateral temporal fenestra (*Coelophysis* (Colbert, 1989), *Herrerasaurus* (Sereno & Novas, 1993), *Coelophysis rhodesiensis* (Raath, 1977), *Coelophysis kayentakayatae* (Rowe, 1989), *Cryolophosaurus* (Hammer & Hickerson, 1994), and *Monolophosaurus* (Zhao & Currie, 1993)). The allosauroidea condition is variable. The process does not extend anterior to the lateral temporal fenestra in *Acrocanthosaurus*, *Carcharodontosaurus*, *Sinraptor dongi*, and *Sinraptor hepingensis* (Gao, 1992). It does extend beyond the lateral temporal fenestra in *Allosaurus* and *Yangchuanosaurus shangyuensis* (Dong, Zhou & Zhang, 1983).

The posterior base of the jugal ramus of the quadratojugal is proportionately deeper in *Allosaurus jimmadseni* than in most other large theropods, and more similar to *Allosaurus fragilis*, *Allosaurus europaeus* and *Yangchuanosaurus shangyuensis* (Dong, Zhou & Zhang, 1983). The jugal ramus tapers to a point anteriorly in all allosauroids in which it is known, with the exception of *Sinraptor dongi*, where it is forked (Currie & Zhao, 1993a).

Primitively, the ascending ramus of the quadratojugal is narrow and does not hide much, if any, of the lateral surface of the quadrate, although it does so more in *Herrerasaurus* (Sereno & Novas, 1993) than in *Coelophysis bauri* (Colbert, 1989), *Coelophysis rhodesiensis* (Raath, 1977), and *Coelophysis kayentakatae* (Rowe, 1989). The quadratojugal does not contact the squamosal in *Herrerasaurus* or *Coelophysis rhodesiensis*, but it does in *Coelophysis bauri* and *Coelophysis kayentakatae*. In allosauroids the quadratojugal contacts the squamosal in all taxa for which this part of the skull is known. The quadratojugal in allosauroids is usually tall and narrow, covering the anterior half of the quadrate. However, in *Allosaurus fragilis* (Madsen, 1976; Gilmore, 1920), *Allosaurus jimmadseni*, *Cryolophosaurus* (Hammer & Hickerson, 1994; Smith et al., 2007), and *Monolophosaurus* (Zhao & Currie, 1993) the quadratojugal covers proportionately more of the quadrate shaft than in *Allosaurus europaeus*.

**Ectopterygoids**—The ectopterygoid (Figs. 5–8) is a hook shaped element in dorsal view with a posteriorly concave margin. It is oriented nearly vertically and contacts the pterygoid medially and the jugal laterally. The ectopterygoid also contacts the posteriormost part of the posterior ramus of the maxilla, which is wedged between the jugal laterally and the ectopterygoid medially. The ectopterygoid forms the posterior margin of the post-palatine suborbital fenestra. There is a ventral pneumatic recess that covers the ventral surface of the ectopterygoid and extends onto the pterygoid. This pneumatic recess is most well developed along its anterior surface. The ectopterygoid is oriented at a high angle and produces a dorsally concave notch between the jugal and pterygoid. Consequently, the dorsal margin of the surangular fits within this notch between the jugal process and the main body of the ectopterygoid and the pterygoid, possibly functioning as a stop for the mandible as suggested by Ford (1997).

The ectopterygoid is well known in *Allosaurus fragilis* but poorly known in most other basal tetanurans. In *Sinraptor dongi* (Currie & Zhao, 1993a; Witmer, 1997a) and *Acrocanthosaurus* (Eddy & Clarke, 2011; Harris, 1998) the pneumatic ectopterygoid recess is larger and more extensively developed than *Allosaurus*, extending onto the jugal process. In *Sinraptor dongi* the recess exits through a large foramen medially to contact the jugal anteriorly (Currie & Zhao, 1993a).

**Pterygoids**—The pterygoid is the largest bone of the palate (Figs. 5 and 7–10) and forms the medial border of the suborbital fenestra. Its anterior process is triangular in medial view and tapers anteriorly. This process extends to the level of the middle of the maxillary antrum and separates the internal choanae along with the vomers. Along the ventral margin of the anterior two thirds of medial surface of this process there is a ridge and a shallow groove immediately above the ridge. This groove marks the contact with the vomer.

Posteriorly, there is a lateral process that has a long contact with the ectopterygoid. A ridge divides this process in two, with the posterior half flat and the anterior half concave. This concave region is confluent with the ectopterygoid recess of the ectopterygoid. The concave region of the lateral process of the pterygoid is not as deeply excavated as in *Sinraptor dongi* and *Coelophysis rhodesiensis* (Witmer, 1997a) and should not be considered a separate pterygoid pneumatic recess. However, this concave region is confluent with the ectopterygoid recess and probably housed part of a pneumatic diverticulum.

The contact with the basipterygoid process is tall, indicating that the contact was along the ventral and anterior surfaces of the basipterygoid processes. The articulation of quadrate and pterygoid is achieved by a broad overlap of the pterygoid wing of the quadrate and the sheet-like posterior quadrate-wing of the pterygoid. These two wings are broadly apressed and it is unclear to what degree they are fused to each other.

The pterygoid is unknown or undescribed in most allosauroids. In *Sinraptor dongi*, the ectopterygoid recess extended into the pterygoid and forms a distinct recess in that bone (Currie & Zhao, 1993a; Witmer, 1997a). Otherwise, the pterygoid of *Sinraptor* is similar to that of *Allosaurus jimmadseni* and *Allosaurus fragilis*. The ectopterygoid process of the pterygoid is much narrower in *Acrocanthosaurus atokensis* (Eddy & Clarke, 2011) than in *Allosaurus jimmadseni*.

**Palatines**—The palatines are paired elements between the posterior ramus of the maxilla and lap dorsally onto the midline elements of the palate, including the vomer and pterygoids (Figs. 5, 7 and 8). The palatine includes a lateral maxillary process on which a long, relatively straight suture contacts a large part of the medial surface of the posterior ramus of the maxilla. The palatine forms the posterior and medial margins of the internal choanae and the anterior margin of the infraorbital fenestra. The anterodorsal process is is visible above the vomer and pterygoid within the antorbital fenestra in medial view. Because the antorbital fossa is still filled with matrix, the dorsal process of the palatine cannot be seen in lateral view in DINO 1154; however, it is visible in medial and ventral views (Figs. 7 and 8) and it is visible in lateral, and ventral views in MOR 693 (Figs. 5 and 8). The palatine of DINO 11541 does not have a depression on the ventral surface of its body, whereas one is present only on the right side of MOR 693. There does not seem to be a large pneumatic recess on the lateral surface of the maxillary process of the palatine.

The palatine is unknown or undescribed in nearly all allosauroids. In medial view the palatine of *Allosaurus jimmadseni* does not differ significantly from that of *Allosaurus fragilis* (Madsen, 1976), *Sinraptor dongi* (Currie & Zhao, 1993a), and *Acrocanthosaurus atokensis* (Eddy & Clarke, 2011; Harris, 1998). Laterally, *Allosaurus jimmadseni* lack the pneumatic palatine recesses (Witmer, 1997a, 1997b) on the lateral surface which are present in *Sinraptor dongi* and *Acrocanthosaurus atokensis*.

**Vomers**—The vomers are not preserved in DINO 11541; however, they are present and articulated in MOR 693 (Figs. 5 and 8). The vomers are coossified and form a thin splint that intervenes between the pterygoids and the dorsal processes of the palatines. It is forked posteriorly and fused medially. It is dorsoventrally thickest and most medolaterally thin along the contact with the doral processes of the palatines. Anteriorly, the fused vomers mediolaterally widen, contact the palatal process of the premaxilla, and are visible in the ventral portion of the maxillary fenestra. They are indistinguishable from the vomers of *Allosaurus fragilis*. The vomers differ from *Sinraptor dongi* (Currie & Zhao, 1993a) in the midlength thickening along the dorsal process of the palatine.

### Bones of the chondrocranium

**Laterosphenoids**—The laterosphenoid (Figs. 7–10) contacts the parietal dorsally, where it projects laterally to support the postorbital process of that bone. Ventrally, it contacts the prootic and posteriorly, the parietal. A notch, the optic foramen (II), is located on the anterior and medial margin of the vertical ramus of the laterosphenoid. A slit on the anterior face of this ramus of the laterosphenoid is for the passage of cranial nerves III and IV. This is the pattern in *Allosaurus fragilis* (UMNH VP 16605). In *Sinraptor dongi* (Currie & Zhao, 1993a) and *Acrocanthosaurus* (OMNH 10146, NCSM 14345) these nerves exit more medially on the laterosphenoid than in *Allosaurus*. Otherwise, the laterosphenoid of *Allosaurus jimmadseni* is not significantly different from that of other allosauroids.

**Prootics**—The prootic (Figs. 5 and 7–9) is a complex bone which contacts the exoccipital/opisthotic, the frontal, laterosphenoid, and basisphenoid. The prootic overlaps the proximal third of the anterior surface of the paroccipital process. A foramen is located at the junction of the prootic, exoccipital, and the frontal, as in *Allosaurus fragilis* (e.g., UUVP 3304, 5583). This foramen sits in the floor of the dorsal tympanic recess. In some other theropods, such as *Shaochilong maortuenis* (IVPP V2885), this foramen is larger and leads into a subdivided internal pneumatic chamber. The foramen in *Allosaurus* may also be pneumatic, although it is not greatly enlarged. The prootic forms the anterior margin of the fenestra ovalis. At the base of the paroccipital process there is a short, well-developed ridge parallel to the long axis of the paroccipital process. This ridge marks the anterior margin part of the dorsal tympanic recess. In *Allosaurus fragilis* this ridge is lacking and the dorsal tympanic recess is a simple depression.

Ventral to the base of the laterosphenoid, the prootic forms an ala which projects laterally to the subotic recess of the basisphenoid (lateral basisphenoidal recess of (Chure & Madsen, 1998)). Because it is delicate, this ala is usually not preserved in *Allosaurus fragilis* and *europeas* skulls. However, it is completely preserved in *Allosaurus jimmadseni*.

The ala lateral to the subotic recess has received several different names; in *Piveteausaurus divesensis*—ala basisphenoidalis (Taquet & Welles, 1977) *Piatnitzkysaurus floresi*—alar process of the laterosphenoid (Bonaparte, 1986), *Allosaurus fragilis*—ala basisphenoidalis (Chure & Madsen, 1998), *Sinraptor dongi*—large crista prootica (Currie & Zhao, 1993a). This reflects the differing elemental composition of the ala as interpreted by various authors. This ala is present in *Acrocanthosaurus* (OMNH 10146, NCSM 14345).

The facial foramen (VII) is a large circular foramen posterior to the ventral edge of the laterosphenoid. A groove from this foramen leads anteroventrally across the prootic and then down the lateral face of the ala prootica. Below the facial foramen, and separated from it by a sharp ridge, is the foramen for the trigeminal nerve (V) is large and more anteriorly positioned on the lateral surface of the prootic, near the contact with the lateraosphenoid compared to the facial foramen (Fig. 9). Posteriorly, this foramen is separated from the smaller, more posteriorly positioned facial foramen by a relatively robust column of bone that is usually called the otosphenoidal crest or crist prootica. Dorsal to the facial foramen and near the laterosphenoid there is a small neurovascular foramen. A groove leads posteroventrally from this foramen. This pattern of foramina is like that in *Allosaurus fragilis* (UMNH VP 16605) and not significantly different from that seen in other allosauroids for which this part of the cranium is preserved such as *Sinraptor dongi* (Currie & Zhao, 1993a) and *Acrocanthosaurus atokensis* (OMNH 10146, NCSM 14345).

**Parasphenoid**—The parasphenoid (Figs. 5 and 7–9) is fused with the basisphenoid and forms the cultriform process. It was preserved in both articulated skulls of *Allosaurus jimmadseni* (DINO 11541, MOR 693) although the process is no longer present on MOR 693 due to damage to the skull while on loan. The cultriform process projects anteriorly, tapers anteriorly, and has a concave ventral outline. The dorsal surface of the cultriform process is angled anteroventrally with the overall orientation of the process pointing toward the anteroventral surface of the premaxilla.

The parasphenoid in *Allosaurus jimmadseni* (DINO 11541, MOR 693) is more elongated anteriorly than in *Allosaurus fragilis* from the Cleveland-Lloyd Quarry (Madsen, 1976). There is a large circular depression nears its base and a smaller, less well defined depression at midlength. Raath (1977) suggests that similar depressions in *Coelophysis rhodesiensis* are pneumatic in origin, but Witmer (1997b) suggests that they might be for a palatal protractor muscles.

The cultriform process is poorly known in allosauroids. Only a small part of the posterior region is preserved in *Acrocanthosaurus atokensis* (Stovall & Langston, 1950) and it is roughly figured in *Sinraptor hepingensis* (Gao, 1992) and only described in detail in *Sinraptor dongi* (Currie & Zhao, 1993a) which differs from *Allosaurus* in having a well-developed longitudinal trough on the ventral side of the process. This trough is not present in *Allosaurus fragilis* but is present as a narrow slit in *Ceratosaurus* (MWC 1).

**Basisphenoid**—The basisphenoid (Figs. 5 and 7–10) is posterioventral to and articulated with the parasphenoid. It forms the ventral part of the braincase. The basisphenoid is strongly articulated to the basioccipital lateral to the paired basitubera posteriorly and the forms the paired basipterygoids anteriorly. The basisphenoid is excluded from the basal tubera by the otoccipital and separated from the tubera by a deep groove. The basipterygoid processes are relatively. The articular surface for the pterygoid is on the lateral and anterior faces of the basipterygoid process. Both the basitubera and basipterygoids are separated by a medial groove. The basisphenoid recess is a medial excavation in the ventral part of the basisphenoid that is positioned along the midline between the basitubera and basipterygoids and is visible only in ventral view. The recess is twice as long as it is wide and is about as dorsoventrally deep as it is anteroposteriorly long. In lateral view, the occipital condyle, basal tubera and basipterygoid process form a right angle. The subotic recess is a deep recess in the middle of the lateral surface of the basisphenoid and, as in *Allosaurus fragilis*, houses the foramen for the internal carotid as well as several pneumatic foramina (Chure & Madsen, 1996). The margins of the subotic recess are more dileniated in *Allosaurus jimmadseni* than in *Allosaurus fragilis*.

In theropods the basisphenoidal recess is primitively visible only in ventral view. The primitive condition is present in *Piatnitzkysaurus floresi* (PVL 4073, *Monolophosaurus jiangi* (Zhao & Currie, 1993)), as it is in *Allosaurus fragilis and Allosaurus jimmadseni*. In *Acrocanthosaurus* (Eddy & Clarke, 2011; Chure & Madsen, 1998) the basisphenoid recess is enlarged and circular, and separates the basal tubera and is visible in posterior view. In *Sinraptor dongi* and *Acrocanthosaurus* the basisphenoid recess is also visible in posterior view. In *Sinraptor dongi* (Currie & Zhao, 1993a) the recess is more extensively exposed in posterior view, it does not separate the basal tubera and it is visible in posterior view because the basicranium has rotated posteroventrally. In *Acrocanthosaurus* the braincase is not posteroventrally rotated. Because of these morphological differences, it is uncertain as to whether the simple feature of the basisphenoid recess being visible in posterior view is homologous in *Acrocanthosaurus atokensis* and *Sinraptor dongi*. A more derived condition is achieved independently in the braincase of the tyrannosauroid *S. clevelandi* (UMNH VP 7818) (Chure & Madsen, 1998), the tyrannosauroid *Nanotyrannus lancensis* (Bakker, Williams & Currie, 1988), and the tyrannosaurid *Tyrannosaurus rex* (Osborn, 1912; Brochu, 2003).

The sella turcica isn’t exposed in DINO 11541 and is difficult to observe in MOR 693 because of its articulated braincase within the rest of the cranium.

The subsellar recess in *Allosaurus jimmadseni* is similar in position and development to the condition seen in *Allosaurus fragilis* (UMNH VP 16605, UMNH VP 21117, UMNH VP 23132), *Acrocanthosaurus* (OMNH 10146, NCSM 14345), *Ceratosaurus* (MWC 1), *Piatnitzkysaurus floresi* (PVL 4073), and *Sinraptor dongi* (IVPP V10600). The rostrolateral surface of the basisphenoid has an elliptical depression which is probably a pneumatic recess for a diverticulum of the anterior tympanic system. Chure & Madsen (1996) described ontogenetic changes in this recess in *Allosaurus fragilis*, where it invades the basipterygoid process in skeletally immature individuals, but in older individuals withdraws and is only a shallow depression on the rostrolateral surface of the basisphenoid. However, in *Allosaurus jimmadseni* this depression is well marked and invasive (i.e., overhung anteriorly). In *Allosaurus fragilis*, individuals the size of DINO 11541 show only a shallow depression for this diverticulum.

Primitive theropods lack recesses on the lateral surface of the basipterygoid processes *Coelophysis rhodesiensis* (Raath, 1977). Invasive basipterygoid sinuses occur in *Piatnitzkysaurus floresi* (Bonaparte, 1986), but are absent in *Acrocanthosaurus* (OMNH 10146, NCSM 14345) and *Sinraptor dongi* (IVPP V10600). Invasive basipterygoid processes arise independently in *Itemirus medullaris* (Kurzanov, 1976) and the tyrannosauroid *S. clevelandi* (UMNH VP 7818).

Sereno et al. (1996, 1994) list the exclusion of the basisphenoid from the basal tubera as a synapomorphy of Allosauroidea. However, the basisphenoid does form a small part of the basal tubera in *Acrocanthosaurus atokensis* (OMNH 10146, NCSM 14345), *Monolophosaurus jiangi* (Zhao & Currie, 1993), but does not contribute to the basitubera in *Sinraptor dongi* (IVPP V10600). The exclusion of the basisphenoid from the basal tubera and the groove between the basisphenoid and the basal tubera are shared between *Sinraptor* and *Allosaurus*.

**Basioccipital**—The basioccipital (Figs. 7–10) forms most of the occipital condyle but is excluded from the margin of the foramen magnum by the otoccipitals. The occipital condyle is shield-shaped in outline in posterior view. The neck of the condyle is robust and not constricted. It forms the medial walls of the paracondylar recesses. The posterior face of the basioccipital is gently concave transversely and its ventral margin is strongly concave. The ventral portion of the basioccipital intervenes between the posterolateral portions of the basisphenoid and exclusively forms the paired basal tubera.

The basioccipital in *Allosaurus jimmadseni* (DINO 11541, MOR 693, BYU 17672) is similar to that of *Allosaurus fragilis* (DINO 2560, UMNH VP 16605) in shape and in its minor contribution to the foramen magnum, similar to the condition in *Acrocanthosaurus atokensis* (NCSM 14345). In *Sinraptor dongi* (IVPP V10600) the basioccipital is excluded from the foramen magnum.

**Otoccipital (Exoccipital-Opisthotic)**—The exoccipital (Figs. 5–7, 9 and 10) is fused with the opisthotic in *Allosaurus jimmadseni* as in all dinosaurs and will be further referred to as the otoccipital sensu Sampson & Witmer (2007). The otoccipitals forms nearly the entire margin of the foramen magnum, except for a tiny contribution from the supraoccipital dorsally and the basioccipital ventrally. Dorsolateral to the foramen magnum on each side there is an elliptical depression for the insertion of *M. rectus capitus posterior*. The suture between otoccipital and the basioccipital runs through the floor of the paracondylar recess. The opisthotic portion of the fused exoccipital-opisthotic form the prominent paroccipital processes that are ventrolaterally and posterolaterally directed on either side of the foramen magnum and are also visible in lateral view. The medioventral margin of the paroccipital process extends ventrally, lateral to the basioccipital and occipital condyle, to almost the level of the basal tubera. Both the medial and lateral ventral distal ends of the paroccipital processes terminate well below the level of the occipital condyle. The paroccipital processes neither taper nor expand distally. The posterior face of the paroccipital process has an elongate depression for the *M. obliquus capitis magnus* (Raath, 1977). The paroccipital recess includes foramina for cranial nerves XI and XII. The anterior face of the paroccipital process has a depression, which is part of the dorsal tympanic recess. The otoccipital forms the medial wall of the fenestra ovalis.

The paroccipital processes of *Monolophosaurus* (Zhao & Currie, 1993) and *Sinraptor dongi* (Currie & Zhao, 1993a) are directed posteroventrallly and laterally, although not to the degree seen in *Allosaurus*. They are long, but laterally directed in *Acrocanthosaurus* (cast OMNH 10146), which is the primitive condition for theropods. The paroccipital processes are unknown or unreported in other allosauroids.

**Supraoccipital**—The supraoccipital (Figs. 6 and 10) is shaped like an inverted “T” in posterior view. Its dorsal ramus forms the massive triangular postnuchal sagittal crest at the rear of the skull. It is triangular in cross-section in dorsal view. The supraoccipital is posterior to the parietal which surrounds it on all of its dorsal and lateral contacts. Ventrally, the supraoccipital contacts the dorsomedial surfaces of the otoccipitals. Dorsally the supraoccipital contributes a tiny portion to the dorsal margin of the foramen magnum in *Allosaurus jimmadseni* (DINO 11541, MOR 693 and SMA 00005). The postnuchal supraoccipital crest is similar to *Allosaurus fragilis* in that; it is nearly the height of the parietal nuchal crest, it is more developed dorsally, it becomes lower and narrower ventrally, and it has a concave dorsal surface. The ventrolateral parts of the supraoccipital are short blunt arms in contact with the parietals dorsally and otoccipitals ventrally. The supraoccipital does not contact the squamosal. The postnuchal crest is likely the origin of the *Ligamentum nuchae*. The narrow flat area lateral to the nuchal crest may have been for the origin of the *M. spinalis capitis* (Raath, 1977).

There is a pair of openings on each side of the supraoccipital, dorsolateral to the foramen magnum. The more medial pair is wholly within the supraoccipital, the lateral one lies along the contact with the parietal. Similar openings have been described in *Tyrannosaurus rex* (Osborn, 1912) and *Troodon formosus* (Currie & Zhao, 1993b). In both cases the lateral opening is identified as a remanent of the posttemporal fenestra, and a similar feature is present in *Herrerasaurus ischigualastensis* (Sereno & Novas, 1993). The medial opening has been identified as a venous foramen in *Tyrannosaurus rex* (Osborn, 1912), and for the posterior canal of the middle cerebral vein in *Troodon formosus*. Both openings are present in *Allosaurus jimmadseni* and *Allosaurus fragilis*. As in *Troodon formosus*, these openings are connected by a well-marked groove in *Allosaurus*. A similar condition appears to be present in *Monolophosaurus jiangi* (Zhao & Currie, 1993). Both openings are present in *Acrocanthosaurus*, although they are not connected by a groove (OMNH 10146). In *Sinraptor dongi*, only the opening for the posterior canal of the middle cerebral vein is present (Currie & Zhao, 1993a).

The supraoccipital does contribute to the dorsal margin of the foramen magnum in *Allosaurus fragilis* (DINO 2560, UMNH VP 5472, UMNH VP 7415, UMNH VP 16652). The supraoccipital contributes a small area along the dorsal surface of the foramen magnum in *Acrocanthosaurus atokensis* (Eddy & Clarke, 2011) and *Giganotosaurus carolinii* (MUCPv-CH-1) (Coria & Currie, 2002).

### Bones of the splanchnocranium

**Ceratobranchial**—Both hyoids of *Allosaurus jimmadseni* (Fig. 13) are preserved in DINO 11541. The elements were in close articulation with each other with the anterior ends separated by only two cm. Presumably, the corpus was present originally but was either taphonomically removed or was cartilaginous. The hyoid is a slightly curved rod with an expanded anterior end that terminates in a flat articular surface where they would have met the corpus along the midline. The hyoids were slightly displaced with the anterior ends located just posterioventral to the last dentary tooth and the posterior ends oriented above the nuchal crest medial to the preserved left side of the skull. The posterior ends taper to a point.

Hyoids are rarely preserved in theropods but are known from the neotype of *Allosaurus fragilis* USNM 4734 (Carrano, Loewen & Evers, 2018), these elements are identical in form to *Allosaurus jimmadseni*. The hyoids of *Coelophysis kayentakatae* (Rowe, 1989) are cylindrical rods similar to that of *Allosaurus jimmadseni* but lack the modification of the proximal articular ends. The hyoids of *Carnatorus sastrei* (Bonaparte, Novas & Coria, 1990) are similar in shape to those of *Allosaurus jimmadseni*, but are much more robust. The hypoid of *Allosaurus jimmadseni* differs in its flat articular surfaces on the anterior end when compared to horizontal flange in *Sinraptor dongi* (Currie & Zhao, 1993a). We interpret the tapered end of the hyoid of *Sinraptor dongi* (Currie & Zhao, 1993a) as the posterior end of the element.

**Stapes**—Both stapes are present, but slightly displaced in DINO 11541 and in MOR 693 (Figs. 7–8 and 10). The left stapes is a long thin bone (59 mm long, two mm wide) with a slightly expanded distal end three mm wide. Much of its surface was damaged during preparation. It is visible only in medial view of the skull. The stapes has slipped ventrally from its life position and most of it is ventral to the left paroccipital process, although the proximal end is hidden beneath (anterior to) the proximal end of the paroccipital process. The footplate is not visible. Both stapes are present but displaced in MOR 693.

The right stapes of DINO 11541 is displaced and fractured in two pieces. The proximal end is lateral and ventral to the left basisphenoid. It is 14 mm long and the footplate is not visible. The other part is medial and ventral to the left pterygoquadrate flange. It measures 31 mm in length and has a slightly expanded end measuring three mm. The stapes was originally complete, but the middle section was lost during preparation. If this section was present, the exposed part of the stapes would measure at least 63 mm.

The stapes is known in *Allosaurus fragilis* (UMNH VP 16605, 16606) although the element is complete only in UMNH VP 16606 (UUVP 5961) (Madsen, 1976, figs. 9a and 17). As in that specimen, there is no evidence of an ossified extracolumella. Only the bases of both stapes are preserved in *Sinraptor dongi* (Currie & Zhao, 1993a).

**Epipterygoids**—The left epipterygoid of *Allosaurus jimmadseni* (Fig. 5) is preserved in MOR 693 and overlaps the lateral surface of the quadrate flange of the pterygoid. It is triangular and elongated with the apex of the element contacting the anterior surface of the laterosphenoid dorsally with a rounded bulbous condyle. The ventral surface is longer posteriorly than anteriorly. There is a pronounced ridge along the posterolateral surface.

The epipterygoid of *Allosaurus fragilis* forms an elongate triangular with a rounded dorsal laterosphenoid condyle and a flattened ventral surface. The epipterygoid of *Allosaurus fragilis* is wider ventrally than in *Allosaurus jimmadseni*. The rounded condyle for articulation with the laterosphenoid in both *Allosaurus fragilis* (UMNH VP 5326, BYU 8901) and *Allosaurus jimmadseni* (MOR693) differ from *Acrocanthosaurus atokensis* (NCSM 14345) and *Cryolophosaurus ellioti* (FMNH PR1821) a which have more elongated triangular shapes and pointed dorsal ends. The posterolateral ridge in *Allosaurus jimmadseni* is similar to the condition in *Acrocanthosaurus atokensis* (NCSM 14345).

**Quadrates**—The dorsal end of the quadrate (Figs. 4–8 and 10) sits in the quadrate cotylus of the squamosal. In lateral view, the quadratojugal laps onto and obscures most of the lateral surface of the quadrate so that only a small part of the dorsal 1/3 of the dorsal ramus is visible posterior to the descending ventral ramus of the squamosal. In addition, a small part of the lateral distal condyle is visible laterally posteroventral to the posteroventral end of the quadratojugal. A large lateromedially-flattened pterygoid wing is visible in the laterotemporal fenestra when viewed laterally. The pterygoid wing of the quadrate extends anteriorly from the posteromedial quadrate. Anteriorly, the quadrate and pterygoid articulate along the anteroventral corner of the pterygoid flange of the quadrate. In medial view, the pterygoid flange is concave with a distinct fossa present. The distal end of the quadrate has two distinct condyles for articulation with the mandible. The lateral and medial condyles are elongated ovals with the long axis oriented at 40° from the sagittal plane. The medial condyles are smaller than the lateral condyles. The medial condyle fits into the cotylus of the articular and the lateral condyle fits into the cotylus formed by the surangular and articular. Posteriorly, there is an oval quadrate foramen at nearly midheight. This foramen is almost completely composed of the quadrate but the lateralmost edge (about 15%) is boardered by the quadratojugal.

The quadrate of *Allosaurus jimmadseni*, similar to *Allosaurus fragilis*, is relatively short compared to most theropods, and similar in height to the jugal. In contrast, *Ceratosaurus nasicornis* (MWC 1) and *Acrocanthosaurus atokensis* (Currie & Carpenter, 2000) have a quadrate that is nearly as tall as the entire posterior portion of the skull. The postion of the posterior quadrate foramen in *Allosaurus jimmadseni* is similar to that of *Allosaurus fragilis* and differs from *Acrocanthosaurus* (NCSM 14345) and *Sinraptor dongi* (IVPP 10600) in its relatively high position and large size.

**Articulars**—The articular (Figs. 4–8 and 10) forms part of the articular surface for the quadrate. Because the left hemimandible is still occluded, the dorsal surface of the mandibular glenoid is not visible in DINO ll541. The articular articulates with the prearticular, surangular, and the antarticular. In medial view, the articular posterior to the mandibular glenoid is widened medially and the dorsal surface of this widened area is deeply concave. This concavity slopes medioventrally and the median margin of the articular is rounded and thickened. Crocodilians have a similar morphology, although in that group the retroarticular process is longer and the dorsal surface is not nearly as deeply excavated. Based on comparison with crocodilians (Iordansky, 1973; Schumacher, 1973) the deep concavity in *Allosaurus jimmadseni* is for the insertion of the *M. depressor mandibulae* and the thickened medial edge of the process marks the insertion of retroarticular aponeurosis of the *M. pterygoideus posterior*. Sereno et al. (1996) listed this pendant medial process of the articular as a synapomophy of Allosauroidea. The anterior face of the medial process is pierced by the foramen for the chorda tympani nerve and posterior condylar artery (Currie & Zhao, 1993a; Heaton, 1979). Only a small part of the articular is visible in lateral view.

In *Allosaurus fragilis* (DINO 2560) a high, vertical, transverse wall forms the posterior margin of the insertion for the *M. depressor mandibulae*. Posterior to this the posterior face of the articular is concave and slopes lateroventrally. This area may also mark the insertion for the *M. pterygoideus posterior*. This is also present in *Allosaurus jimmadseni*.

The concavity immediately posterior to the mandibular glenoid is absent in *Monolophosaurus jiangi* (Zhao & Currie, 1993). It is present in *Sinraptor dongi* (Currie & Zhao, 1993a), *Sinraptor hepingensis* (Gao, 1992), *Yangchuanosaurus shangyuensis*, and *Yangchuanosaurus shangyouensis* (Dong, Zhou & Zhang, 1983). It is present in *Acrocanthosaurus atokensis* (Eddy & Clarke, 2011; Harris, 1998), but the concavity is much narrower anteroposteriorly.

**Antarticulars**—The antarticular (Figs. 6, 8, 10 and 14) is an element near the articular surfaces of the posterior mandible and amongst tetanurans has only been recognized in *Allosaurus*. It was first recognized by Madsen (1976) in *Allosaurus fragilis*. It has subsequently been described in the possible tyrannosauroid *Bagaraatan ostromi* (Osmolska, 1996), but if in this specimen the antarticular is present, it is a thin spline that sits on the prearticular, articular and surangular anteromedial to the medial glenoid socket. In *Allosaurus* the antarticular is a sub-pyramidal element that sits in a pocket formed by the surangular, prearticular, and articular on the posteromedial surface of the mandible. It is present in *Allosaurus jimmadseni* on both mandibles of MOR 693; however, it is easily lost. Among skulls of *Allosaurus fragilis* it is present only in completely articulated skulls, including DINO 2560. In the Cleveland-Lloyd Dinosaur Quarry the antarticular is found only as an isolated element and is not known in-place from any jaw material. Because of its loose connection with the rest of the mandible and the fact that the right side of the skull of DINO 11541 was removed by water action, there is a high probability that the antarticular in this specimen was lost, especially as it is similar in size to some of the clasts in the matrix.

### Dermal bones of the lower jaw

**Surangulars**—The surangular (Figs. 4–8 and 10) is relatively dorsoventrally deep in lateral view, reflecting a relatively small external mandibular fenestra. Anterodorsally, there is a groove on the lateral surface of the surangular which broadens anteriorly. Madsen (1976) illustrates a foramen in the groove in *Allosaurus fragilis*, but a foramen is lacking in *Allosaurus jimmadseni*. In DINO 11541, dorsal to the external mandibular fenestra, the surangular has many large shallow pits. When prepared, these pits were filled with sand and so are not pebble impressions. The pits may be pathological. Laterally, the surangular ridge is well developed (Figs. 4 and 5). There is a tiny surangular foramen beneath this ridge at midlength as in *Allosaurus fragilis* (Madsen, 1976). The retroarticular process in *Allosaurus jimmadseni* is shorter posteriorly than in *Allosaurus fragilis* (Madsen, 1976). The surangular overlaps the angular for most of the retroarticular process, although the angular is more exposed in lateral view than in *Allosaurus fragilis* (Madsen, 1976). The surangular has only a small medial exposure, dorsal to the coronoid.

A pronounced surangular ridge is present in all allosauroids. No surangular foramen is reported in *Cryolophosaurus* (Hammer & Hickerson, 1994); however, one is located beneath the surangular ridge in *Allosaurus fragilis*, *Allosaurus jimmadseni, Monolophosaurus* (Zhao & Currie, 1993), and *Acrocanthosaurus atokensis* (Eddy & Clarke, 2011). Although Stovall & Langston (1950) indicate that this foramen is large in *Acrocanthosaurus* relative to other allosauroids, first-hand examination of NCSN 14345 indicates that the surangular foramen is not significantly larger in *Acrocanthosaurus atokensis*. In *Sinraptor dongi* there is a second surangular foramen that is ventral to the mandibular glenoid (Currie & Zhao, 1993a).

*Cryolophosaurus* (Hammer & Hickerson, 1994; Smith et al., 2007) and *Monolophosaurus* (Zhao & Currie, 1993; Brusatte, Benson & Xu, 2010) are primitive in lacking a retroarticular process. The process is more developed in *Sinraptor dongi* (Currie & Zhao, 1993a), *Yangchuanosaurus shangyouensis* (Dong, Zhou & Zhang, 1983), and *Acrocanthosaurus atokensis* (38); these exhibit a squared off end with a short retroarticular process. *Sinraptor hepingensis* (Gao, 1992) and *Yangchuanosaurus shangyuensis* (Dong, Zhou & Zhang, 1983) appear to have a well-developed retroarticular process, reminiscent of the condition in *Allosaurus*.

Primitively, theropods have a relatively large external mandibular fenestra, and as a result, the portion of the surangular anterior to this fenestra is shallow (*Coelophysis rhodesiensis* (Raath, 1977), *Coelophysis kayentakatae* (Rowe, 1989), *Coelophysis bauri* (Colbert, 1989)) Molnar, Dong & Kurzanov (1990) noted that some derived theropods (their Carnosauria) have a shallow external mandibular fenestra and consequently the anterior portion of the surangular is deep. Sereno et al. (1996) suggested this was a synapomorphy for Avetheropoda. Harris (1998) redefined the character and divided it in two, because of variation in the size of the external mandibular fenestra in allosauroids. Among allosauroids a shallow external mandibular fenestra and deep surangular occurs in *Acrocanthosaurus atokensis* (Eddy & Clarke, 2011), *Allosaurus fragilis*, *Allosaurus jimmadseni*, and *Monolophosaurus jiangi* (Zhao & Currie, 1993). The primitive condition is retained in *Sinraptor dongi* (Currie & Zhao, 1993a), *Sinraptor hepingensis* (Gao, 1992), *Yangchuanosaurus shangyuensis*, and *Yangchuanosaurus shangyouensis* (Dong, Zhou & Zhang, 1983). The condition is unknown or unreported in other allosauroids. The derived condition arises independently in tyrannosaurids (Molnar, Dong & Kurzanov, 1990). Madsen (1976) shows *Allosaurus fragilis* as having a very short and shallow external mandibular fenestra. However, the specimen upon which that composite restoration is based on, DINO 2560, has a long, shallow external mandibular fenestra on both hemimandibles. We are unaware of any specimens of *Allosaurus fragilis* that match the condition illustrated in Madsen’s (1976) monograph.

**Prearticulars**—The prearticular (Figs. 4–8 and 10) is a broadly U-shaped element that contacts the dentary, coronoid, splenial, surangular, antarticular, and articular. Its anterior process is relatively deep; it is shallowest at midlength and becomes slightly deeper posteriorly. Dorsally, it forms the ventral and the majority of the anterior margin of the adductor fossa. Along the anteroventral margin there is a large notch, the posterior mylohyoid foramen (Madsen, 1976) for the anterior, medial, and posterior branches of the mandibular ramus of the trigeminal nerve (Schumacher, 1973). The lateroventral margin of the prearticular fits into a groove on the medioventral surface of the angular, forming a tight articulation. The posterior end is blunt and does not reach the end of the mandible. The mandibular joint runs between the prearticular and the dentary and splenial.

Overall, the prearticular of *Allosaurus* is similar in shape to that in other large theropods.

The prearticular has a very narrow exposure in lateral view through the external mandibular fenestra in *Allosaurus*, *Acrocanthosaurus atokensis* (Eddy & Clarke, 2011), *Sinraptor dongi* (Currie & Zhao, 1993a), and *Yangchuanosaurus shangyouensis* (Dong, Zhou & Zhang, 1983).

**Angulars**—The angular (Figs. 4, 5, 8 and 10) is visible mainly in lateral view (it is partially visible in ventral and posterior views). It contacts the dentary anteriorly, lapping medially to the posteroventral process of the dentary in a lap joint. The angular includes an elongate socket medially into which the prearticular sits and the two bones form the posteroventral surface of the mandible. The contact with the surangular is slightly more complex, most of the dorsal surface of the angular seems meets the ventral surface of the surangular in a butt joint and the posterior end of the angular laps onto the posteroventral surface of the prearticular, surangular and articular. Laterally, the angular extends as a thin process all the way to the posterior end of the jaw.

Although Madsen (1976) shows the angular ending short of the posterior limit of the mandible in *Allosaurus fragilis*, it does reach the end in USNM 4734, DINO 2560, BYU 9466 and all other known articulated specimens. We recognize this condition as typical for *Allosaurus* as the condition is also present in *Allosaurus europaeus*. Among tetanurans, the angular nearly reaches the posterior end of the jaw in *Cryolophosaurus* (Smith et al., 2007). It does not reach the end of the mandible in *Acrocanthosaurus atokensis* (Eddy & Clarke, 2011), *Monolophosaurus jiangi* (Zhao & Currie, 1993; Brusatte, Benson & Xu, 2010), *Sinraptor dongi* (Currie & Zhao, 1993a), *Sinraptor hepingensis* (Gao, 1992), *Yangchuanosaurus shangyuensis*, and *Yangchuanosaurus shangyouensis* (Dong, Zhou & Zhang, 1983).

**Coronoids**—The coronoid is visible in medial view along the medial surface of the mandible (Figs. 7 and 8). It is a thin triangular bone forming the rostrodorsal margin of the adductor fossa. Dorsally, the coronoid contacts the surangular. Ventrally, it overlaps the prearticular and anteriorly is overlapped medially by the prearticular. Among allosauroids, the coronoid is well-known *Allosaurus fragilis* and *Monolophosaurus* (Zhao & Currie, 1993). It is present but broken in *Acrocanthosaurus atokensis* (Eddy & Clarke, 2011). The only significant difference among these taxa is that in *Monolophosaurus* there is a neurovascular canal on the anterior half of the coronoid.

**Splenials**—The splenial is a roughly triangular bone that forms a lateral wall covering the posterior half of the meckelian canal (Figs. 7 and 8). It does not wrap around the ventral margin of the dentary. The anterior end of the splenial is forked. The anterior mylohyoid foramen is located at about midlength along the ventral margin. In *Allosaurus jimmadseni* the ventral margin of the mylohyoid foramen is nearly completely closed by a thin process of the splenial. In *Allosaurus fragilis* the foramen is more like a notch and opens ventrally (Madsen, 1976). In *Sinraptor dongi* the foramen is completely within the splenial (Currie & Zhao, 1993a). The condition in *Monolophosaurus jiangi* (Zhao & Currie, 1993) is like that in *Allosaurus jimmadseni*. The posterior margin of the splenial is bifurcated, forming the anterodorsal sweep of the prearticular. The ventral ramus is much longer than the dorsal process. Posteriorly, the splenial is separated from the prearticular by the intramandibular joint.

**Supradentaries**—The supradentary is a thin strap-shaped bone which lies along the mediodorsal margin of the dentary and lies lateral to the base of the teeth (Figs. 7 and 8). It runs from the third dentary tooth to the last dentary tooth. Its posteroventral margin is overlapped by the splenial. This element is preserved in both DINO 11541 and MOR 693.

**Dentaries**—The dentaries (Figs. 4, 5, 7 and 8) taper posteriorly in lateral view, contacting the surangular to the dorsal surface of the external mandibular fenestra. There is a posteriorly concave notch that forms the anterodorsal portion of the external mandibular fenestra, producing a forked posteroverntral ramus. The posteroventral ramus of the dentary laps lateral to the angular along a short ramus ventral to the anterior margin of the external mandibular fenestra. Mental foramina, possibly for innervation of the skin by the inferior alveolar nerve (Currie & Zhao, 1993a) are present on the lateral surface of the dentary. These foramina are rounded on the anterior portion of the dentary and are horizontally elliptical posteriorly. Ventrolaterally, these foramina are isolated, as in *Monolophosaurus* (Zhao & Currie, 1993), *Neovenator* (Hutt, Martill & Barker, 1996; Brusatte, Benson & Hutt, 2008), and *Piatnitzkysaurus* (Bonaparte, 1986). Dorsolaterally, there is a distinct row of foramina about one tooth width below the dorsal surface of the dentary similar to the condition in *Acrocanthosaurus atokensis* (Eddy & Clarke, 2011), *Giganotosaurus* (Coria & Salgado, 1995) and *Sinraptor dongi* (Currie & Zhao, 1993a). The inter-dentary symphysis is vertical and smooth and likely allowed for movement between the right and left hemimandibles. Both DINO 11541 and MOR 693 exhibit disarticulation along this suture. There is very little medial curvature of the dentary in dorsal view, indicating a relatively narrow snout. The medial surface of the dentary is vertical and flat. The meckelian canal is positioned on the ventral third of the medial surface of the dentary, and the posterior part of the meckelian canal is covered by the splenial. However, the anterior part of this canal is visible and it runs nearly to the symphysis. A foramen is present ventral to the anterior end of the meckelian canal and was probably for the symphiseal ramus of the inferior alveolar nerve (Currie & Zhao, 1993a). A small section of the posterior margin of the dentary is visible between the splenial and prearticular.

### Dentition

**Premaxillary dentition**—There are five premaxillary teeth in each premaxilla of *Allosaurus jimmadseni*. The typical premaxillary tooth count in theropods is four, although *Ceratosaurus* has three (Gilmore, 1920). Among basal tetanurans five premaxillary teeth have been reported only in *Allosaurus fragilis*, *Allosaurus jimmadseni*, and *Neovenator* (Hutt, Martill & Barker, 1996; Brusatte, Benson & Hutt, 2008); however, tooth counts higher than four have arisen independently at least two other times in the Theropoda. The basal theropod *Sinosaurus triassicus* (previously *Dilophosaurus sinensis*) (Hu, 1993) has five premaxillary teeth. Spinosaurids, have a premaxillary tooth count of seven, arranged in a terminal rosette.

**Maxillary dentition**—There are 16 maxillary teeth in DINO 11541 and on either side of MOR 693. The anterior maxillary teeth are vertical, but angle backward with posterior progress along the tooth row. The teeth are anteroposteriorly broadest at the midlength of the tooth row. The maxillary teeth are typical of *Allosaurus*; laterally compressed, with a relatively straight posterior margin and recurved anterior margin. Serrations are small, flat topped, and extend further down posterior margin of tooth than the anterior end.

**Dentary dentition**—There are 19 dentary teeth in DINO 11541 and 18 and 19 in MOR 693. This is the highest maxillary tooth count known for any allosauroid. *Allosaurus fragilis* has either 16 or 17 dentary teeth in the dentary. They are labiolingually compressed and with a recurved crown. Numbers 1 and 9 are incoming replacement teeth, but all the others are fully erupted. The roots are exposed on most teeth, but especially so on the first eight, because of the shallow depth of the medial face of the dentary. Serrations are present on both the anterior and posterior edges of the teeth.

The teeth of *Allosaurus jimmadseni* do not differ morphologically from those of *Allosaurus fragilis* and most other allosauroids or large theropods. *Carcharodontosaurus* has strikingly different maxillary teeth in that they are symmetrical, rather than recurved, and have marked curved enamel wrinkles, one per denticle, with some extending across the labial and lingual surfaces (Sereno et al., 1996). Similar wrinkles are present in the dentary teeth of *Giganotosaurus carolinii*, but not in *Acrocanthosaurus atokensis* or *Saurophaganax maximus*.

### Atlas–Axis complex

**Atlas**—The atlantal pleurocentrum, atlantal intercentrum, and atlantal neural arches of *Allosaurus jimmadseni* are preserved in MOR 693 and SMA 0005 (Fig. 15). They are similar to the atlantal complex of *Allosaurus fragilis* (USMN 4734, UMNH VP 9089). The pleurocentrum of the atlas is semi-crescentic in anterior view with a concave dorsal surface and convex ventral surface. The anterior surface is slightly concave as well, and forms the ventral portion of the articular surface for the occipital condyle along with the anterior portions of the atlantal intercentrum and the anterodorsal portion of the anterior surface of the axial intercentrum. In lateral view, the atlantal plerurocentrum is sub-rectangular and only about 70% as long anteroposteriorly as the axial intercentrum and only about as tall dorsoventrally as the atlantal intercentrum. The position of the atlantal pleurocentrum is more ventral than the axial intercentrum. The atlantal intercentra are unfused and articulate with the atlantal pleurocentrum ventrally and do not touch each other dorsomedially. They make up the dorsalmost 30% of the lateral part of the articular facet for the occipital condyle. The atlantal neural arches sit dorsally on the atlantal intercentra and articulate with each other anterodorsomedially. In dorsal view, the articulated neural arches have lateral, posteriorly directed transverse processes that form a “V” shaped chevron that points anteriorly and sweeps posteriorly around the neural arch of the axis.

The ventral position of the atlantal pleurocentrum in *Allosaurus jimmadseni* contrasts with the more dorsal position of the atlas in *Allosaurus fragilis* (UMNH VP 9081). The atlas is more rectangular in lateral view and more rounded in anterior view in *Allosaurus jimmadseni* than in *Sinraptor dongi* (IVPP 10600).

**Axis**—The axial intercentrum, pleurocentrum, and neural arch of *Allosaurus jimmadseni* (Fig. 15) are preserved in DINO 11541, MOR 693, SMA 0005 and BYU 10599. The intercentrum is firmly attached to the centrum although the suture is still clearly visible. The intercentrum is circular in anterior view and its anterior margin is flared. The axial intercentrum is rotated dorsally such that its anterior surface faces anterodorsally in SMA 0005, DINO 11541 and BYU 10599 but is more ventrally oriented in MOR 693. As a result, the ventral surface of the intercentrum is not in line with the ventral surface of the axial centrum, except in MOR 693. The ventral orientation of the ventral surface of the axial intercentrum (more in line with the ventral surface of the axial pleurocentrum) in MOR 693 is more like the condition in *Allosaurus fragilis* in USMN 4734, UMNH VP 9089, and BYU 8901. This rotated intercentrum brings the vertebral column up under occipital condyle and supports the skull in a way unusual for Jurassic theropods (Currie & Zhao, 1993a). This condition is also present in *Sinraptor dongi* (Currie & Zhao, 1993a), *Monolophosaurus jiangi* (Zhao & Currie, 1993), *Yangchuanosaurus shangyouensis* (Dong, Zhou & Zhang, 1983) and *Marshosaurus bicentesimus* (CMNH 217040).

The ventral portion of the axial pleurocentrum is pinched but does not form a distinct keel. The axial centrum is more mediolaterally compressed in *Allosaurus jimmadseni* (DINO 11541, MOR 693, SMA 0005, BYU 10599) than in *Allosaurus fragilis* (USMN 4734, UMNH VP 9089, BYU 8901). An elliptical central foramen is present ventral to the diapophysis and posterior to the parapophysis, showing that the axis is pneumatic. The long axis of the foramen is inclined about 30° to the long axis of the centrum the orientation of this foramen is like that in *Allosaurus fragilis* (Gilmore, 1920) and *Acrocanthosaurus atokensis* (Harris, 1998) and unlike that of *Sinraptor dongi* and *Monolophosaurus* where the foramen is vertical (Zhao & Currie, 1993; Currie & Zhao, 1993a). This foramen is absent in *Piatnitzkysaurus* (Bonaparte, 1986) and appears to be missing in *Yangchuanosaurus shangyouensis* as well (Dong, Zhou & Zhang, 1983).

The odontoid process is not fused to either the centrum or intercentrum. In anterior view, it is tall and narrow, rather than reniform as in *Allosaurus fragilis, Yangchuanosaurus shangyouensis*, and *Piatnitzkysaurus floresi*, and *Acrocanthosaurus atokensis* (Madsen, 1976; Bonaparte, 1986; Harris, 1998; Dong, Zhou & Zhang, 1983). In contrast to *Acrocanthosaurus atokensis* the odontoid process in *Allosaurus* is apneumatic (Britt, 1993).

The axial diapophysis is small and pendant as is in most large Jurassic theropods, with the exception of *Piatnitzkysaurus* (Bonaparte, 1986) where it is poorly developed and more posteriorly positioned. A pneumatic fossa is present beneath the posterior margin of the base of the diapophyses. A similarly placed fossa is present in *Allosaurus fragilis*, *Sinraptor dongi* (Currie & Zhao, 1993a), and *Yangchuanosaurus shangyouensis* but absent in *Piatnitzkysaurus* (Bonaparte, 1986). In *Acrocanthosaurus atokensis* a pneumatic foramen is present (Harris, 1998). The condition is not described in *Monolophosaurus* (Zhao & Currie, 1993).

The neuropophysis is well developed. The neural spine slants craniodorsally and a large notch separates the spine from the postzygopophyses. The epipophyses are small as in *Allosaurus fragilis* and *Monolophosaurus* (Madsen, 1976; Zhao & Currie, 1993) and only extend to the posterior end of the postzygapophysis. Axial epipophyses are larger in *Piatnitzkysaurus*, *Sinraptor*, and *Szechuanoraptor* (Bonaparte, 1986; Currie & Zhao, 1993a; Dong, Zhou & Zhang, 1983).

The relative height of the neural spine compares closely with *Allosaurus fragilis*. There is less separation between the axial epipophyses and neural spine in *Sinraptor dongi* (Currie & Zhao, 1993a), *Yangchuanosaurus shangyuensis* (Dong, Zhou & Zhang, 1983) than in *Allosaurus*.

## Ontogenetic Assessment of DINO 11541

DINO 11541 is one of the most complete single theropod skeletons known from Late Jurassic formations anywhere in the world. The remarkable half-skull allows for an understanding of skull morphology that is rarely afforded any theropod. For these and other reasons, *Allosaurus jimmadseni* will be an important comparative specimen for future studies of theropods anatomy and evolution.

DINO 11541 is clearly a specimen of *Allosaurus*. Some of the features in the field appeared to be markedly different from *Allosaurus* (such as the thin and drawn out lateral edge of the foot of the right pubis), surprisingly, turned out to be asymmetries within the specimen. Nevertheless, as given in the diagnosis, there are several features which separate this specimen from all *Allosaurus fragilis* specimens and warrants formal systematic recognition.

Brochu (1996), however, provides another approach to this question. Neurocentral sutures close late in post-hatching ontogeny in crocodilians, and this closure proceeds from posterior to anterior in the presacral series. Thus, in crocodilians, the closure of the neurocentral sutures in the anterior end of the presacral column occurs late in ontogeny and indicates the attainment of morphological maturity (Brochu, 1996, 1992a, 1992b). While this approach cannot give an absolute age for an individual it does provide a size-independent criterion for maturity.

The holotype of *Allosaurus jimmadseni* has an estimated length of 5.6 m, assuming that the missing midcaudals come to about one m. It shows a number of features which suggest that it is not morphologically mature. The sacral centra are not fused and there is separation between the centra of sacrals 1 and 2. More relevant to Brochu’s (1996) indicators, the neurocentral suture is open throughout the entire presacral column, and there is slight separation along the neurocentral suture in cervicals 2 through 4. This suggests that the holotype of *Allosaurus jimmadseni* was relatively immature, and may not have undergone all ontogenetic changes, and was certainly not near its maximum size.

DINO 11541 does, however, show a pattern of neurocentral fusion in the caudal vertebrae which is in contrast with that of crocodilians. Brochu (1996) reports that in crocodilians the neurocentral suture is fully closed in most caudals at the time of hatching. In DINO 15541, the neurocentral suture is clearly open, as evidenced by a sediment filled gap between the neural arch and centrum in caudals 1 through 5 and caudal 8. In caudals 1 through 5 the neural arches, as a unit, pulled away from their arches for a distance up to three cm, indicating that there was stronger attachment between the neural arches than between each arch and its respective centrum. The larger size of MOR 693 and SMA 0005 further support the conclusion that *Allosaurus jimmadseni* was not near its maximum size.

As the lack of neurocentral fusion suggests that *Allosaurus jimmadseni* may not have undergone all ontogenetic changes, one might ask is the differences in *Allosaurus jimmadseni* might merely reflect this ontogenetic immaturity. Fortunately, the collections from the Cleveland-Lloyd Dinosaur Quarry can serve as an ontogenetic control, that is, when a feature appeared to be unique to *Allosaurus jimmadseni*, we examined specimens from Cleveland-Lloyd which were smaller, of equal size, and larger, in order to assess whether these differences might be ontogenetic in origin. The features diagnosing *Allosaurus jimmadseni* do not occur at any morphological stage represented by the Cleveland-Lloyd collection and are here interpreted to be of systematic significance.

## Conclusions

Based on all known data for specimens of *Allosaurus*, the genus contains two valid species from the Morrison Formation of North America, *Allosaurus fragilis* and *Allosaurus jimmadseni*, which are distinct from *Allosaurus europeaus* (Fig. 16). The jugal, maxilla and nasal of the two taxa differ in multiple characters, including features associated both with signaling structures (nasolacrimal crest in *Allosaurus jimmadseni*; lacrimal horn of *Allosaurus fragilis*) and with craniofacial modifications that more likely reflect modification under the direction of natural selection (e.g., transverse expansion of the rear portion of the skull in *Allosaurus fragilis*; dorsal displacement of the maxillary tooth row relative to the jaw joint in *Allosaurus fragilis*). Using these characters, this study assigns several specimens to *Allosaurus jimmadseni*. In a subsequent publication we will review all named species of *Allosaurus* from North America in support of our view that there are only two valid species of *Allosaurus* in North America, *Allosaurus fragilis* and *Allosaurus jimmadseni*.

## Supplemental Information

10.7717/peerj.7803/supp-1 Supplemental Information 1 Specimens and institutional locations.

## Institutional Abbreviations

AMNH: American Museum of Natural History, New York, New York, USA
BMNH: British Museum of Natural History, London, United Kingdom
BSP: Bayerische Staatsammlung für Paläontologie und historische Geologie, Munich, Germany
BYU: Earth Science Museum, Brigham Young University, Provo, Utah, USA
CC: Concordia College, Moorhead, Minnesota, USA
CEU: College of Eastern Utah, Price Utah, USA
CM: Carnegie Museum of Natural History, Pittsburg, Pennsylvania, USA
DINO: Dinosaur National Monument, Jensen, Utah, USA
DMNH: Denver Museum of Nature and Science (Formerly Denver Museum of Natural History), Denver, Colorado, USA
ELDM: Erlianhaote Dinosaur Museum, Inner Mongolia, China
FMNH: Field Museum of Natural History, Chicago, Illinois, USA
GR: Ruth Hall Museum of Paleontology, Ghost Ranch, Abiquiu, New Mexico, USA
IGM: Institute of Geology Mongolia, Ulaanbaatar, Mongolia
IPFUB: Institut für Geologische Wissenschaften der Freie Universität, Berlin, Germany
IVPP: Institute of Vertebrate Paleontology and Paleoanthropology, Beijing, China
LH: Museo de Cuenca, Cuenca, Spain
MACN: Museo Argentino de Ciencias Naturales, Buenos Aires, Argentina
MCZ: Museum of Comparative Zoology, Harvard University, Boston, Massachusetts, USA
ML: Museu da Lourinhã, Lourinhã, Portugal
MNA: Museum of Northern Arizona, Flagstaff, Arizona, USA
MNHM: Muséum national d’histoire naturelle, Paris, France
MOR: Museum of the Rockies, Bozeman, Montana, USA
MUCP: Museo de la Universidad Nacional del Comahue, Neuquén, Argintina
MWC: Museum of Western Colorado, Fruita, Colorado, USA
NCSM: North Carolina Museum of Natural Sciences, (formerly North Carolina State Museum), Raleigh, North Carolina, USA
NGMC: National Geological Museum of China, Beijing, China
NIGP: Nanjing Institute of Geology and Palaeontology, Nanjing, China
NMMNH: New Mexico Museum of Natural History and Science, Albuquerque, New Mexico, USA
NMV: National Museum of Victoria, Australia
OMNH: Sam Nobel Oklahoma Museum of Natural History, Norman, Oklahoma, USA
OUMNH: Oxford University Museum of Natural History, Oxford, United Kingdom
PALEON: Glenrock Paleontological Museum, Glenrock, Wyoming, USA
SC: Sheridan College, Sheridan, Wyoming, USA
PVL: Paleontología Vertebrados, Fundación Miguel Lillo, Tucumán, Argentina
ROM: Royal Ontario Museum, Toronto, Canada
SDSM: South Dakota School of Mines, Rapid City, South Dakota, USA
SGM: Ministere de l’Energie et des Mines Rabat, Morocco
SMA: Sauriermuseum of Aathal, Aathal, Switzerland
TPII: North American Museum of Ancient Life, Lehi, Utah, USA
UC: University of Chicago, Chicago Illinois, USA
UCMP: University of California Museum of Paleontology, Berkeley, California, USA
UMNH VP: Natural History Museum of Utah (Formerly the Utah Museum of Natural History and now the number for UUVP specimens), Salt Lake City, Utah, USA
UNSM: University of Nebraska State Museum, Lincoln, Nebraska, USA
USNM: National Museum of Natural History, (formerly United States National Museum), Smithsonian Institution, Washington, D.C., USA
UUVP: University of Utah vertebrate paleontology collection, Salt Lake City, Utah (now catalogued as UMNH VP)
UWGM: University of Wyoming Geologic Museum, Laramie, Wyoming, USA
WDS: Wyoming Dinosaur Center, Thermopolis, Wyoming, USA
YPM: Yale Peabody Museum, New Haven, Connecticut, USA
ZDCM: Zhucheng Dinosaur Museum, Shandong, China.

## Quarry Abbreviations

BAQ: (WY-61 of Turner & Peterson (1999)), Big Al Quarry, Big Horn County, Wyoming
BCQ: Bone Cabin Quarry, Albany County, Wyoming
CBQ: Como Bluff, Reed’s Quarries 1, 4, and 9, Alban County, Wyoming
DQ: Dana Quarry near Ten Sleep, Washakie County, Wyoming
FQ1: Marsh’s Felch Quarry 1, Garden Park, Colorado
CLDQ: Cleveland-Lloyd Dinosaur Quarry, Cleveland, Utah
DMQ: (CO-58 of Turner & Peterson (1999)), Dry Mesa Quarry, Colorado
DNMCQ: Carnegie Quarry. Dinosaur National Monument, Brushy Basin Member, Uinta County, Utah
DNMSW: (DNM-116 of Turner & Peterson (1999)), Dinosaur National Monument, Salt Wash Member, Uinta County, Utah
HKQ: Hinkle Quarry, Utah
HQ: (WY-62 of Turner & Peterson (1999)), Howe Quarry, Big Horn County, Wyoming
HSQ: Howe Stevens Quarry, Big Horn County, Wyoming
LHQ: (WY-11 of Turner & Peterson (1999)), Little Houston Quarry, Crook County, Wyoming
MC: Moffit County, Colorado
MQ: Meilyn Quarry, Carbon County, Wyoming.

## Additional Information and Declarations

### Data Availability

The following information was supplied regarding data availability: The raw data is in the article under descriptions and the specimens examined are available in the Supplemental File.

### New Species Registration

The following information was supplied regarding the registration of a newly described species: Publication LSID: urn:lsid:zoobank.org:pub:DF37FD14-171C-4C02-8A5B-D2FCE929AABF. Allosaurus jimmadseni Chure and Loewen sp. nov. urn:lsid:zoobank.org:act:4D577308-64BC-4F87-A1F6-EE0467CF1A2F.
