# Supplementary material for: Cranial anatomy of Allosaurus jimmadseni, a new species from the lower part of the Morrison Formation (Upper Jurassic) of Western North America
Source: PeerJ. 2020 Jan 24;8:e7803. doi: 10.7717/peerj.7803 (PMC6984342; doi:10.7717/peerj.7803)
Supplement: Supplemental Information 1 [file peerj-08-7803-s001.docx]

***Institutional Abbreviations***

**AMNH**, American Museum of Natural History, New York, New York, USA; **BMNH**, British Museum of Natural History, London, United Kingdom: **BSP**, Bayerische Staatsammlung für Paläontologie und historische Geologie, Munich, Germany; **BYU**, Earth Science Museum, Brigham Young University, Provo, Utah, USA; **CC**, Concordia College, Moorhead, Minnesota, USA; **CEU**, College of Eastern Utah, Price Utah, USA; **CM**, Carnegie Museum of Natural History, Pittsburg, Pennsylvania, USA; **DINO**, Dinosaur National Monument, Vernal, Utah, USA; **DMNH**, Denver Museum of Nature and Science (Formerly Denver Museum of Natural History), Denver, Colorado, USA; **ELDM**, Erlianhaote Dinosaur Museum, Inner Mongolia, China; **FMNH**, Field Museum of Natural History, Chicago, Illinois, USA; **GR**, Ruth Hall Museum of Paleontology, Ghost Ranch, Abiquiu, New Mexico, USA; **IGM**, Institute of Geology Mongolia, Ulaanbaatar, Mongolia; **IPFUB**, Institut für Geologische Wissenschaften der Freie Universität, Berlin, Germany; **IVPP**, Institute of Vertebrate Paleontology and Paleoanthropology, Beijing, China**; LH**, Museo de Cuenca, Cuenca, Spain; **MACN**, Museo Argentino de Ciencias Naturales, Buenos Aires, Argentina**, MCZ**, Museum of Comparative Zoology, Harvard University, Boston, Massachusetts, USA; **ML**, Museu da Lourinhã, Lourinhã, Portugal; **MNA**, Museum of Northern Arizona, Flagstaff, Arizona, USA; **MNHM**, Muséum national d'histoire naturelle, Paris, France; **MOR**, Museum of the Rockies, Bozeman, Montana, USA; **MUCP**, Museo de la Universidad Nacional del Comahue, Neuquén, Argintina; **MWC**, Museum of Western Colorado, Fruita, Colorado, USA; **NCSM**, North Carolina Museum of Natural Sciences, (formerly North Carolina State Museum), Raleigh, North Carolina, USA; **NGMC**, National Geological Museum of China, Beijing, China; **NIGP**, Nanjing Institute of Geology and Palaeontology, Nanjing, China; **NMMNH**, New Mexico Museum of Natural History and Science, Albuquerque, New Mexico, USA; **NMV**, National Museum of Victoria, Australia; **OMNH**, Sam Nobel Oklahoma Museum of Natural History, Norman, Oklahoma, USA; **OUMNH**, Oxford University Museum of Natural History, Oxford, United Kingdom; **PALEON**, Glenrock Paleontological Museum, Glenrock, Wyoming, USA; **SC**, Sheridan College, Sheridan, Wyoming, USA; **PVL**, Paleontología Vertebrados, Fundación Miguel Lillo, Tucumán, Argentina; **ROM**, Royal Ontario Museum, Toronto, Canada; **SDSM**, South Dakota School of Mines, Rapid City, South Dakota, USA; **SGM**, Ministere de l’Energie et des Mines Rabat, Morocco; **SMA**, Sauriermuseum of Aathal, Aathal, Switzerland; **TPII**, North American Museum of Ancient Life, Lehi, Utah, USA: **UC**, University of Chicago, Chicago Illinois, USA; **UCMP**, University of California Museum of Paleontology, Berkeley, California, USA; **UMNH VP**, Natural History Museum of Utah (Formerly the Utah Museum of Natural History and now the number for UUVP specimens), Salt Lake City, Utah, USA; **UNSM**, University of Nebraska State Museum, Lincoln, Nebraska, USA; **USNM**, National Museum of Natural History, (formerly United States National Museum), Smithsonian Institution, Washington, D.C. , USA; **UUVP**, University of Utah vertebrate paleontology collection, Salt Lake City, Utah (now catalogued as UMNH VP); **UWGM**, University of Wyoming Geologic Museum, Laramie, Wyoming, USA; **WDS,** Wyoming Dinosaur Center, Thermopolis, Wyoming, USA; **YPM**, Yale Peabody Museum, New Haven, Connecticut, USA; **ZDCM**, Zhucheng Dinosaur Museum, Shandong, China.

***Institutions Housing Specimens Discussed in the Paper***

**AMNH**, American Museum of Natural History, New York, New York, USA: AMNH 275; AMNH 287; AMNH 290; AMNH 324; AMNH 408; AMNH 496; AMNH 600; AMNH 619; AMNH 666; AMNH 680; AMNH 813; AMNH 851; AMNH 5750, AMNH 5753; AMNH 5767; AMNH 6125; AMNH 6128; AMNH 7223; AMNH 7224; AMNH 7239; AMNH 7241; and AMNH 7242

**BSP**, Bayerische Staatsammlung für Paläontologie und historische Geologie, Munich, Germany: BSP AS I 563

**BYU**, Earth Science Museum, Brigham Young University, Provo, Utah, USA: BYU 1059; BYU 2028; BYU 4861; BYU 5122; BYU 5164; BYU 5253; BYU 5268; BYU 5292; BYU 5583; BYU 8901; BYU 9466; BYU 12893 BYU 13024; BYU 13807; BYU 11936; BYU 13621; BYU 16942; BYU 17106; and BYU 17281

**CM**, Carnegie Museum of Natural History, Pittsburg, Pennsylvania, USA: CM 11844

**DINO**, Dinosaur National Monument, Vernal, Utah, USA: DINO 972; DINO 2560 (previously catalogued as UUVP 6000); DINO 3984; and DINO 11541

**DMNH**, Denver Museum of Nature and Science (Formerly Denver Museum of Natural History), Denver, Colorado, USA: DMNH 2419

**ELDM**, Erlianhaote Dinosaur Museum, Inner Mongolia; China: ELDM V1001

**FMNH**, Field Museum of Natural History, Chicago, Illinois, USA: FMNH P1505; FMNH PR1821; and FMNH P25114

**GR**, Ruth Hall Museum of Paleontology, Ghost Ranch, Abiquiu, New Mexico, USA: GR 241; GR 155; GR 242; GR 243; and GR 244

**IGM**, Institute of Geology Mongolia, Ulaanbaatar, Mongolia: IGM 100/29

**IPFUB**, Institut für Geologische Wissenschaften der Freie Universität, Berlin, Germany: IPFUB Gui Th 1; IPFUB Gui Th 2, and IPFUB Gui Th 3

**IVPP**, Institute of Vertebrate Paleontology and Paleoanthropology, Beijing, China: IVPP V2885; IVPP 10600; IVPP 84019; IVPP V11579; V14242; V14243; IVPP V14531; and V14532

**LH**, Museo de Cuenca, Cuenca, Spain: LH 7777

**MACN**, Museo Argentino de Ciencias Naturales, Buenos Aires, Argentina: MACN Pv CH895

**MCZ**, Museum of Comparative Zoology, Harvard University, Boston, Massachusetts, USA: MCZ 3897 R

**ML**, Museu da Lourinhã, Lourinhã, Portugal: ML 415

**MNA**, Museum of Northern Arizona, Flagstaff, Arizona, USA: MNA V3315

**MNHM**, Muséum national d'histoire naturelle, Paris, France; MNHN CNJ79; MNHN 1998-13

**MOR**, Museum of the Rockies, Bozeman, Montana, USA: MOR 693

**MUCP**, Museo de la Universidad Nacional del Comahue, Neuquén, Argintina; MUCPv-CH-1

**MWC**, Museum of Western Colorado, Fruita, Colorado, USA: MWC 1

**NCSM**, North Carolina Museum of Natural Sciences, (formerly North Carolina State Museum), Raleigh, North Carolina, USA: NCSM 14345

**NGMC**, National Geological Museum of China, Beijing, China: NGMC 97-4-002

**NIGP**, Nanjing Institute of Geology and Palaeontology, Nanjing, China: NIGP 127586; NIGP 127587

**OMNH**, Sam Nobel Oklahoma Museum of Natural History, Norman, Oklahoma, USA: OMNH 10146

**OUMNH**, Oxford University Museum of Natural History, Oxford, United Kingdom: OUMNH J.3311

**PVL**, Paleontología Vertebrados, Fundación Miguel Lillo, Tucumán, Argentina: PVL 4073

**ROM**, Royal Ontario Museum, Toronto, Canada: ROM 12868

**SDSM**, South Dakota School of Mines, Rapid City, South Dakota, USA: SDSM 30510

**SGM**, Ministere de l’Energie et des Mines Rabat, Morocco: SGM-Din 1

**SMA**, Sauriermuseum of Aathal, Aathal, Switzerland: SMA 0005

**TPII**, North American Museum of Ancient Life, Lehi, Utah, USA: TPII 2000-09-29

**UCMP**, University of California Museum of Paleontology, Berkeley, California, USA: UCMP 37302; UCMP 37303; and UCMP 77270

**UMNH VP**, Natural History Museum of Utah (Formerly the Utah Museum of Natural History and now the number for UUVP specimens), Salt Lake City, Utah, USA: UMNH VP 1251; UMNH VP 3113; UMNH VP 5278; UMNH VP 5316; UMNH VP 5326–5328; UMNH VP 5427; UMNH VP 5470; UMNH VP 5480; UMNH VP 6051; UMNH VP 6052; UMNH VP 6317; UMNH VP 6340; UMNH VP 6365; UMNH VP 6383; UMNH VP 6400; UMNH VP 6408; UMNH VP 6473; UMNH VP 6475; UMNH VP 6499; UMNH VP 6502; UMNH VP 7190; UMNH VP 7408; UMNH VP 7411; UMNH VP 7434; UMNH VP 7794; UMNH VP 7748; UMNH VP 7818; UMNH VP 7821; UMNH VP 7880; UMNH VP 7882, 7884–7885; UMNH VP 7889–7891; UMNH VP 7895; UMNH VP 7898; UMNH VP 7908; UMNH VP 7922; UMNH VP 7926–7930; UMNH VP 7932; UMNH VP 7934; UMNH VP 7937–7938; UMNH VP 7957; UMNH VP 7966; UMNH VP 8102; UMNH VP 8123; UMNH VP 8142; UMNH VP 8151; UMNH VP 8229; UMNH VP 8240–8241; UMNH VP 8355; UMNH VP 8397; UMNH VP 8484; UMNH VP 9089; UMNH VP 9103; UMNH VP 9147; UMNH VP 9149; UMNH VP 9149; UMNH VP 9162; UMNH VP 9168; UMNH VP 9180; UMNH VP 9191; UMNH VP 9201; UMNH VP 9212; UMNH VP 9323; UMNH VP 9327; UMNH VP 9366; UMNH VP 9376; UMNH VP 9401; UMNH VP 9470; UMNH VP 9473; UMNH VP 9480; UMNH VP 9500; UMNH VP 9502; UMNH VP 9505; UMNH VP 9514; UMNH VP 9709; UMNH VP 10360; UMNH VP 10386; UMNH VP 10779; UMNH VP 11031; UMNH VP 11463; UMNH VP 12231; UMNH VP 16584–16585 UMNH VP 16605; UMNH VP 21117; UMNH VP 23132; UMNH VPC 481

**USNM**, National Museum of Natural History, (formerly United States National Museum), Smithsonian Institution, Washington, D.C., USA: USNM 4734; USNM 4735; and USMN 544100

**YPM**, Yale Peabody Museum, New Haven, Connecticut, USA: YPM 1890; YPM 1893; YPM 1991-1995; YPM 2010; and YPM 9162

**ZDCM**, Zhucheng Dinosaur Museum, Shandong, China: ZDCM 5000; ZDCM 5001;
